# Identification of Potential Serum Protein Biomarkers and Pathways for Pancreatic Cancer Cachexia Using an Aptamer-Based Discovery Platform

**DOI:** 10.3390/cancers12123787

**Published:** 2020-12-15

**Authors:** Ashok Narasimhan, Safi Shahda, Joshua K. Kays, Susan M. Perkins, Lijun Cheng, Katheryn N. H. Schloss, Daniel E. I. Schloss, Leonidas G. Koniaris, Teresa A. Zimmers

**Affiliations:** 1 Department of Surgery, Indiana University School of Medicine, Indianapolis, IN 46202, USA; ashnaras@iu.edu (A.N.); joshkays@iupui.edu (J.K.K.); schlosskn@upmc.edu (K.N.H.S.); schlossde@upmc.edu (D.E.I.S.); lkoniari@iu.edu (L.G.K.); 2 Department of Medicine, Indiana University School of Medicine, Indianapolis, IN 46202, USA; safi.shahda@lilly.com; 3 Simon and Bren Comprehensive Cancer Center, Indiana University, Indianapolis, IN 46202, USA; sperkin1@iupui.edu; 4 Department of Biostatistics, Indiana University School of Medicine, Indianapolis, IN 46202, USA; Lijun.Cheng@osumc.edu; 5 Indiana Center for Musculoskeletal Health, Indianapolis, IN 46202, USA; 6 Department of Otolaryngology—Head & Neck Surgery, Indiana University School of Medicine, Indianapolis, IN 46202, USA; 7 Department of Biochemistry and Molecular Biology, Indiana University School of Medicine, Indianapolis, IN 46202, USA; 8 Richard L. Roudebush Veterans Administration Medical Center, Indianapolis, IN 46202, USA

**Keywords:** pancreatic adenocarcinoma, cachexia, biomarkers, humans, neoplasms, proteome, weight loss, paracrine communication

## Abstract

**Simple Summary:**

Patients with pancreatic cancer and other advanced cancers suffer from progressive weight loss that reduces treatment response and quality of life and increases treatment toxicity and mortality. Effective interventions to prevent such weight loss, known as cachexia, require molecular markers to diagnose, stage, and monitor cachexia. No such markers are currently validated or in clinical use. This study used a discovery platform to measure changes in plasma proteins in patients with pancreatic cancer compared with normal controls. We found proteins specific to pancreatic cancer and cancer stage, as well as proteins that correlate with cachexia. These include some previously known proteins along with novel ones and implicates both well-known and new molecular mechanisms. Thus, this study provides novel insights into the molecular processes underpinning cancer and cachexia and affords a basis for future validation studies in larger numbers of patients with pancreatic cancer and cachexia.

**Abstract:**

Patients with pancreatic ductal adenocarcinoma (PDAC) suffer debilitating and deadly weight loss, known as cachexia. Development of therapies requires biomarkers to diagnose, and monitor cachexia; however, no such markers are in use. Via Somascan, we measured ~1300 plasma proteins in 30 patients with PDAC vs. 11 controls. We found 60 proteins specific to local PDAC, 46 to metastatic, and 67 to presence of >5% cancer weight loss (FC ≥ |1.5|, *p* ≤ 0.05). Six were common for cancer stage (Up: GDF15, TIMP1, IL1RL1; Down: CCL22, APP, CLEC1B). Four were common for local/cachexia (C1R, PRKCG, ELANE, SOST: all oppositely regulated) and four for metastatic/cachexia (SERPINA6, PDGFRA, PRSS2, PRSS1: all consistently changed), suggesting that stage and cachexia status might be molecularly separable. We found 71 proteins that correlated with cachexia severity via weight loss grade, weight loss, skeletal muscle index and radiodensity (*r* ≥ |0.50|, *p* ≤ 0.05), including some known cachexia mediators/markers (LEP, MSTN, ALB) as well as novel proteins (e.g., LYVE1, C7, F2). Pathway, correlation, and upstream regulator analyses identified known (e.g., IL6, proteosome, mitochondrial dysfunction) and novel (e.g., Wnt signaling, NK cells) mechanisms. Overall, this study affords a basis for validation and provides insights into the processes underpinning cancer cachexia.

## 1. Introduction

Cancer cachexia is a multifactorial paraneoplastic syndrome characterized by severe loss of muscle and fat, leading to overall weight loss [1,2,3]. Up to 85% of patients with pancreatic ductal adenocarcinoma (PDAC) are affected by this debilitating condition and up to 70% of newly diagnosed cases of pancreatic cancer present with weight loss, low muscle mass or clinically defined cachexia [4,5,6]. Cachexia is a powerful predictor of mortality in pancreatic cancer. Patients with cachexia exhibit shorter overall survival after surgery for PDAC [7] and after neoadjuvant chemotherapy [8]. As well, among patients treated with folfirinox for PDAC, cachexia phenotype associates with reduced survival among patients with PDAC, even when controlling for demographics, cancer site, stage, and treatment response [9]. Persistent weight loss and muscle depletion also associate with mortality in chemoradiation [10]. Moreover, cachexia (often conflated with malnutrition) per se is a considerable health burden. Unintentional weight loss associates with poor patient psychosocial well-being, self-esteem and relationships with others [11], and malnutrition/cachexia is a chief cause of hospitalization among U.S. patients with pancreatic cancer, with a 12-month cumulative hospitalization incidence of 23.0% [12]. This was the second greatest indication for non-tumor/pancreas related hospitalization after cytopenia (30.1%) and far greater than chemotherapy-related toxicity (0.7%). Related and contributing to cachexia, problems of nausea/vomiting and gastrointestinal motility comprised 20.8% of hospitalizations. Thus, cachexia contributes greatly to morbidity and mortality in pancreatic cancer.

While reduced food intake and absorption is a recognized contributor to cachexia in patients with pancreatic cancer, cachexia is an active, catabolic process characterized by ongoing inflammation [13]. Feeding of adequate calories does not maintain weight and weight loss often proceeds more rapidly than in conditions of starvation [14]. Moreover, pre-clinical models demonstrate that weight loss results from disordered metabolism due to host-tumor interactions, and not merely from reduced caloric intake [15,16,17,18,19,20]. While there is evidence for efficacy of specific molecular interventions in pre-clinical models e.g., [16,17,21,22,23,24,25], currently there are no FDA-approved, effective therapies for cancer cachexia [26]. This great unmet clinical need is due in part to lack of robust drug development tools, including biomarkers, as well as lack of consensus on appropriate clinical trial inclusion criteria and endpoints.

The consensus definition of cancer cachexia is weight loss greater than 5%, or weight loss greater than 2% in individuals with body-mass index [BMI] < 20 kg/m^2^ or with low skeletal muscle mass (sarcopenia) [1]. This definition has accelerated research in cachexia, but it is retrospective, relies upon prior knowledge of body weight or patient self-reporting, and does not discriminate stages/severity of cachexia. Other frameworks for identifying patients with cachexia include BMI, percentage weight loss, cachexia weight loss grade—a combination of BMI and history of weight loss, skeletal muscle index, and skeletal muscle quality or myosteatosis, often measured by radiodensity [3,27]. These metrics generally relate to toxicities and mortality in cancer and have been useful for stratifying risk.

Currently there are no validated cellular or molecular biomarkers of cancer cachexia, including no prognostic biomarkers to predict which patients will suffer from cachexia. If predictive biomarkers were established, cachexia risk might be detected during the pre-cachectic period when patients have not yet developed overt weight loss or tissue wasting. Intervention at this early stage might alter cachexia progression. Moreover, there are no validated biomarkers to stage cachexia severity at presentation or during progression of disease, hindering quantitative assessment of interventions. Finally, there are no prognostic markers of cachexia that might reveal patients who are likely to respond to therapy. With appropriate biomarkers, we would have the opportunity to identify, stratify, treat, and monitor patients earlier and more precisely than in current clinical trials, optimizing the potential for therapeutic benefit.

Identification of biomarkers also furthers understanding of mechanisms of cachexia. Preclinical models demonstrate that cachexia is driven by multiple biological pathways, particularly inflammatory cytokines and chemokines, but also growth factors, neuropeptides, lipids, miRNAs, and exosomes. All these pathways ultimately signal on multiple organ systems, including the central nervous system, hematopoietic system, liver, gut, heart and tumor, to produce changes in behavior, appetite, energy expenditure, absorption, metabolism, immunity and inflammation that ultimately produce adipose lipolysis and skeletal muscle catabolism [22]. Discovering how these interactions are mediated requires discovery approaches in patients.

To address both discovery of potential biomarkers and discovery of molecular mechanisms, we carried out an aptamer-based proteomics analysis of plasma from patients with pancreatic ductal adenocarcinoma, a tumor type associated with high rates and severity of cachexia. Here we identify (i) differentially expressed proteins based on cancer stage (local or metastatic) vs. controls, (ii) serum proteins that correlate with cachexia-related variables, as determined by the consensus definition [1], cancer weight loss grade [3], percentage weight loss and skeletal muscle index, (iii) the ontological functions, canonical pathways and upstream regulators associated with the cachexia-associated proteins, and (iv) protein co-expression networks that uncover novel pathways in PDAC cachexia. This approach demonstrates little overlap between cancer-associated and cachexia-associated proteins and identified several known cachexia-associated proteins and pathways as well as many new ones. Overall, our data provide candidate proteins and pathways for further validation and functional analysis in PDAC cachexia.

## 2. Results

### 2.1. Patient Demographics/Clinical Characteristics and SOMAscan Quality Control Results

We studied plasma from 30 patients with confirmed pancreatic ductal adenocarcinoma (PDAC) and 11 controls. Age was significantly different between PDAC (67.1 ± 11.4 years) and controls (49.2 ± 14.7). PDAC patients were nearly evenly split for sex, while controls were eight females and three males. Given these imbalances between groups, values were statistically adjusted for age and sex for all cachexia-related comparisons. Markers of wasting or cachexia were evident in the PDAC group vs. controls. Body mass index (BMI), weight loss, cancer weight loss grade (CWLG) [3], skeletal muscle index (SMI), and sarcopenia status [27] were all significantly different between PDAC patients and controls, although skeletal muscle radio-density (SMD) and total adipose index (TAI) were not. Overall, among patients with PDAC there were no differences between sexes except that men had greater TAI (*p* = 0.0033). Average weight loss among patients with PDAC was 11.9 ± 8.1%; this is considerably more severe than the clinical definition of cachexia (>5% weight loss) and more than the 8% cutoff associated with low survival in patients presenting with lung or gastrointestinal cancers [27]. Mean skeletal muscle index for women with PDAC was 39.6 ± 4.2 cm^2^/m^2^, also considerably lower than the threshold of 41 cm^2^/m^2^ associated with low survival in women [27]. Average skeletal muscle density was at or below thresholds associated with mortality (less than 41 for males and 33 for females) for both controls and patients with PDAC. The clinical characteristics are described in Table 1.

### 2.2. SOMAscan Quality Control and Results by Cancer Stage

**Figure 1:**
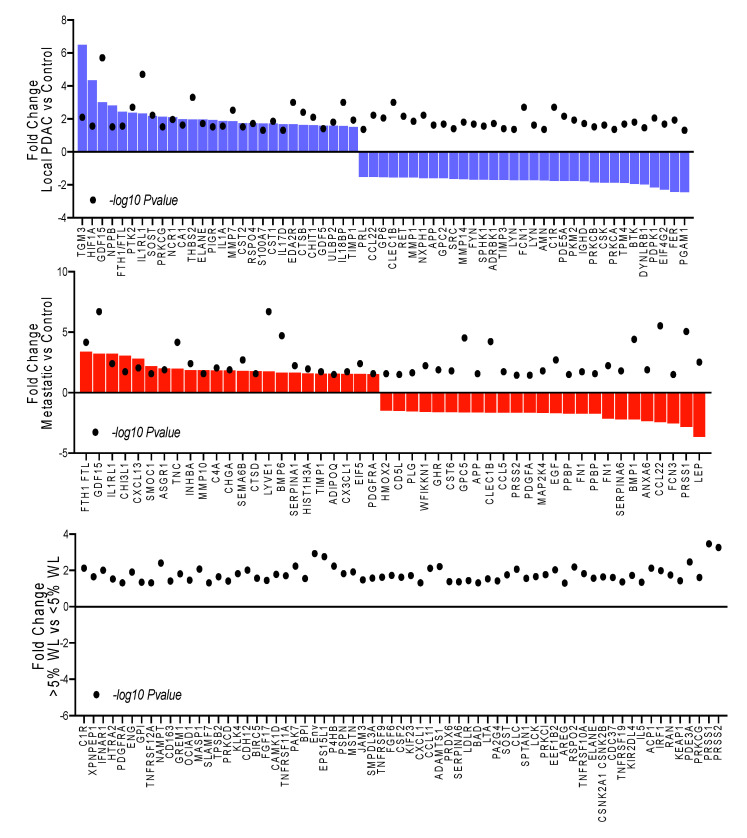
Differentially present proteins in blood of patients with cancer vs. controls. Differentially present proteins by cancer stage, purple = local or locally advanced vs. controls, and red = metastatic vs. controls, or by cachexia status, green = weight loss > 5% vs. <5%, FC ≥ |1.5|, *p* ≤ 0.05.

**Figure 2:**
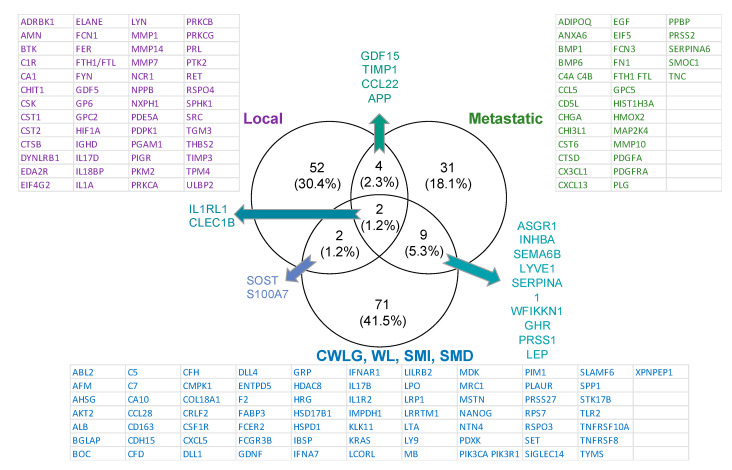
Comparison of proteins specific to cancer vs. cachexia. Venn diagram comparing differentially present proteins in local/locally advanced PDAC patients vs. controls (purple), in metastatic PDAC patients vs. controls (green), and for proteins correlated to cancer weight loss grade (CWLG) and body weight loss (blue).

SOMAscan is an aptamer-based assay that can measure 1310 proteins with high specificity and sensitivity [28]. After quality control and other pre-processing steps, 16 proteins did not satisfy the threshold values. Thus, 1294 proteins were subjected to quantile normalization and used for downstream analysis. We compared cancer patients to non-cancer controls by cancer stage—local and metastatic—and report all differences with fold change (FC) ≥ |1.5|, *p* < 0.05 (Figure 1). In all, 60 proteins were differentially present in patients with local/locally advanced PDAC vs. controls (Table S1), and 46 proteins in patients with metastatic PDAC vs. controls (Table S2). Among the proteins differentially present in cancer vs. control were six common to both local and metastatic PDAC: Up—GDF15, TIMP1, and IL1RL1; Down—CCL22, APP, and CLEC1B (Figure 2).

Ingenuity pathway analysis of proteins differentially present in local PDAC vs. control (*Z*-score ≥ |2.0|) identified the canonical pathways IL-15 Production, Insulin Secretion Signaling, PI3K Signaling in B Lymphocytes, PDGF Signaling, and Reelin Signaling in Neurons and the upstream regulators SP1 (*Z* = −2.214, *p* = 1.38 × 10^−11^) and STAT3 (*Z* = 2.182, *p* = 2.99 × 10^−5^). Disease and biological functions analysis returned annotations related to increased cellular and organismal death and decreased proliferation and movement of blood and tumor cells. In the comparison of metastatic PDAC vs. control, there were no canonical pathways with *Z*-score ≥ |2.0|, although there were many *p*-value significant pathways. Upstream regulators of these proteins were predicted to include HIF1a (−2.000, *p* = 1.94 × 10^−6^), EGF (2.052, *p* = 9.61 × 10^−7^), EGFR (2.102, *p* = 4.35 × 10^−4^), EZH2 (2.200, *p* = 2.76 × 10^−7^). Disease and biofunctional pathways generally related to inhibition of cell signaling and movement of blood and tumor cells. These generally non-overlapping results suggest that mechanisms and manifestations of cancer in peripheral blood could be quite different by cancer stage. The complete lists of pathways are available in Tables S3 and S4.

#### Proteins Associated with Clinical Variables of Cachexia

To identify markers of cachexia, we restricted our analysis to patients with PDAC and compared those meeting a general definition of cachexia, i.e., weight loss of at least 5% during the prior 6 months (*n* = 23) to those with less than 5% or no weight loss (*n =* 6). In this analysis, 67 proteins were differentially present with FC ≥ |1.5|, and unadjusted *p* ≤ 0.05 (Table S5). Comparing this list of cachexia-specific proteins to stage-specific proteins only four were also found to be present in local PDAC—C1R, PRKCG, ELANE, and SOST—and all were oppositely regulated between conditions. In contrast, the 4 proteins common between metastatic PDAC and cachexia, SERPINA6, PDGFRA, PRSS2, and PRSS1, were all changed in the same direction (Figure 1). These data indicate that while cachexia typically associates to the more advanced stage, here cancer stage and cachexia status appear to be largely separable at a molecular level.

Given the disproportionately small number of no-cachexia controls in our dataset (and given that weight loss of even <5% could be both clinically meaningful and indicative of cachexia onset), we next compared blood proteins in cancer patients to cachexia-relevant variables that have been shown to correlate with cancer mortality. These include cancer weight loss grade (CWLG), percentage weight loss, skeletal muscle index (SMI), and skeletal muscle density (SMD). Proteins with correlation values *r* ≥ |0.5| and unadjusted *p* ≤ 0.05 were considered for interpretation [29].

**Figure 3:**
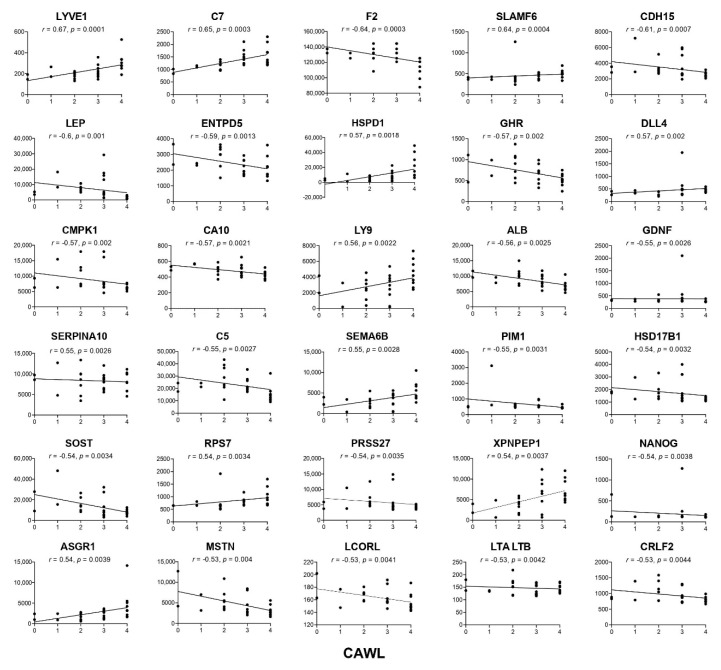
Top 30 proteins correlated with Cancer Weight Loss Grade. The partial Spearman’s rank correlation (*r*) adjusted for age and sex, and *p*-value are given for each protein. *X*-axis indicates cancer weight loss grade (0 = high BMI/low weight loss; 4 = low BMI/high weight loss) and Y axis indicates relative fluorescence units.

Of the 47 proteins associated with CWLG, 28 were negatively correlated and 19 proteins were positively correlated (the top 30 are shown in Figure 3 and the entire list in Table 2). LYVE1, a homolog of CD44, was identified as the top correlated protein with CWLG. Other inflammation-related proteins such as C7, lymphocyte surface antigen LY9, IFNAR1, and IL1RL1 also correlated positively with CWLG, while C5 and F2 correlated negatively. Proteins implicated as mediators or biomarkers of cachexia, including MSTN, INHBA and ALB, were also identified. PH related protein CA10 and stem cell marker NANOG were negatively correlated with CWLG.

**Figure 4:**
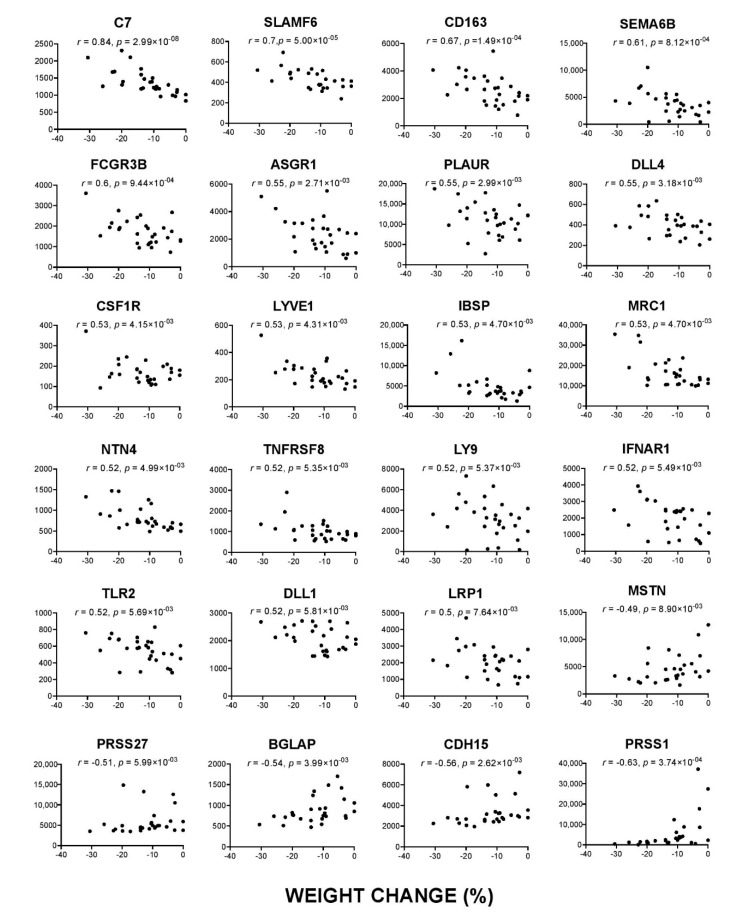
Proteins correlated with weight loss. The partial Spearman’s rank correlation (*r*) adjusted for age and sex, and *p*-value are given for each protein. X-axis indicates percentage weight loss and Y axis indicates relative fluorescence units.

Among the 24 proteins correlated with percentage weight loss, there were 12 which overlapped with CWLG (Figure 4). 19 proteins rose with increasing weight loss, while five fell with weight loss. Complement C7 showed the tightest relationship (*r* = 0.84, *p* = 2.99 × 10^−8^), rising steeply with weight loss. Other pro-inflammatory markers included CD163 (*r* = 0.67, *p* = 0.0001) and CSF1R (*r* = 0.53, *p* = 0.0041), indicating inflammation. Interestingly, IBSP (*r* = 0.53, *p* = 0.0047), an important structural protein in bone matrix and BGLAP (*r* = −0.54, *p* = 0.0039), a bone remodeling protein, correlated with percent weight change. Evidence from animal studies suggest that, along with muscle and fat loss, bone loss and reduced bone strength was also observed in cachexia [30].

We also determined proteins correlated with body composition, including skeletal muscle index (SMI), skeletal muscle density (SMD), and total adipose index (TAI). Low skeletal muscle index (myopenia) is correlated with cancer mortality. The top correlated protein with SMI was gastrin releasing peptide, GRP. Other proteins correlated with SMI include acetylation proteins SET (*r* = 0.56, *p* = 0.0028) and HDAC8 (*r* = 0.51, *p* = 0.0076), inflammatory proteins CFH (*r* = 0.60, *p* = 0.0012) and IL1R2 (*r* = 0.53, *p* = 0.0054) and calcium binding protein S100A7 (*r* = 0.62, *p* = 0.0007). The list of proteins correlated with SMI is presented in Table 3.

**Figure 5:**
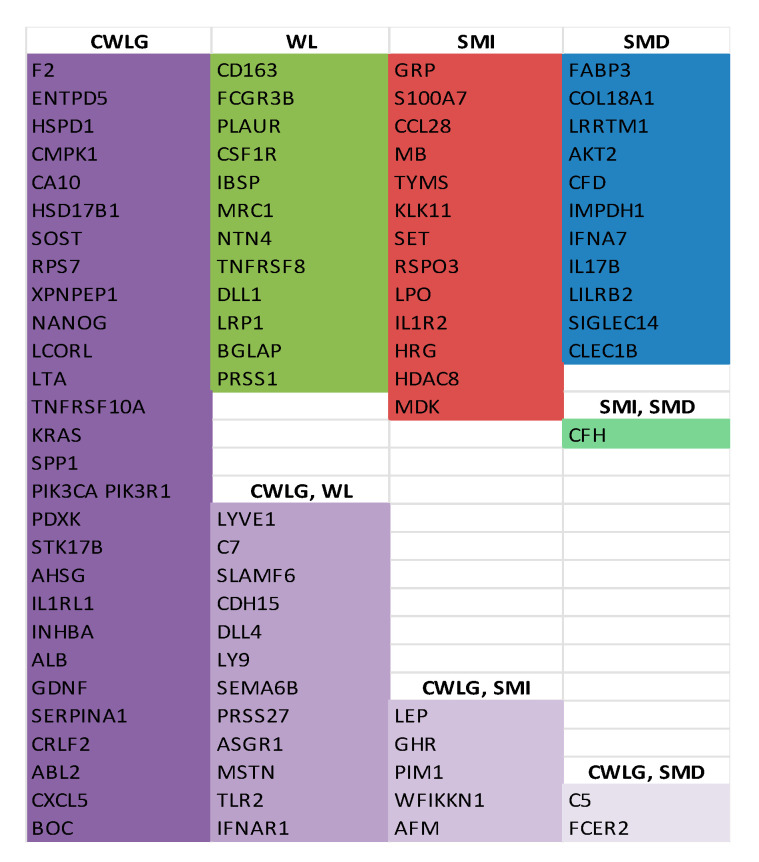
Proteins correlated with cachexia relevant variables. Unique and overlapping proteins identified across the cachexia indices of cancer weight loss Grade (CWLG), weight loss (WL), skeletal muscle index (SMI), skeletal muscle density (SMD), and the combinations.

For skeletal muscle density (SMD), the largest correlation was for FABP3, a fatty acid binding protein involved in fatty acid transport, which increased with decreasing density, likely a reflection of increasing myosteatosis. Proteins associated with inflammation such as CFH (*r* = −0.58, *p* = 0.0018), C5 (*r* = −0.56, *p* = 0.0031), IFNA7 (*r* = −0.55, *p* = 0.0034), IL17B (*r* = 0.53, *p* = 0.0051) and FCER2 (*r* = −0.51, *p* = 0.0073) were also associated with SMD. The complete list of proteins correlated with SMD is reported in Table 4. The list of unique and common proteins identified across the cachexia indices is given in Figure 5.

Total adipose index (TAI) has not been shown to be predictive of mortality in cancer and the role of adipose in cachexia is unclear. Nevertheless, we correlated blood proteins vs. TAI. Leptin (LEP, *r* = 0.85, *p* = 3.13 × 10^−8^), an important molecule in energy homeostasis [31] was the top molecule positively associated with TAI. SFRP1 (*r* = −0.53, *p* = 0.006), a protein associated with increased adiposity, and lipid-associated proteins APOE (*r* = 0.5, *p* = 0.009) and FABP3 (*r* = 0.67, *p* = 0.0002) were also positively associated with TAI. Other proteins include C5 (*r* = 0.69, *p* = 0.0001), C1S (*r* = 0.65, *p* = 0.0003), F9 (*r* = 0.53, *p* = 0.006), CCL28 (*r* = −0.68, *p* = 0.0002), PTN (*r* = −0.52, *p* = 0.007). The complete list of proteins correlated with TAI is presented in Table 5.

Given that our primary goal was to identify cachexia-related biomarkers and not tumor markers per se, we compared proteins differentially present by cancer presence and cancer stage with all those that correlated with the cachexia-relevant variables of CWLG, percentage weight loss, SMI or SMD (Figure 2). This approach enabled identification of markers that might be common to cancer stage and cachexia, as well as markers unique to each condition. Two proteins were common among all comparisons, IL1RL1 and CLEC1B. Two others, SOST and S100A7 were common between local PDAC and cachexia-relevant measures. Metastatic PDAC and cachexia-relevant measures shared 9 unique proteins, ASGR1, INHBA, SEMA6, LYVE1, SERPINA1, WFIKKN1, GHR, PRSS1 and LEP. Given that cachexia severity and frequency increases in advanced disease, such common markers could relate to either metastasis or cachexia or both.

### 2.3. Functional Enrichment of Proteins

**Figure 6:**
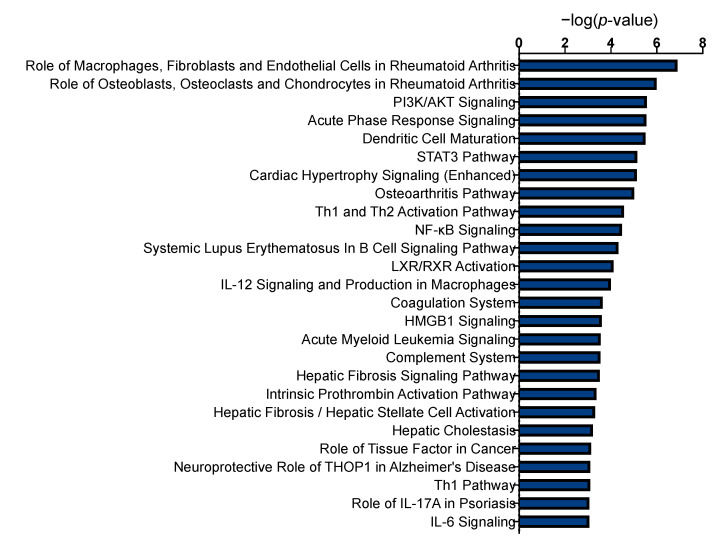
Pathways implicated by proteins differentially present in patients with cachexia. Functional enrichment analysis of proteins correlated with CWLG, weight loss, SMI, and SMD were used for pathway analysis. Pathways with *p* < 0.05 are represented. The X-axis indicates the number of genes in each pathway and Y-axis indicate the pathway names.

Functional enrichment analysis was performed on the proteins that correlated with CWLG, weight loss, SMI, SMD. Ingenuity pathway analysis identified inflammatory pathways in arthritis along with classic cachexia pathways, including PI3K/Akt signaling, acute phase response signaling, STAT3 pathway, NF-kB signaling, coagulation and complement system, and IL-6 signaling pathways (Figure 6). Pathways related to dendritic cell maturation, cardiac hypertrophy and osteoarthritis were also revealed.

### 2.4. Upstream Regulators

**Figure 7:**
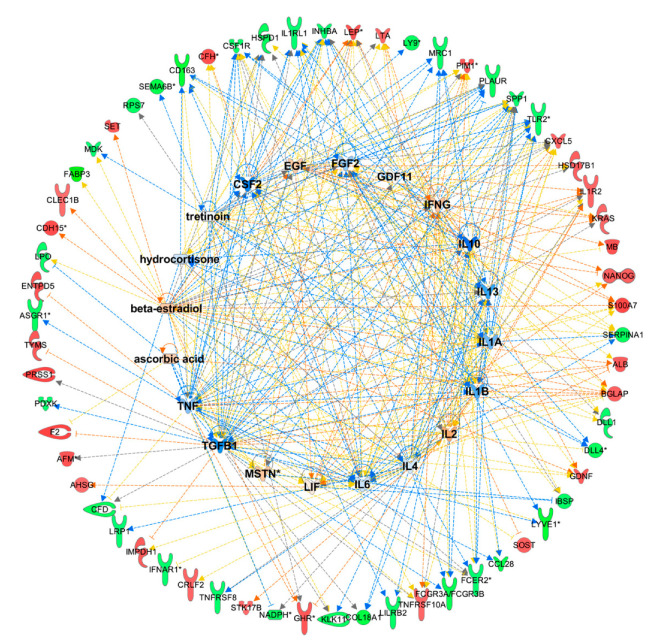
Predicted upstream regulators of proteins differentially present in patients with cancer cachexia. Predicted upstream regulators: The orange and blue color for the upstream regulators in the middle indicate activation and inhibition states, respectively. The red and green color for the downstream proteins indicate positive and negative correlations.

Upstream regulators were predicted from all proteins that correlated with CWLG, weight loss, SMI, and SMD. Predicted regulators include transforming growth factor-beta family members TGF-b, Myostatin, and GDF-11, cytokines including Interleukins (IL) IL-1alpha, IL-1beta, IL-2, IL-4, IL-6, IL-10, IL-13, and leukemia inhibitory factor (LIF), interferon-gamma, tumor necrosis factor (TNF), CSF2, EGF, FGF2, and the endogenous hormones tretinoin, hydrocortisone (cortisol), and beta-estradiol (Figure 7). Several of these upstream regulators such as IL-1 alpha, IL4, IL6, LIF, TNF have known causal roles in cachexia validated using experimental models [13,32,33,34]. The complete list of upstream regulators is given in Table S6.

### 2.5. Protein Co-Expression Analysis

**Figure 8:**
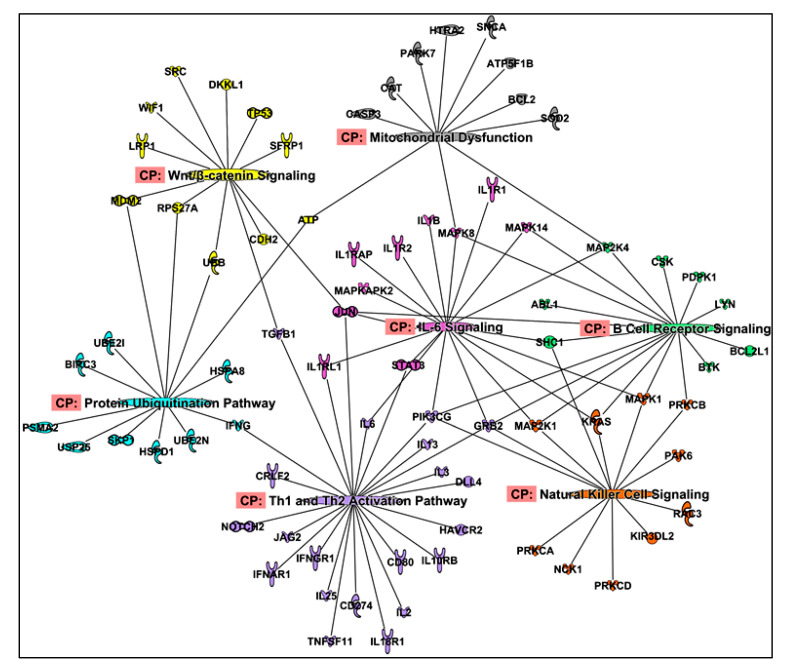
Canonical pathways identified from protein co-expression network. Canonical pathways identified from protein-protein co-expression analysis. Many pathways that were earlier not known in cachexia such as Th1 and Th2 activation pathways, natural killer cell pathways were identified. Some of the well-studied pathways such as IL-6 signaling and protein ubiquitination pathway were also identified.

To identify novel pathways that could potentially be associated with cachexia, we performed a protein co-expression analysis. All 1294 proteins were correlated against each other using Spearman’s correlations. Correlation pairs with *r* value of >0.6 and *p* < 0.05 were considered for the analysis. As CWLG had the highest number of proteins and some proteins associated with SMI, SMD and TAI were also found in CWLG, all the CWLG proteins and their correlated pairs were considered to identify the pathways involved in PDAC cachexia. We identified 1498 correlated pairs for CWLG associated proteins. The non-redundant protein list was imported to IPA and the filters used in IPA were: in prediction category, “experimentally validated and highly predicted pathways” were selected; in the tissue selections, we used “immune cells, skeletal muscle and adipose tissue” to identify canonical pathways. Pathways with *p* < 0.05 included several previously implicated in cachexia including IL-6 signaling, protein ubiquitination, and mitochondrial dysfunction, along with others less explored in cachexia, including B cell receptor signaling, Th1 and Th2 activation pathway, natural killer cell signaling, Wnt/β- catenin signaling, and PTEN signaling (Figure 8). Other pathways including acute phase response, glucocorticoid receptor, STAT3, and BMP signaling pathways were also identified. The complete list of significant canonical pathways is given in Table S7.

## 3. Discussion

Using SOMAscan, an aptamer-based assay, we identified potential circulating protein biomarkers for PDAC cancer stages and cachexia. While studies have identified circulating biomarkers for either pancreatic cancer or cachexia independently, it has never been done simultaneously, a strength of this study. Other strengths of the study include the unbiased discovery approach, the comparison of cancer patients vs. controls, the use of both the canonical, categorical definition of cachexia and also ordinal and continuous measures of CWLG, body weight loss, SMI, and SMD. Weaknesses include a lack of validation cohort and modest sample size, which are appropriate for discovery studies but insufficient for establishing predictive value of any one protein or group of proteins for predicting cachexia status. Despite these limitations, our study revealed that proteins differentially present for local PDAC and metastatic PDAC vs. controls were predominantly non-overlapping indicating that distinct mechanisms may be involved in disease progression. Ultimately such distinctions in circulation may aid in developing stage specific markers.

Although the primary motivation for our study was to find cachexia-specific proteins, we also found proteins present in all conditions and many exclusive to cancer stage. Among the proteins found in all conditions were IL1RL1 and CLEC1B. IL1RL1, also known as IL-33R/ST2, has been linked to tumor growth and progression in mouse models of pancreatic cancer [35]. As well, IL-33 and the decoy receptor soluble IL1RL1/ST2 as measured by ELISA associates with poor survival in patients with advanced PDAC undergoing chemotherapy [36]. CLEC1B has been identified as a potential biomarker of hepatocellular carcinoma [37,38], but to date there are no data in PDAC. Four additional proteins were present in PDAC but did not associate to cachexia status, including GDF15, TIMP1, CCL22, and APP. GDF15 is known to regulate nausea and vomiting and its inhibition slows cachexia in preclinical cancer models [21,25,39]. However, in our small study GDF15 associated with PDAC but not with any metric of cachexia. TIMP1 is reported to be elevated in patients with PDAC [40], but a prior study of TIMP1 measured by ELISA revealed only a modest Spearman correlation with weight loss (*r* = 0.304, *p* = 0.017) [41] consistent with our data. CCL22 correlates with a systemic inflammation response index that predicts survival of patients with PDAC [42], and APP has been linked to tumor growth in experimental cancers, including PDAC [43]. Our confirmatory findings suggest further analysis of these and other cancer stage-specific proteins.

We chose two methods of identifying cachexia-related proteins—the dichotomous approach of <5% vs. >5% weight loss, which resulted in a small group of “no cachexia” patients who might have early cachexia, and correlation analysis with the ordinal or continuous variables of CWLG, weight loss, SMI and SMD. We felt the latter approach to be more discriminating even given our relatively small sample size and thus limit our pathway analysis and discussion to these proteins. Many proteins correlated with CWLG were also correlated with SMI, SMD and TAI. This is expected because both muscle and fat generally decline with increasing CWLG.

Two proteins were common between local PDAC and cachexia, SOST and S100A7. To our knowledge, this is the first report of an association between the WNT inhibitor SOST, or sclerostin, and pancreatic cancer. SOST reduces osteoblastic bone formation and SOST inhibitors are approved for use in women with high risk of osteoporotic fracture. SOST levels by Somascan associate strongly with age [44,45], however in our findings SOST levels declined with increased weight loss severity CWLG (*r* = −0.54, *p* = 0.034), even after adjusting for age. Further investigation of SOST might be warranted. S100A7 or psoriasin was increased in local PDAC but correlated positively with SMI; thus, despite some evidence of association with cancer severity, S100A7 appears uninformative in this context.

Among the nine common proteins between the metastatic PDAC and cachexia-associated discoveries are several with reported roles in both PDAC progression and cachexia. These include INHBA (Activin A) and WFIKKN, an activin inhibitor also known as GASP-2. Activin A associates with PDAC stage and cachexia severity in other studies using different methods and Activin inhibition slows muscle wasting and lengthens survival in experimental models of PDAC [16,46], while GASP-2 over-expression results in hypermuscularity [47]. The anti-activin receptor antibody bimagrumab (BYM 338) has been tested in advanced lung and pancreatic cancer (clinicaltrials.gov identifier NCT01433263), although results have not been published in the literature. Here we observed positive association of INHBA with CWLG (*r* = 0.50, *p* = 0.0077), and negative association of WFIKKN (*r* = −0.52, *p* = 0.0068, consistent with increased Activin activity as a mechanism of cachexia. LYVE1 is reported to be elevated in urine of patients with PDAC, enough to be one of three biomarkers to stratify cancer risk [48], with no data on cachexia. PRSS1 aka trypsin/trypsinogen mutations increase risk of chronic pancreatitis and pancreatic cancer [49] and its pathological activation promotes neoplasia [50]. The ratio of PRSS1 and its inhibitor SERPINA1 or alpha-1-antitrypsin is elevated in patients with PDAC [51], and SERPINA1 deficiency is associated with pancreatitis and cachexia phenotypes [52]. The 3.664 reduction in LEP observed in patients with metastatic PDAC, its negative association with CWLG (*r* = −0.59, *p* = 0.0010) and its positive association with TAI (*r* = 0.85, *p* = 3.13 × 10^−8^) all suggest that LEP tracks with adiposity in PDAC and cachexia. Studies are lacking for the remaining two common proteins, SEMA6B and GHR, in PDAC and cachexia. Given the high relevance of the other identified proteins, however, perhaps they require a careful look.

Among the remainder of cachexia-associated proteins, we find some consistencies with the literature, including a negative association of ALB with CWLG (*r* = −0.56, *p* = 0.0025), consistent with inflammation and albumin as a negative acute phase response protein, and a negative association of MSTN (*r* = −0.53, *p* = 0.0040) potentially tracking with muscle loss (but not SMI), as observed for LEP and adiposity [46,53,54]. Pertinently, the MSTN neutralizing antibody LY2495655 did not improve survival in a Phase 2 trial of 125 patients with stage 2–4 pancreatic cancer, and indeed treatment trended poorer survival [55]. While IL-6, another often implicated biomarker in cachexia was not differentially present, upstream regulator prediction and protein co-expression network analysis both identified IL-6 with high significance [56,57]. IL-6 and STAT3 were also positively correlated with CWLG in our study, although below the threshold of 0.50 correlation coefficient set for significance. These consistencies provide confidence in the relevance of novel proteins identified here.

Proteins identified in circulation such as FABP3, MB were also identified in skeletal muscle proteome studies [58,59]. Other highly correlated proteins included coagulation factors C7, F2, and C5, the lymphocyte surface antigen LY9, and IL1RL1, suggesting inflammation. As well, we identified several proinflammatory cytokines such as IL-1 alpha, IL4, IL6, LIF, TNF which have been implicated in cancer cachexia [13,32,33,34].

Our findings suggest that inflammation is a key driver of PDAC cachexia. Functional enrichment and co-expression analysis identified B cell receptor signaling, Th1, Th2 activation pathways and natural killer cell signaling pathways, which are novel cachexia associated pathways identified from the aptamer assay. Other pathways which were previously reported in cachexia such as IL-6 signaling [60], STAT3 signaling [60,61], protein ubiquitination pathway [62] and mitochondrial dysfunction [63] were also identified.

With cachexia being a manifestation of complex host-tumor interaction leading to muscle wasting and impairing muscle regeneration [64], it is recognized that along with myogenic factors, immune cells have a crucial role in remodeling and regenerating skeletal muscle [65]. The presence of immune cells in healthy skeletal muscle is rare. However, in an injured muscle, the concentration of immune cells increases many fold [66]. T cells were shown to be involved in skeletal muscle regeneration after injury [67]. While Th1 has a proinflammatory effect in recruiting neutrophils and monocytes to the damaged site, Th2 cells promote anti-inflammatory response and myoblast fusion [68]. Therefore, balance between Th1 and Th2 signaling may be crucial for proper muscle regeneration. As the inflammatory status in cancer cachexia is shifted more towards proinflammatory than anti-inflammatory [69], it remains to be studied if the same mechanism is observed in Th1 and Th2. Alongside, we identified two coagulation proteins F2 and F9 to be correlated with CWLG. Coagulation imbalance leading to excessive thrombosis is one of the complications seen in patients with advanced PDAC [70]. In C26 mouse model of cancer cachexia, hypercoagulation was observed due to partially elevated inflammatory cytokine levels, including IL-6 [71]. In our study, we found F2 to be negatively correlated with CWLG and F9 to be positively correlated with TAI.

One of the common links between Th1, Th2 pathways and the coagulation proteins F2 and F9 is IL-6. IL-6 is one of the well-studied biomarkers in cancer cachexia [72,73]. IL-6 signaling is one of the significant pathways identified in this study and has extensively been studied for its role in PDAC cachexia, and as a target for cancer therapy [74]. From our pathway network analysis (Figure 8), IL-6 was shown to be involved in Th1 and Th2 activation pathway, and can simultaneously inhibit Th1 polarization and promote Th2 differentiation [75]. Increased levels of IL-6 also causes muscle and fat wasting in mouse models of cachexia [76]. These inferences suggest the diverse and critical role of IL-6 in cachexia. Therefore, anti-IL-6 therapy would be an interesting option to target and see if it can attenuate tumor mass, thereby reducing muscle and fat wasting.

This is the first report on the expression of serum protein biomarkers for myosteatosis. Presence of myosteatosis in patients with cancer severely impacts survival [77]. However, the mechanism through which fat infiltrates muscle in cancer remains to be elucidated. It remains to be seen if FABP3, a fatty acid transport protein correlated here with SMD may potentially be involved in this process. Additional known inflammatory mediators (ALB, TLR2), and signaling molecules of cachexia (INHBA, MSTN, LEP) were identified in our study. In concordance with what has previously been reported, myostatin and albumin are negatively correlated with CWLG in our study [46,78]. Many of the pathways identified from skeletal muscle in earlier studies using experimental models of cachexia have been confirmed in our study using serum samples. This approach could allow us to bridge the gap in understanding the pathophysiology of human PDAC cachexia and accelerate drug development for this devastating condition.

Of note, our upstream analysis identified several TGF-beta superfamily of proteins such as MSTN, GDF11, GDF15, TGF-β suggesting their important roles in cancer and in cachexia, offering a window for therapeutic interventions [79]. Similar to targeting the activin pathway, it would be interesting to explore these common proteins as therapeutic targets to understand if they can reduce tumor burden and also cachexia.

The study identified novel pathways and upstream regulators that requires further investigation in the context of cachexia. Aberrant Wnt/β-catenin signaling causes a shift in muscle fiber type through the interaction of Wnt3a with FOXO1 in chronic heart failure in mice [80]. While FOXO1 activation has a causal role in muscle atrophy [32], it would be interesting to study the effects of targeting Wnt signaling in muscle atrophy. The novel upstream regulators include FGF2 and CSF2 and drugs such as tretinoin, ascorbic acid and beta-estradiol. While FGF2 promotes satellite cell proliferation [81] and beta estradiol deficiency causes muscle weakness in female [82], the role of FGF2 and beta estradiol along with CSF2 and other drugs identified as upstream regulators needs further investigation.

To understand the disease trajectory of cachexia, collecting serial biopsies at different time points would enable us to better understand the change in muscle microenvironment, however, obtaining skeletal muscle biopsies from cancer patients is an invasive procedure and has, to date been difficult to perform. The barriers such as access to muscle samples, the advanced stage of patients with cancer, the focus on cancer therapy and its toxicity have contributed to the slow progress in understanding human cachexia. Alternatively, future studies should aim at collecting liquid biopsies at different time points which may aid in stratifying biomarkers based on the severity of the cancer and cachexia. Further, there are studies to suggest that many of the molecules involved in gene expression, post-transcriptional gene regulatory mechanisms (microRNA and other small non-coding RNA) can be captured using plasma and serum from cancer patients [83,84]. This remains relatively unexplored for cancer cachexia. These observations suggest that liquid biopsies could prove to be a powerful source for biomarker discovery and to understand the human biology of cachexia. Hence, identifying pathways that are enriched in humans, followed by validating in suitable model systems which can capture the heterogeneity of cachexia to an extent could prove to be a powerful strategy for bench to bedside approach.

## 4. Materials and Methods

### 4.1. Recruitment of Study Participants

This was a prospective, observational study that was approved by the Indiana University Institutional Review Board (IRB) (protocol number 1312105608). The study participants diagnosed with either local PDAC or metastatic were recruited from Indiana University Hospital between the years 2015 and 2017. Written informed consent was obtained from patients for blood and clinical data collections. Patients had to be >18 years of age, provide informed consent, had confirmed PDAC—classified as either advanced group or local PDAC (surgical group). Study procedures including collecting blood samples and clinical data were coordinated to meet the standard of care procedures per the treating physician’s discretion. Patients were excluded if they had known HIV or other active malignancies other than PDAC. The collected blood was stored in −80 °C until further use. A total of 30 patients with PDAC, including 19 localized PDAC and 11 with metastatic disease were included in this study. All experiments were performed in accordance with the IRB protocol.

### 4.2. Assessment of Clinical Variables for Cachexia

Weight loss information over the preceding 6 months was collected using the Patient-Generated Subjective Global Assessment. Cancer weight loss grade (CWLG), an ordinal classification of history of percent weight loss and BMI across five grades, was determined for all patients using the Martin et al. classification [3]. The five grades of BMI and percent weight loss are <20.0, 20.0 to 21.9, 22.0 to 24.9, 25.0 to 27.9, and ≥28.0 kg/m^2^ and −2.5% to −5.9%, −6.0% to −10.9%, −11.0% to −14.9%, ≥−15.0%, and weight stable respectively [3].

CT scans obtained as part of standard of care follow up and in intervals of every 8–12 weeks were retrieved for body composition analysis utilizing SliceOMatic software according to the method of Baracos; the third lumbar vertebrae were used as a standard landmark to measure the skeletal muscle and total adipose components [3,27]. Skeletal muscle index (SMI) and total adipose index (TAI) were calculated by normalizing the skeletal muscle and adipose tissue area to their stature (cm^2^/m^2^) [3,27,85]. Skeletal muscle density was measured as average Hounsfield units across. Sarcopenia status of these patients was calculated based on Martin et al. classification [27].

One participant could not be scored for cancer weight loss grade (CWLG) and two participants did not have CT scans from which to determine SMI, SMD and TAI. Therefore, 29 subjects were used for CWLG correlation analysis and 28 subjects for body composition correlation analysis.

### 4.3. Protein Measurements Using SOMAscan

Serum samples were subjected to SOMAscan proteomic assay [28]. In brief, SOMAscan is an aptamer-based technology that utilizes single-stranded DNA aptamers chemically modified to enhance the binding to protein epitope with high specificity. Each of the 1310 proteins measured in serum by the version of the SOMAscan assay performed in this study has its own targeted SOMAmer reagent, which is used as an affinity binding reagent and quantified on a custom hybridization chip. Cases associated with local PDAC and metastatic group were randomly assigned to plates within each assay run along with a set of calibration and normalization samples. No identifying information was available to the laboratory technicians operating the assay. Intrarun normalization and interrun calibration was performed according to SOMAscan assay data quality-control procedures as defined in the SomaLogic good laboratory practice quality system.

Among the 1310 proteins assayed, several proteins had more than one probe. 6 proteins did not pass QC, leaving 1294 proteins for analysis. The output for every protein from the array is given as relative fluorescence units (RFU), which is proportional to the amount of target protein present in the sample. Hybridization normalization was performed to reduce the technical variation. The data was then median normalized to remove any variation between samples and to account for any variation in the assay. After the preprocessing steps, quantile normalization was performed across all samples for proteins for downstream analyses. The raw data and normalized data are submitted to GEO (ncbi.nlm.nih.gov/geo/; Accession number: GSE119483).

### 4.4. Statistical Analyses

Proteins were tested for normality by group (No cancer/Metastatic/Local PDAC) via Kolmogorov-Smirnov test (*p*-value < 0.05 indicating non-normality). Even after taking the natural log, many proteins were non-normal (18% for controls, 36% for Local PDAC, and 23% for Metastatic); thus, non-parametric approaches were utilized in analyses. Groups comparisons were made using Wilcoxon Mann-Whitney U-tests. Proteins with >1.5-fold change and *p* < 0.05 were considered for downstream analysis. For cachexia comparisons among cancer patients, partial Spearman’s correlations adjusted for age and sex were computed to correlate protein expression with CWLG (treated as an ordinal variable), SMI, SMD and TAI (treated as continuous variables). GraphPad Prism 7 (GraphPad Software, Inc., La Jolla, CA, USA) and R statistical program (R Core Team, Vienna, Austria) were used for statistical analyses and visualization.

### 4.5. Identification of Upstream Regulators of Proteins Associated with Cachexia

Proteins correlated with CWLG, SMI, and SMD with effect size ≥0.5 and *p* < 0.05 were used for prediction of upstream regulators using Ingenuity Pathway Analysis (IPA, Version 2.3, November 2018, Qiagen, Redwood City, CA, USA).

### 4.6. Protein Co-Expression Analysis to Identify Novel Pathways Associated with Cachexia

To identify novel pathways that can potentially be associated with PDAC cachexia, in-silico protein co-expression analysis was performed using Spearman correlation. All 1294 protein values in patients with PDAC were correlated against each other and interactions with strong correlation value of 0.6 (*r*-value) and *p* < 0.05 were further considered for interpretation. Pathways of the co-expression network were identified using Ingenuity Pathway Analysis.

## 5. Conclusions

We have identified novel circulating protein biomarkers associated with human PDAC and PDAC cachexia. We have also identified previously reported markers along with novel biomarkers. Our data suggest that cancer stage and cachexia stage are molecularly different. We report inflammatory and signaling pathways that were not previously described in cachexia. It would also be of great interest to explore whether these biomarkers are disease specific by evaluating them in other malignancies associated cachexia.

## Figures and Tables

**Table 1:** Clinical characteristics of patients included in this cohort.

Characteristics	Control (*n* = 11)	PDAC (*n* = 30)	*p*-Value
Age ^a^	49.2 ± 14.7	67.1 ± 11.4	0.001
Female	54.6 ± 12.7	70.5 ± 11.7	0.015
Male	34.7 ± 8.5	64.2 ± 10.3	0.002
Gender ^b^			0.138
Female	8	14	
Male	3	16	
Cancer Stage			
Local/locally advanced		19	
Metastatic		11	
BMI (kg/m^2^) † ^a^	33.2 ± 7.8	28.0 ± 7.1	0.023
Female	33.4 ± 8.8	26.1 ± 4.5	0.042
Male	32.7 ± 3.8	29.7 ± 8.5	0.301
Change in weight (% mean) ‡ ^a^	−0.9 ± 9.1	11.9 ± 8.1	0.001
Female	0.2 ± 10.4	−10.8 ± 8.3	0.013
Male	−4.0 ± 0.7	−12.9 ± 7.8	0.129
Weight Loss Grade ^c^			0.02
Grade 0	3	3	
Grade 1	4	2	
Grade 2	3	7	
Grade 3	1	8	
Grade 4	0	9	
Skeletal muscle index (cm^2^/m^2^) * ^a^			
Female	51.5 ± 9.3	39.6 ± 4.2	0.013
Male	59.9 ± 4.4	46.1 ± 12.2	0.1
Skeletal muscle density (HU) ^a^			
Female	30.8 ± 7.7	27.9 ± 9.5	0.99
Male	40.3 ± 0.9	33.2 ± 9.8	0.427
Total Adipose Index (cm^2^/m^2^) * ^a^			
Female	183.8 ± 82.1	111.1 ± 52.2	0.140
Male	177.4 ± 54.8	143.7 ± 83.2	0.301
Sarcopenia Status (yes/no) § ^c^	2/9	20/8	0.0040
Female	2/6	6/7	0.399
Male	0/3	12/6	0.0632
Information not available	-	2	

Values are indicated as mean ± standard deviation. † BMI calculated as weight (kg)/height (m)^2^. ‡ Weight loss in prior 6 months calculated as ((current weight in kg) − (weight 6 months ago in kg)/weight 6 months ago in kg)) × 100%. Negative values indicate weight loss. * Skeletal and total adipose indices were calculated as cross-sectional area (cm^2^)/height (m)^2^. Cancer Weight Loss grade was defined as history of weight loss combined with BMI. § Sarcopenia status was assigned using Martin et al. classification [27]. Statistically significant differences were not observed between males and females. a—Mann-Whitney U test; b—Chi-square test; c—Fisher’s exact test.

**Table 2:** Proteins correlated with CWLG.

Protein Name	*r*-Value	*p*-Value	Protein Name	*r*-Value	*p*-Value	Protein Name	*r*-Value	*p*-Value
LYVE1	0.67	0.0001	C5	−0.55	0.0027	TNFRSF10A	−0.52	0.0056
C7	0.65	0.0003	SEMA6B	0.55	0.0028	KRAS	−0.52	0.0056
F2	−0.64	0.0003	PIM1	−0.55	0.0031	SPP1	0.52	0.0059
SLAMF6	0.64	0.0004	HSD17B1	−0.54	0.0032	PIK3CA PIK3R1	0.52	0.0059
CDH15	−0.61	0.0007	SOST	−0.54	0.0034	PDXK	0.51	0.0063
LEP	−0.6	0.0010	RPS7	0.54	0.0034	TLR2	0.51	0.0064
ENTPD5	−0.59	0.0013	PRSS27	−0.54	0.0035	IFNAR1	0.51	0.0065
HSPD1	0.57	0.0018	XPNPEP1	0.54	0.0037	STK17B	−0.51	0.0065
GHR	−0.57	0.0020	NANOG	−0.54	0.0038	WFIKKN1	−0.52	0.0068
DLL4	0.57	0.0020	ASGR1	0.54	0.0039	FCER2	−0.50	0.0073
CMPK1	−0.57	0.0020	MSTN	−0.53	0.0040	AHSG	−0.50	0.0074
CA10	−0.57	0.0021	LCORL	−0.53	0.0041	IL1RL1	0.50	0.0075
LY9	0.56	0.0022	LTA	−0.53	0.0042	INHBA	0.50	0.0077
ALB	−0.56	0.0025	CRLF2	−0.53	0.0044	AFM	−0.50	0.0077
GDNF	−0.55	0.0026	ABL2	−0.53	0.0047	BOC	0.50	0.0078
SERPINA1	0.56	0.0026	CXCL5	−0.53	0.0048			

Partial Spearman Correlation adjusted for age and gender was calculated for CWLG. All proteins with *r* ≥ 0.5 and *p* < 0.05 are reported. *+* value indicates correlation value.

**Table 3:** Proteins correlated with skeletal muscle index (SMI).

Protein Name	*r*-Value	*p*-Value	Protein Name	*r*-Value	*p*-Value
GRP	0.70	0.0001	SET	0.56	0.0028
S100A7	0.62	0.0007	LEP	0.56	0.0028
CCL28	−0.61	0.0009	RSPO3	−0.56	0.0031
CFH	0.60	0.0012	WFIKKN1	0.54	0.0041
AFM	0.59	0.0012	LPO	−0.53	0.0050
MB	0.59	0.0015	IL1R2	0.53	0.0054
TYMS	0.57	0.0023	HRG	0.52	0.0066
GHR	0.57	0.0025	HDAC8	0.51	0.0076
PIM1	0.57	0.0026	MDK	−0.50	0.0086
KLK11	−0.56	0.0027			

Partial Spearman Correlation adjusted for age and gender was calculated for SMI. All proteins which had *r* ≥ 0.5 and *p* < 0.05 are reported in this table. *r*-value indicates correlation value.

**Table 4:** Proteins correlated with skeletal muscle density (SMD).

Protein Name	*r*-Value	*p*-Value
FABP3	−0.77	3.7000 × 10^−6^
COL18A1	−0.63	0.0006
LRRTM1	−0.61	0.0008
CFH	−0.58	0.0018
AKT2	0.57	0.0025
CFD	−0.57	0.0026
C5	−0.56	0.0031
IMPDH1	0.56	0.0032
IFNA7	−0.55	0.0034
IL17B	0.53	0.0051
LILRB2	−0.53	0.0052
FCER2	−0.51	0.0073
SIGLEC14	−0.51	0.0074
CLEC1B	0.50	0.0086

Partial Spearman Correlation adjusted for age and gender was calculated for SMD. All proteins which had *r* ≥ 0.5 and *p* < 0.05 are reported in this table. *r*-value indicates correlation value.

**Table 5:** Proteins correlated with total adipose index (TAI).

Protein Name	*r*-Value	*p*-Value	Protein Name	*r*-Value	*p*-Value
LEP	0.85	3.13 × 10^−8^	Human-virus	0.56	0.003
C5	0.69	0.0001	CFH	0.54	0.004
CCL28	−0.68	0.0002	TP53	0.54	0.004
FABP3	0.67	0.0002	DKKL1	0.55	0.004
BIRC5	0.65	0.0003	AFM	0.55	0.004
C1S	0.65	0.0003	PLG	0.54	0.005
KLRF1	0.65	0.0003	SFRP1	−0.53	0.006
PIM1	0.65	0.0003	UBE2G2	0.52	0.006
KLK11	−0.61	0.001	F9	0.53	0.006
MDK	−0.61	0.001	KLKB1	0.552	0.006
PRKAA1	0.59	0.001	PTN	−0.52	0.007
HRG	0.6	0.001	LTBR	0.51	0.007
RSPO3	−0.58	0.002	KEAP1	−0.51	0.008
HMGN1	0.58	0.002	APOE	0.5	0.009
LMAN2	0.59	0.002	CCL16	0.5	0.01

Partial Spearman Correlation adjusted for age and gender was calculated for TAI. All proteins which had *r* ≥ 0.5 and *p* < 0.05 are reported in this table. *r*-value indicates correlation value.

## Supplementary Materials

The following are available online at https://www.mdpi.com/2072-6694/12/12/3787/s1, Table S1: List of differentially expressed proteins between local PDAC vs. control patients. Table S2: List of differentially expressed proteins between metastatic PDAC vs. control patients. Table S3: Canonical pathways for local PDAC vs. control. Table S4: Canonical pathways for metastatic PDAC vs. control. Table S5: List of differentially expressed proteins between cancer patients with cachexia vs. no cachexia. Table S6: Predicted upstream regulators of proteins correlated to markers of cancer cachexia. Table S7: Canonical pathways for protein co-expression analysis.
