# Supplementary material for: Identification of Potential Serum Protein Biomarkers and Pathways for Pancreatic Cancer Cachexia Using an Aptamer-Based Discovery Platform
Source: Cancers (Basel). 2020 Dec 15;12(12):3787. doi: 10.3390/cancers12123787 (PMC7765482; doi:10.3390/cancers12123787)
Supplement: Supplementary file 1 [file cancers-12-03787-s001.pdf]

cancers

MDPI

Supplementary Materials

Identification of Potential Serum Protein Biomarkers and Pathways for pancreatic cancer cachexia Using an Aptamer-Based Discovery Platform

Ashok Narasimhan, Safi Shahda, Joshua K. Kays, Susan M. Perkins, Lijun Cheng, Katheryn N. H. Schloss, Daniel E. I. Schloss, Leonidas G. Koniaris and Teresa A. Zimmers

Table S1. List of differentially expressed proteins between Local PDAC vs. control patients.

Protein	Fold change	*p*-value
TGM3	6.493	0.008
HIF1A	4.340	0.028
GDF15	2.996	0.000002
NPPB	2.808	0.031
FTH1/FTL	2.435	0.028
PTK2	2.372	0.002
IL1RL1	2.322	0.00002
SOST	2.188	0.006
PRKCG	2.133	0.031
NCR1	2.107	0.011
CA1	1.977	0.024
THBS2	1.962	0.0005
ELANE	1.958	0.019
PIGR	1.945	0.031
IL1A	1.882	0.028
MMP7	1.849	0.003
CST2	1.739	0.031
RSPO4	1.731	0.019
S100A7	1.711	0.049
CST1	1.708	0.014
IL17D	1.675	0.049
EDA2R	1.675	0.001
CTSB	1.624	0.004
CHIT1	1.616	0.008
GDF5	1.586	0.039
ULBP2	1.580	0.016
IL18BP	1.571	0.001
TIMP1	1.502	0.012
PRL	-1.517	0.044
CCL22	-1.527	0.006
GP6	-1.533	0.009
CLEC1B	-1.547	0.001
RET	-1.548	0.007
MMP1	-1.558	0.014

NXPH1	-1.604	0.006
APP	-1.606	0.024
GPC2	-1.608	0.021
SRC	-1.641	0.039
MMP14	-1.654	0.016
FYN	-1.683	0.021
SPHK1	-1.688	0.028
ADRBK1	-1.691	0.019
TIMP3	-1.693	0.039
LYN	-1.720	0.044
FCN1	-1.723	0.002
LYN	-1.723	0.024
AMN	-1.726	0.044
C1R	-1.758	0.002
PDE5A	-1.763	0.007
PKM2	-1.769	0.012
IGHD	-1.772	0.019
PRKCB	-1.859	0.031
CSK	-1.870	0.024
PRKCA	-1.875	0.044
TPM4	-1.886	0.021
BTK	-1.949	0.016
DYNLRB1	-1.968	0.035
PDPK1	-2.167	0.009
EIF4G2	-2.298	0.021
FER	-2.424	0.012
PGAM1	-2.438	0.049

Table S2. List of differentially expressed proteins between Metastatic group vs control patients.

Protein	Fold change	*p*-value
FTH1 FTL	3.391	0.00007
GDF15	3.217	0.0000002
IL1RL1	3.210	0.004
CHI3L1	3.045	0.019
CXCL13	2.812	0.009
SMOC1	2.184	0.027
ASGR1	2.002	0.013
TNC	1.975	0.00007
INHBA	1.852	0.004
MMP10	1.851	0.027
C4A C4B	1.839	0.009
CHGA	1.838	0.013
SEMA6B	1.800	0.002
CTSD	1.771	0.027
LYVE1	1.766	0.0000002
BMP6	1.635	0.00002
SERPINA1	1.634	0.006
HIST1H3A	1.589	0.011
TIMP1	1.571	0.019
ADIPOQ	1.570	0.032
CX3CL1	1.545	0.019
EIF5	1.544	0.004
PDGFRA	1.511	0.027
HMOX2	-1.500	0.027
CD5L	-1.511	0.032
PLG	-1.547	0.023
WFIKKN1	-1.595	0.006
GHR	-1.608	0.013
CST6	-1.620	0.016
GPC5	-1.631	0.00003
APP	-1.646	0.027
CLEC1B	-1.659	0.00006
CCL5	-1.662	0.019
PRSS2	-1.672	0.037
PDGFA	-1.674	0.037
MAP2K4	-1.686	0.016
EGF	-1.704	0.002
PPBP	-1.731	0.032
FN1	-1.737	0.019
PPBP	-1.739	0.027
FN1	-2.156	0.006
SERPINA6	-2.204	0.016
BMP1	-2.204	0.00004
ANXA6	-2.358	0.013
CCL22	-2.433	0.000003

FCN3	-2.550	0.032
PRSS1	-2.828	0.000009
LEP	-3.664	0.003

Table S3. Canonical pathways for local PDAC vs control.

Ingenuity Canonical Pathways	*p*-value	Molecules
Leukocyte Extravasation Signaling	1.0E-13	BTK,FER,MMP1,MMP14,MMP7,PRKCA,PRKCB,PRKCG,PTK2,SRC,TIMP1,TIMP3
Sperm Motility	4.0E-13	BTK,CSK,FER,FYN,LYN,NPPB,PRKCA,PRKCB,PRKCG,PTK2,RET,SRC
Systemic Lupus Erythematosus In B Cell Signaling Pathway	5.0E-12	BTK,CSK,FYN,IGHD,IL17D,IL1A,LYN,PDPK1,PRKCA,PRKCB,PRKCG,SRC
GP6 Signaling Pathway	2.0E-11	BTK,FYN,GP6,LYN,PDPK1,PRKCA,PRKCB,PRKCG,PTK2
Inhibition of Matrix Metalloproteases	7.2E-10	MMP1,MMP14,MMP7,THBS2,TIMP1,TIMP3
IL-15 Production	8.9E-10	BTK,CSK,FER,FYN,LYN,PTK2,RET,SRC
Tec Kinase Signaling	1.0E-08	BTK,FYN,LYN,PRKCA,PRKCB,PRKCG,PTK2,SRC
Fc Epsilon RI Signaling	2.2E-08	BTK,FYN,LYN,PDPK1,PRKCA,PRKCB,PRKCG
p70S6K Signaling	4.3E-08	BTK,LYN,PDPK1,PRKCA,PRKCB,PRKCG,SRC
HIF1α Signaling	5.8E-08	HIF1A,MMP1,MMP14,MMP7,PKM,PRKCA,PRKCB,PRKCG
Prolactin Signaling	6.6E-08	FYN,PDPK1,PRKCA,PRKCB,PRKCG,PRL
Fcγ Receptor-mediated Phagocytosis in Macrophages and Monocytes	1.6E-07	FYN,LYN,PRKCA,PRKCB,PRKCG,SRC
Insulin Secretion Signaling Pathway	2.1E-07	EIF4G2,FYN,LYN,PRKCA,PRKCB,PRKCG,PRL,SRC
Hepatic Fibrosis Signaling Pathway	4.3E-07	HIF1A,IL1A,IL1RL1,MMP1,PRKCA,PRKCB,PRKCG,PTK2,TIMP1
G-Protein Coupled Receptor Signaling	4.9E-07	FYN,GRK2,PDE5A,PDPK1,PRKCA,PRKCB,PRKCG,SRC
B Cell Receptor Signaling	5.0E-07	BTK,CSK,IGHD,LYN,PDPK1,PRKCB,PTK2
Cholecystokinin/Gastrin-mediated Signaling	6.5E-07	IL1A,PRKCA,PRKCB,PRKCG,PTK2,SRC
RAR Activation	6.9E-07	CSK,MMP1,PDPK1,PRKCA,PRKCB,PRKCG,SRC
G Beta Gamma Signaling	7.6E-07	BTK,PDPK1,PRKCA,PRKCB,PRKCG,SRC
Growth Hormone Signaling	1.1E-06	PDPK1,PRKCA,PRKCB,PRKCG,PRL
Role of Macrophages, Fibroblasts and Endothelial Cells in Rheumatoid Arthritis	1.4E-06	IL1A,IL1RL1,MMP1,PRKCA,PRKCB,PRKCG,SOST,SRC
Erythropoietin Signaling	1.6E-06	PDPK1,PRKCA,PRKCB,PRKCG,SRC
Estrogen Receptor Signaling	2.0E-06	HIF1A,MMP1,MMP14,MMP7,PRKCA,PRKCB,PRKCG,SRC
Gαq Signaling	3.4E-06	BTK,CSK,GRK2,PRKCA,PRKCB,PRKCG
Opioid Signaling Pathway	3.5E-06	FYN,GRK2,LYN,PRKCA,PRKCB,PRKCG,SRC
Cardiac Hypertrophy Signaling (Enhanced)	4.3E-06	IL17D,IL1A,IL1RL1,NPPB,PDE5A,PRKCA,PRKCB,PRKCG,PTK2
Phospholipase C Signaling	4.5E-06	BTK,FYN,LYN,PRKCA,PRKCB,PRKCG,SRC
CXCR4 Signaling	4.7E-06	LYN,PRKCA,PRKCB,PRKCG,PTK2,SRC
FAK Signaling	4.8E-06	CSK,FYN,PDPK1,PTK2,SRC
Neuregulin Signaling	5.0E-06	PDPK1,PRKCA,PRKCB,PRKCG,SRC
VEGF Signaling	5.9E-06	HIF1A,PRKCA,PRKCB,PTK2,SRC
Granulocyte Adhesion and Diapedesis	7.2E-06	CCL22,IL1A,IL1RL1,MMP1,MMP14,MMP7

Hepatic Cholestasis	8.5E-06	IL17D,IL1A,IL1RL1,PRKCA,PRKCB,PRKCG
Virus Entry via Endocytic Pathways	8.5E-06	FYN,PRKCA,PRKCB,PRKCG,SRC
UVC-Induced MAPK Signaling	9.3E-06	PRKCA,PRKCB,PRKCG,SRC
ERK/MAPK Signaling	1.1E-05	FYN,PRKCA,PRKCB,PRKCG,PTK2,SRC
Role of Tissue Factor in Cancer	1.3E-05	FYN,LYN,MMP1,PRKCA,SRC
Thrombin Signaling	1.6E-05	PDPK1,PRKCA,PRKCB,PRKCG,PTK2,SRC
mTOR Signaling	1.7E-05	EIF4G2,HIF1A,PDPK1,PRKCA,PRKCB,PRKCG
Osteoarthritis Pathway	1.8E-05	GDF5,HIF1A,IL1RL1,MMP1,SPHK1,TIMP3
Role of Osteoblasts, Osteoclasts and Chondrocytes in Rheumatoid Arthritis	2.2E-05	IL1A,IL1RL1,MMP1,MMP14,SOST,SRC
ErbB4 Signaling	2.8E-05	PDPK1,PRKCA,PRKCB,PRKCG
PI3K Signaling in B Lymphocytes	3.0E-05	BTK,FYN,LYN,PDPK1,PRKCB
Type II Diabetes Mellitus Signaling	3.4E-05	PDPK1,PKM,PRKCA,PRKCB,PRKCG
Axonal Guidance Signaling	3.4E-05	FYN,MMP1,MMP14,MMP7,PRKCA,PRKCB,PRKCG,PTK2
Macropinocytosis Signaling	4.6E-05	PRKCA,PRKCB,PRKCG,SRC
Role of Pattern Recognition Receptors in Recognition of Bacteria and Viruses	5.0E-05	IL17D,IL1A,PRKCA,PRKCB,PRKCG
VDR/RXR Activation	5.0E-05	IL1RL1,PRKCA,PRKCB,PRKCG
IL-7 Signaling Pathway	5.0E-05	FYN,LYN,PDPK1,PTK2
Chemokine Signaling	5.6E-05	PRKCA,PRKCB,PTK2,SRC
Molecular Mechanisms of Cancer	6.6E-05	FYN,HIF1A,PRKCA,PRKCB,PRKCG,PTK2,SRC
PDGF Signaling	7.4E-05	PRKCA,PRKCB,SPHK1,SRC
GNRH Signaling	8.7E-05	PRKCA,PRKCB,PRKCG,PTK2,SRC
ErbB Signaling	1.0E-04	PDPK1,PRKCA,PRKCB,PRKCG
Nitric Oxide Signaling in the Cardiovascular System	1.3E-04	PDE5A,PRKCA,PRKCB,PRKCG
Apelin Cardiomyocyte Signaling Pathway	1.3E-04	HIF1A,PRKCA,PRKCB,PRKCG
Neuropathic Pain Signaling In Dorsal Horn Neurons	1.4E-04	PRKCA,PRKCB,PRKCG,SRC
Agranulocyte Adhesion and Diapedesis	1.4E-04	CCL22,IL1A,MMP1,MMP14,MMP7
Mechanisms of Viral Exit from Host Cells	1.7E-04	PRKCA,PRKCB,PRKCG
IL-8 Signaling	1.7E-04	PRKCA,PRKCB,PRKCG,PTK2,SRC
HGF Signaling	2.0E-04	PRKCA,PRKCB,PRKCG,PTK2
Apelin Endothelial Signaling Pathway	2.3E-04	HIF1A,PRKCA,PRKCB,PRKCG
nNOS Signaling in Neurons	2.5E-04	PRKCA,PRKCB,PRKCG
Airway Pathology in Chronic Obstructive Pulmonary Disease	2.5E-04	ELANE,IL17D,IL1A,MMP1
Renin-Angiotensin Signaling	2.5E-04	PRKCA,PRKCB,PRKCG,PTK2
14-3-3-mediated Signaling	3.3E-04	PRKCA,PRKCB,PRKCG,SRC
UVB-Induced MAPK Signaling	3.4E-04	PRKCA,PRKCB,PRKCG
Reelin Signaling in Neurons	3.5E-04	APP,FYN,LYN,SRC
Androgen Signaling	4.4E-04	PRKCA,PRKCB,PRKCG,SRC
Colorectal Cancer Metastasis Signaling	5.0E-04	GRK2,MMP1,MMP14,MMP7,SRC
Thrombopoietin Signaling	5.9E-04	PRKCA,PRKCB,PRKCG
Factors Promoting Cardiogenesis in Vertebrates	6.3E-04	NPPB,PRKCA,PRKCB,PRKCG
Calcium-induced T Lymphocyte Apoptosis	6.8E-04	PRKCA,PRKCB,PRKCG
Nur77 Signaling in T Lymphocytes	7.4E-04	PRKCA,PRKCB,PRKCG
Aldosterone Signaling in Epithelial Cells	7.6E-04	PDPK1,PRKCA,PRKCB,PRKCG
eNOS Signaling	7.8E-04	PDPK1,PRKCA,PRKCB,PRKCG
Melatonin Signaling	8.7E-04	PRKCA,PRKCB,PRKCG
Caveolar-mediated Endocytosis Signaling	9.1E-04	FYN,PRKCA,SRC
Glioma Invasiveness Signaling	9.3E-04	PTK2,TIMP1,TIMP3
FcγRIIB Signaling in B Lymphocytes	9.8E-04	BTK,LYN,PDPK1

Germ Cell-Sertoli Cell Junction Signaling	1.0E-03	FER,PDPK1,PTK2,SRC
IL-3 Signaling	1.1E-03	PRKCA,PRKCB,PRKCG
LPS-stimulated MAPK Signaling	1.3E-03	PRKCA,PRKCB,PRKCG
NF-κB Activation by Viruses	1.3E-03	PRKCA,PRKCB,PRKCG
HER-2 Signaling in Breast Cancer	1.3E-03	PRKCA,PRKCB,PRKCG
VEGF Family Ligand-Receptor Interactions	1.3E-03	PRKCA,PRKCB,PRKCG
Hepatic Fibrosis / Hepatic Stellate Cell Activation	1.4E-03	IL1A,IL1RL1,MMP1,TIMP1
Endothelin-1 Signaling	1.4E-03	PRKCA,PRKCB,PRKCG,SRC
Synaptic Long Term Depression	1.5E-03	LYN,PRKCA,PRKCB,PRKCG
Xenobiotic Metabolism CAR Signaling Pathway	1.5E-03	PRKCA,PRKCB,PRKCG,SRC
Adrenomedullin signaling pathway	1.7E-03	CSK,HIF1A,IL1A,PTK2
Gap Junction Signaling	1.7E-03	PRKCA,PRKCB,PRKCG,SRC
CCR5 Signaling in Macrophages	1.9E-03	PRKCA,PRKCB,PRKCG
α-Adrenergic Signaling	2.0E-03	PRKCA,PRKCB,PRKCG
Bladder Cancer Signaling	2.0E-03	MMP1,MMP14,MMP7
Glycolysis I	2.1E-03	PGAM1,PKM
Role of NFAT in Cardiac Hypertrophy	2.3E-03	PRKCA,PRKCB,PRKCG,SRC
T Cell Receptor Signaling	2.6E-03	BTK,CSK,FYN
Paxillin Signaling	2.8E-03	CSK,PTK2,SRC
Glioma Signaling	3.0E-03	PRKCA,PRKCB,PRKCG
GPCR-Mediated Nutrient Sensing in Enteroendocrine Cells	3.1E-03	PRKCA,PRKCB,PRKCG
Huntington's Disease Signaling	3.3E-03	PDPK1,PRKCA,PRKCB,PRKCG
fMLP Signaling in Neutrophils	3.4E-03	PRKCA,PRKCB,PRKCG
Protein Kinase A Signaling	3.7E-03	PDE5A,PRKCA,PRKCB,PRKCG,PTK2
CD28 Signaling in T Helper Cells	3.8E-03	CSK,FYN,PDPK1
CCR3 Signaling in Eosinophils	4.1E-03	PRKCA,PRKCB,PRKCG
Phagosome Formation	4.2E-03	PRKCA,PRKCB,PRKCG
Docosahexaenoic Acid (DHA) Signaling	4.4E-03	APP,PDPK1
P2Y Purigenic Receptor Signaling Pathway	4.4E-03	PRKCA,PRKCB,PRKCG
Synaptic Long Term Potentiation	4.6E-03	PRKCA,PRKCB,PRKCG
Gα12/13 Signaling	4.7E-03	BTK,PTK2,SRC
IL-12 Signaling and Production in Macrophages	4.9E-03	PRKCA,PRKCB,PRKCG
STAT3 Pathway	5.2E-03	IL1A,IL1RL1,SRC
Oncostatin M Signaling	5.6E-03	MMP1,TIMP3
Endocannabinoid Cancer Inhibition Pathway	6.2E-03	HIF1A,PTK2,SRC
Xenobiotic Metabolism General Signaling Pathway	6.2E-03	PRKCA,PRKCB,PRKCG
Corticotropin Releasing Hormone Signaling	6.3E-03	PRKCA,PRKCB,PRKCG
Xenobiotic Metabolism Signaling	6.6E-03	IL1A,PRKCA,PRKCB,PRKCG
Ephrin A Signaling	6.6E-03	FYN,PTK2
Hematopoiesis from Pluripotent Stem Cells	7.2E-03	IGHD,IL1A
Primary Immunodeficiency Signaling	7.4E-03	BTK,IGHD
HOTAIR Regulatory Pathway	8.3E-03	MMP1,MMP14,MMP7
Synaptogenesis Signaling Pathway	8.7E-03	FYN,LYN,SRC,THBS2
Dopamine-DARPP32 Feedback in cAMP Signaling	8.7E-03	PRKCA,PRKCB,PRKCG
EGF Signaling	8.9E-03	PRKCA,SRC
Semaphorin Signaling in Neurons	1.1E-02	FYN,PTK2
PI3K/AKT Signaling	1.1E-02	GDF15,IL1RL1,PDPK1
SPINK1 Pancreatic Cancer Pathway	1.1E-02	CTSB,ELANE
Acute Phase Response Signaling	1.1E-02	C1R,IL1A,PDPK1
Ephrin Receptor Signaling	1.1E-02	FYN,PTK2,SRC
Role of NFAT in Regulation of the Immune Response	1.1E-02	BTK,FYN,LYN

Regulation of Cellular Mechanics by Calpain Protease	1.2E-02	PTK2,SRC
Production of Nitric Oxide and Reactive Oxygen Species in Macrophages	1.3E-02	PRKCA,PRKCB,PRKCG
NRF2-mediated Oxidative Stress Response	1.3E-02	PRKCA,PRKCB,PRKCG
PPARα/RXRα Activation	1.3E-02	IL1RL1,PRKCA,PRKCB
ILK Signaling	1.3E-02	HIF1A,PDPK1,PTK2
Xenobiotic Metabolism PXR Signaling Pathway	1.3E-02	PRKCA,PRKCB,PRKCG
IL-10 Signaling	1.4E-02	IL1A,IL1RL1
GM-CSF Signaling	1.4E-02	LYN,PRKCB
Natural Killer Cell Signaling	1.4E-02	FYN,NCR1,ULBP2
Non-Small Cell Lung Cancer Signaling	1.5E-02	PDPK1,PRKCA
Rapoport-Luebering Glycolytic Shunt	1.5E-02	PGAM1
CREB Signaling in Neurons	1.7E-02	PRKCA,PRKCB,PRKCG
Toll-like Receptor Signaling	1.7E-02	IL1A,IL1RL1
Agrin Interactions at Neuromuscular Junction	1.7E-02	PTK2,SRC
Integrin Signaling	1.8E-02	FYN,PTK2,SRC
cAMP-mediated signaling	2.1E-02	GRK2,PDE5A,SRC
Communication between Innate and Adaptive Immune Cells	2.6E-02	IGHD,IL1A
PPAR Signaling	3.0E-02	IL1A,IL1RL1
IGF-1 Signaling	3.0E-02	PDPK1,PTK2
Role of IL-17A in Psoriasis	3.3E-02	S100A7
iCOS-iCOSL Signaling in T Helper Cells	3.4E-02	CSK,PDPK1
Neuroprotective Role of THOP1 in Alzheimer's Disease	3.6E-02	APP,C1R
Sphingosine-1-phosphate Signaling	3.7E-02	PTK2,SPHK1
p38 MAPK Signaling	3.8E-02	IL1A,IL1RL1
LXR/RXR Activation	4.0E-02	IL1A,IL1RL1
Sirtuin Signaling Pathway	4.0E-02	APP,HIF1A,PGAM1
Glutaryl-CoA Degradation	4.1E-02	CA1
IL-6 Signaling	4.2E-02	IL1A,IL1RL1
PTEN Signaling	4.3E-02	PDPK1,PTK2
Atherosclerosis Signaling	4.3E-02	IL1A,MMP1
Human Embryonic Stem Cell Pluripotency	4.8E-02	PDPK1,SPHK1
Iron homeostasis signaling pathway	4.9E-02	GDF15,HIF1A
Ovarian Cancer Signaling	5.0E-02	MMP7,SRC
Cardiomyocyte Differentiation via BMP Receptors	5.1E-02	NPPB
Inflammasome pathway	5.1E-02	CTSB
Insulin Receptor Signaling	5.1E-02	FYN,PDPK1
Cardiac β-adrenergic Signaling	5.2E-02	GRK2,PDE5A
Aryl Hydrocarbon Receptor Signaling	5.4E-02	IL1A,SRC
Differential Regulation of Cytokine Production in Intestinal Epithelial Cells by IL-17A and IL-17F	5.8E-02	IL1A
Tryptophan Degradation III (Eukaryotic)	5.8E-02	CA1
Apelin Cardiac Fibroblast Signaling Pathway	5.8E-02	SPHK1
Phagosome Maturation	5.9E-02	CTSB,DYNLRB1
Epithelial Adherens Junction Signaling	5.9E-02	FER,SRC
Regulation of eIF4 and p70S6K Signaling	6.3E-02	EIF4G2,PDPK1
Gluconeogenesis I	6.6E-02	PGAM1
Breast Cancer Regulation by Stathmin1	6.8E-02	HIF1A,PRKCA,PRKCB,PRKCG
HMGB1 Signaling	6.9E-02	IL17D,IL1A
Sonic Hedgehog Signaling	7.2E-02	GRK2
Wnt/β-catenin Signaling	7.4E-02	MMP7,SRC

NF-κB Signaling	7.9E-02	IL1A,PRKCB
RhoGDI Signaling	7.9E-02	PRKCA,SRC
Inhibition of Angiogenesis by TSP1	8.5E-02	FYN
Role of JAK2 in Hormone-like Cytokine Signaling	8.5E-02	PRL
IL-17A Signaling in Fibroblasts	8.7E-02	MMP1
B Cell Development	8.9E-02	IGHD
Complement System	9.1E-02	C1R
Role of Hypercytokinemia/hyperchemokinemia in the Pathogenesis of Influenza	1.1E-01	IL1A
tRNA Splicing	1.1E-01	PDE5A
BAG2 Signaling Pathway	1.1E-01	CTSB
IL-23 Signaling Pathway	1.1E-01	HIF1A
Actin Cytoskeleton Signaling	1.1E-01	CSK,PTK2
LPS/IL-1 Mediated Inhibition of RXR Function	1.2E-01	IL1A,IL1RL1
EIF2 Signaling	1.2E-01	EIF4G2,PDPK1
Graft-versus-Host Disease Signaling	1.2E-01	IL1A
Systemic Lupus Erythematosus Signaling	1.2E-01	IL1A,LYN
Amyloid Processing	1.2E-01	APP
Lymphotoxin β Receptor Signaling	1.3E-01	PDPK1
Role of Cytokines in Mediating Communication between Immune Cells	1.3E-01	IL1A
Role of IL-17A in Arthritis	1.3E-01	MMP1
Endometrial Cancer Signaling	1.4E-01	PDPK1
Autophagy	1.5E-01	CTSB
Wnt/Ca+ pathway	1.5E-01	PRKCA
ErbB2-ErbB3 Signaling	1.6E-01	PDPK1
Remodeling of Epithelial Adherens Junctions	1.6E-01	SRC
Small Cell Lung Cancer Signaling	1.7E-01	PTK2
ERK5 Signaling	1.7E-01	SRC
Ephrin B Signaling	1.7E-01	PTK2
Estrogen-Dependent Breast Cancer Signaling	1.7E-01	SRC
Hypoxia Signaling in the Cardiovascular System	1.7E-01	HIF1A
TREM1 Signaling	1.8E-01	IL1RL1
Angiopoietin Signaling	1.8E-01	PTK2
GDNF Family Ligand-Receptor Interactions	1.8E-01	RET
Neurotrophin/TRK Signaling	1.8E-01	PDPK1
Antiproliferative Role of Somatostatin Receptor 2	1.8E-01	SRC
Dopamine Receptor Signaling	1.8E-01	PRL
Neuroinflammation Signaling Pathway	1.8E-01	APP,PRKCG
Thyroid Cancer Signaling	1.9E-01	RET
BEX2 Signaling Pathway	1.9E-01	HIF1A
IL-17 Signaling	1.9E-01	TIMP1
FLT3 Signaling in Hematopoietic Progenitor Cells	1.9E-01	PDPK1
Renal Cell Carcinoma Signaling	1.9E-01	HIF1A
Role of MAPK Signaling in the Pathogenesis of Influenza	1.9E-01	PRKCA
Apelin Adipocyte Signaling Pathway	1.9E-01	HIF1A
TR/RXR Activation	2.0E-01	HIF1A
FGF Signaling	2.0E-01	PRKCA
Regulation Of The Epithelial Mesenchymal Transition In Development Pathway	2.0E-01	HIF1A
Xenobiotic Metabolism AHR Signaling Pathway	2.0E-01	IL1A
Ceramide Signaling	2.1E-01	SPHK1

RANK Signaling in Osteoclasts	2.1E-01	SRC
CTLA4 Signaling in Cytotoxic T Lymphocytes	2.1E-01	FYN
Regulation of IL-2 Expression in Activated and Anergic T Lymphocytes	2.1E-01	FYN
Altered T Cell and B Cell Signaling in Rheumatoid Arthritis	2.1E-01	IL1A
Prostate Cancer Signaling	2.1E-01	PDPK1
IL-1 Signaling	2.1E-01	IL1A
Th17 Activation Pathway	2.1E-01	HIF1A
Glucocorticoid Receptor Signaling	2.2E-01	MMP1,PRL
Melanocyte Development and Pigmentation Signaling	2.2E-01	SRC
PAK Signaling	2.2E-01	PTK2
p53 Signaling	2.2E-01	HIF1A
UVA-Induced MAPK Signaling	2.2E-01	PRKCA
Apoptosis Signaling	2.3E-01	PRKCA
PD-1, PD-L1 cancer immunotherapy pathway	2.4E-01	CSK
Telomerase Signaling	2.4E-01	PDPK1
Gαs Signaling	2.4E-01	SRC
Rac Signaling	2.5E-01	PTK2
NGF Signaling	2.6E-01	PDPK1
Endocannabinoid Developing Neuron Pathway	2.6E-01	SRC
RhoA Signaling	2.7E-01	PTK2
Gαi Signaling	2.8E-01	SRC
FXR/RXR Activation	2.8E-01	IL1A
White Adipose Tissue Browning Pathway	2.9E-01	NPPB
Semaphorin Neuronal Repulsive Signaling Pathway	2.9E-01	FYN
Cellular Effects of Sildenafil (Viagra)	2.9E-01	PDE5A
Adipogenesis pathway	3.0E-01	HIF1A
Th2 Pathway	3.0E-01	IL1RL1
Relaxin Signaling	3.2E-01	PDE5A
Gustation Pathway	3.3E-01	PDE5A
PKCθ Signaling in T Lymphocytes	3.3E-01	FYN
Glioblastoma Multiforme Signaling	3.5E-01	SRC
Cdc42 Signaling	3.5E-01	SRC
Mitochondrial Dysfunction	3.6E-01	APP
Th1 and Th2 Activation Pathway	3.6E-01	IL1RL1
Dendritic Cell Maturation	3.8E-01	IL1A
Sertoli Cell-Sertoli Cell Junction Signaling	3.8E-01	SRC
Regulation Of The Epithelial Mesenchymal Transition By Growth Factors Pathway	3.9E-01	MMP1
Regulation of the Epithelial-Mesenchymal Transition Pathway	3.9E-01	HIF1A
Clathrin-mediated Endocytosis Signaling	4.0E-01	SRC
Calcium Signaling	4.2E-01	TPM4
Signaling by Rho Family GTPases	4.7E-01	PTK2
Senescence Pathway	5.1E-01	IL1A
Systemic Lupus Erythematosus In T Cell Signaling Pathway	5.8E-01	PTK2

Table S4. Canonical pathways for metastatic vs control.

Ingenuity Canonical Pathways	*p*-value	Molecules
Iron homeostasis signaling pathway	2.5E-10	BMP1,BMP6,EGF,FTH1,GDF15,HMOX2,PDGFA,PDGFRA
Hepatic Fibrosis / Hepatic Stellate Cell Activation	2.9E-09	CCL5,EGF,FN1,IL1RL1,LEP,PDGFA,PDGFRA,TIMP1
Granulocyte Adhesion and Diapedesis	6.2E-08	CCL22,CCL5,CX3CL1,CXCL13,IL1RL1,MMP10,PPBP
Agranulocyte Adhesion and Diapedesis	9.8E-08	CCL22,CCL5,CX3CL1,CXCL13,FN1,MMP10,PPBP
STAT3 Pathway	2.6E-07	BMP6,EGF,GHR,IL1RL1,MAP2K4,PDGFRA
Hepatic Fibrosis Signaling Pathway	5.6E-07	CCL5,FTH1,IL1RL1,LEP,MAP2K4,PDGFA,PDGFRA,TIMP1
Role of Macrophages, Fibroblasts and Endothelial Cells in Rheumatoid Arthritis	2.5E-06	CCL5,FN1,IL1RL1,MAP2K4,PDGFA,PRSS1,PRSS2
Human Embryonic Stem Cell Pluripotency	6.9E-06	BMP1,BMP6,INHBA,PDGFA,PDGFRA
VDR/RXR Activation	1.7E-05	CCL5,CST6,IL1RL1,PDGFA
Acute Phase Response Signaling	2.7E-05	FN1,HMOX2,MAP2K4,PLG,SERPINA1
Osteoarthritis Pathway	5.9E-05	ADIPOQ,FN1,IL1RL1,LEP,MMP10
Neuroprotective Role of THOP1 in Alzheimer's Disease	8.1E-05	APP,PLG,PRSS1,PRSS2
Axonal Guidance Signaling	3.6E-04	BMP1,BMP6,EGF,MMP10,PDGFA,SEMA6B
Regulation Of The Epithelial Mesenchymal Transition By Growth Factors Pathway	5.1E-04	EGF,MAP2K4,PDGFA,PDGFRA
PPARα/RXRα Activation	5.4E-04	ADIPOQ,GHR,IL1RL1,MAP2K4
BMP signaling pathway	6.5E-04	BMP1,BMP6,MAP2K4
PDGF Signaling	6.6E-04	MAP2K4,PDGFA,PDGFRA
HIF1α Signaling	7.1E-04	BMP6,EGF,MAP2K4,MMP10
Role of Osteoblasts, Osteoclasts and Chondrocytes in Rheumatoid Arthritis	9.3E-04	BMP1,BMP6,IL1RL1,MAP2K4
PAK Signaling	9.3E-04	MAP2K4,PDGFA,PDGFRA
IL-17A Signaling in Gastric Cells	1.1E-03	CCL5,MAP2K4
PPAR Signaling	1.1E-03	IL1RL1,PDGFA,PDGFRA
Glioma Signaling	1.3E-03	EGF,PDGFA,PDGFRA
Coagulation System	2.2E-03	PLG,SERPINA1
Inhibition of Matrix Metalloproteases	2.7E-03	MMP10,TIMP1
Neuroinflammation Signaling Pathway	2.9E-03	APP,CCL5,CX3CL1,MAP2K4
Factors Promoting Cardiogenesis in Vertebrates	3.2E-03	BMP1,BMP6,MAP2K4
Role of Pattern Recognition Receptors in Recognition of Bacteria and Viruses	3.5E-03	CCL5,LEP,MAP2K4
Glioblastoma Multiforme Signaling	4.3E-03	EGF,PDGFA,PDGFRA
PI3K/AKT Signaling	5.0E-03	GDF15,GHR,IL1RL1
Role of IL-17A in Arthritis	5.1E-03	CCL5,MAP2K4
EGF Signaling	5.4E-03	EGF,MAP2K4
NF-κB Signaling	5.4E-03	EGF,GHR,PDGFRA
Hepatic Cholestasis	5.9E-03	IL1RL1,LEP,MAP2K4
Thyroid Hormone Biosynthesis	5.9E-03	CTSD
SPINK1 Pancreatic Cancer Pathway	6.3E-03	PRSS1,PRSS2
Clathrin-mediated Endocytosis Signaling	6.6E-03	EGF,PDGFA,SERPINA1
Leukocyte Extravasation Signaling	6.9E-03	MAP2K4,MMP10,TIMP1
Heme Degradation	7.9E-03	HMOX2
IL-10 Signaling	8.3E-03	IL1RL1,MAP2K4
SPINK1 General Cancer Pathway	8.3E-03	PRSS1,PRSS2
Basal Cell Carcinoma Signaling	8.9E-03	BMP1,BMP6

Actin Cytoskeleton Signaling	9.1E-03	EGF,FN1,PDGFA
Glioma Invasiveness Signaling	9.5E-03	PLG,TIMP1
Toll-like Receptor Signaling	1.0E-02	IL1RL1,MAP2K4
Macropinocytosis Signaling	1.0E-02	EGF,PDGFA
IL-17 Signaling	1.1E-02	MAP2K4,TIMP1
Role of MAPK Signaling in the Pathogenesis of Influenza	1.1E-02	CCL5,MAP2K4
Huntington's Disease Signaling	1.1E-02	CTSD,EGF,MAP2K4
Ceramide Signaling	1.3E-02	CTSD,MAP2K4
Colorectal Cancer Metastasis Signaling	1.4E-02	EGF,MAP2K4,MMP10
CCR5 Signaling in Macrophages	1.5E-02	CCL5,MAP2K4
ErbB Signaling	1.5E-02	EGF,MAP2K4
TGF-β Signaling	1.5E-02	INHBA,MAP2K4
Cardiac Hypertrophy Signaling (Enhanced)	1.5E-02	GHR,IL1RL1,LEP,MAP2K4
Bladder Cancer Signaling	1.6E-02	EGF,MMP10
Pathogenesis of Multiple Sclerosis	1.8E-02	CCL5
Pancreatic Adenocarcinoma Signaling	1.9E-02	EGF,MAP2K4
Role of PKR in Interferon Induction and Antiviral Response	2.2E-02	MAP2K4,PDGFA
Sphingosine-1-phosphate Signaling	2.2E-02	PDGFA,PDGFRA
Renin-Angiotensin Signaling	2.3E-02	CCL5,MAP2K4
p38 MAPK Signaling	2.3E-02	IL1RL1,MAP2K4
Role of NANOG in Mammalian Embryonic Stem Cell Pluripotency	2.3E-02	BMP1,BMP6
LXR/RXR Activation	2.4E-02	IL1RL1,SERPINA1
IL-15 Production	2.4E-02	MAP2K4,PDGFRA
IL-6 Signaling	2.5E-02	IL1RL1,MAP2K4
FXR/RXR Activation	2.6E-02	MAP2K4,SERPINA1
PTEN Signaling	2.6E-02	GHR,PDGFRA
Atherosclerosis Signaling	2.6E-02	PDGFA,SERPINA1
Reelin Signaling in Neurons	2.7E-02	APP,MAP2K4
Estrogen Receptor Signaling	2.7E-02	EGF,LEP,MMP10
Semaphorin Neuronal Repulsive Signaling Pathway	2.8E-02	MAP2K4,SEMA6B
IL-12 Signaling and Production in Macrophages	2.8E-02	MAP2K4,SERPINA1
Type II Diabetes Mellitus Signaling	3.2E-02	ADIPOQ,MAP2K4
Differential Regulation of Cytokine Production in Macrophages and T Helper Cells by IL-17A and IL-17F	3.5E-02	CCL5
Coronavirus Pathogenesis Pathway	3.5E-02	CCL5,MAP2K4
Molecular Mechanisms of Cancer	4.2E-02	BMP1,BMP6,MAP2K4
HMGB1 Signaling	4.3E-02	LEP,MAP2K4
Differential Regulation of Cytokine Production in Intestinal Epithelial Cells by IL-17A and IL-17F	4.5E-02	CCL5
Mitochondrial Dysfunction	4.5E-02	APP,MAP2K4
IL-22 Signaling	4.7E-02	MAP2K4
Tumoricidal Function of Hepatic Natural Killer Cells	4.7E-02	LYVE1
Role of JAK family kinases in IL-6-type Cytokine Signaling	4.8E-02	MAP2K4
Ephrin Receptor Signaling	4.9E-02	EGF,PDGFA
Apelin Liver Signaling Pathway	5.0E-02	MAP2K4
Dendritic Cell Maturation	5.1E-02	LEP,MAP2K4
Production of Nitric Oxide and Reactive Oxygen Species in Macrophages	5.4E-02	MAP2K4,SERPINA1

NRF2-mediated Oxidative Stress Response	5.4E-02	FTH1,MAP2K4
ILK Signaling	5.5E-02	FN1,MAP2K4
Regulation of the Epithelial-Mesenchymal Transition Pathway	5.5E-02	EGF,MAP2K4
TNFR2 Signaling	5.8E-02	MAP2K4
IL-8 Signaling	6.0E-02	EGF,MAP2K4
4-1BB Signaling in T Lymphocytes	6.2E-02	MAP2K4
Inhibition of Angiogenesis by TSP1	6.5E-02	MAP2K4
Role of JAK2 in Hormone-like Cytokine Signaling	6.5E-02	GHR
AMPK Signaling	6.8E-02	ADIPOQ,LEP
Sperm Motility	7.2E-02	MAP2K4,PDGFRA
LPS/IL-1 Mediated Inhibition of RXR Function	7.2E-02	IL1RL1,MAP2K4
Docosahexaenoic Acid (DHA) Signaling	7.2E-02	APP
April Mediated Signaling	7.4E-02	MAP2K4
B Cell Activating Factor Signaling	7.8E-02	MAP2K4
MIF Regulation of Innate Immunity	7.9E-02	MAP2K4
Oncostatin M Signaling	8.1E-02	CHI3L1
Role of Hypercytokinemia/hyperchemokinemia in the Pathogenesis of Influenza	8.1E-02	CCL5
Apelin Pancreas Signaling Pathway	8.3E-02	MAP2K4
PFKFB4 Signaling Pathway	8.7E-02	MAP2K4
TNFR1 Signaling	9.3E-02	MAP2K4
Amyloid Processing	9.3E-02	APP
UVC-Induced MAPK Signaling	9.5E-02	MAP2K4
UVB-Induced MAPK Signaling	9.8E-02	MAP2K4
CD27 Signaling in Lymphocytes	1.0E-01	MAP2K4
Maturity Onset Diabetes of Young (MODY) Signaling	1.1E-01	ADIPOQ
PCP pathway	1.1E-01	MAP2K4
Induction of Apoptosis by HIV1	1.1E-01	MAP2K4
Autophagy	1.1E-01	CTSD
Activation of IRF by Cytosolic Pattern Recognition Receptors	1.2E-01	MAP2K4
Role of PI3K/AKT Signaling in the Pathogenesis of Influenza	1.2E-01	CCL5
IL-17A Signaling in Airway Cells	1.2E-01	MAP2K4
CD40 Signaling	1.2E-01	MAP2K4
Regulation of Cellular Mechanics by Calpain Protease	1.2E-01	EGF
Pyridoxal 5'-phosphate Salvage Pathway	1.2E-01	MAP2K4
Growth Hormone Signaling	1.3E-01	GHR
Melatonin Signaling	1.3E-01	MAP2K4
ERK5 Signaling	1.3E-01	EGF
Caveolar-mediated Endocytosis Signaling	1.3E-01	EGF
Non-Small Cell Lung Cancer Signaling	1.3E-01	EGF
Leptin Signaling in Obesity	1.4E-01	LEP
TREM1 Signaling	1.4E-01	IL1RL1
Fcγ RIIB Signaling in B Lymphocytes	1.4E-01	MAP2K4
GDNF Family Ligand-Receptor Interactions	1.4E-01	MAP2K4
Neurotrophin/TRK Signaling	1.4E-01	MAP2K4
Systemic Lupus Erythematosus In T Cell Signaling Pathway	1.4E-01	LEP,MAP2K4
Glucocorticoid Receptor Signaling	1.4E-01	CCL5,MAP2K4
Agrin Interactions at Neuromuscular Junction	1.4E-01	MAP2K4

BEX2 Signaling Pathway	1.4E-01	MAP2K4
Chemokine Signaling	1.5E-01	CCL5
LPS-stimulated MAPK Signaling	1.5E-01	MAP2K4
HER-2 Signaling in Breast Cancer	1.5E-01	EGF
RANK Signaling in Osteoclasts	1.6E-01	MAP2K4
Acute Myeloid Leukemia Signaling	1.6E-01	MAP2K4
Regulation of IL-2 Expression in Activated and Anergic T Lymphocytes	1.6E-01	MAP2K4
Altered T Cell and B Cell Signaling in Rheumatoid Arthritis	1.6E-01	CXCL13
OX40 Signaling Pathway	1.6E-01	MAP2K4
IL-1 Signaling	1.6E-01	MAP2K4
Death Receptor Signaling	1.6E-01	MAP2K4
FAK Signaling	1.7E-01	EGF
Neuregulin Signaling	1.7E-01	EGF
Communication between Innate and Adaptive Immune Cells	1.7E-01	CCL5
ATM Signaling	1.7E-01	MAP2K4
Salvage Pathways of Pyrimidine Ribonucleotides	1.8E-01	MAP2K4
UVA-Induced MAPK Signaling	1.8E-01	MAP2K4
Apoptosis Signaling	1.8E-01	MAP2K4
SAPK/JNK Signaling	1.8E-01	MAP2K4
Sumoylation Pathway	1.8E-01	MAP2K4
T Cell Receptor Signaling	1.9E-01	MAP2K4
Telomerase Signaling	1.9E-01	EGF
Paxillin Signaling	1.9E-01	MAP2K4
Antioxidant Action of Vitamin C	1.9E-01	MAP2K4
HGF Signaling	2.0E-01	MAP2K4
Type I Diabetes Mellitus Signaling	2.0E-01	MAP2K4
Rac Signaling	2.0E-01	MAP2K4
NGF Signaling	2.0E-01	MAP2K4
Endocannabinoid Developing Neuron Pathway	2.0E-01	MAP2K4
Apelin Endothelial Signaling Pathway	2.0E-01	MAP2K4
Fc Epsilon RI Signaling	2.1E-01	MAP2K4
Airway Pathology in Chronic Obstructive Pulmonary Disease	2.1E-01	LEP
Cholecystokinin/Gastrin-mediated Signaling	2.1E-01	MAP2K4
CD28 Signaling in T Helper Cells	2.1E-01	MAP2K4
Phagosome Formation	2.2E-01	FN1
14-3-3-mediated Signaling	2.2E-01	MAP2K4
White Adipose Tissue Browning Pathway	2.3E-01	LEP
Gα12/13 Signaling	2.3E-01	MAP2K4
Adipogenesis pathway	2.3E-01	LEP
Th2 Pathway	2.4E-01	IL1RL1
Ovarian Cancer Signaling	2.4E-01	EGF
Aryl Hydrocarbon Receptor Signaling	2.5E-01	CTSD
Endocannabinoid Cancer Inhibition Pathway	2.5E-01	MAP2K4
Xenobiotic Metabolism General Signaling Pathway	2.5E-01	MAP2K4
Phagosome Maturation	2.6E-01	CTSD
Epithelial Adherens Junction Signaling	2.6E-01	EGF
PKCθ Signaling in T Lymphocytes	2.6E-01	MAP2K4
HOTAIR Regulatory Pathway	2.7E-01	MMP10
Tec Kinase Signaling	2.8E-01	MAP2K4

CXCR4 Signaling	2.8E-01	MAP2K4
Cdc42 Signaling	2.8E-01	MAP2K4
Germ Cell-Sertoli Cell Junction Signaling	2.9E-01	MAP2K4
Th1 and Th2 Activation Pathway	2.9E-01	IL1RL1
GNRH Signaling	2.9E-01	MAP2K4
T Cell Exhaustion Signaling Pathway	2.9E-01	MAP2K4
Sertoli Cell-Sertoli Cell Junction Signaling	3.1E-01	MAP2K4
B Cell Receptor Signaling	3.1E-01	MAP2K4
Xenobiotic Metabolism CAR Signaling Pathway	3.1E-01	MAP2K4
RAR Activation	3.2E-01	MAP2K4
Adrenomedullin signaling pathway	3.2E-01	MAP2K4
Gap Junction Signaling	3.3E-01	EGF
Breast Cancer Regulation by Stathmin1	3.3E-01	BMP6,EGF
Thrombin Signaling	3.4E-01	EGF
Integrin Signaling	3.5E-01	MAP2K4
Role of NFAT in Cardiac Hypertrophy	3.5E-01	MAP2K4
EIF2 Signaling	3.6E-01	EIF5
Cardiac Hypertrophy Signaling	3.8E-01	MAP2K4
Insulin Secretion Signaling Pathway	3.8E-01	GHR
Signaling by Rho Family GTPases	3.8E-01	MAP2K4
Opioid Signaling Pathway	3.9E-01	MAP2K4
Systemic Lupus Erythematosus In B Cell Signaling Pathway	4.2E-01	LEP
Senescence Pathway	4.2E-01	MAP2K4
Xenobiotic Metabolism Signaling	4.4E-01	MAP2K4
Sirtuin Signaling Pathway	4.4E-01	APP

Table S5. List of differentially expressed proteins between cancer patients with cachexia vs no cachexia.

Protein	Fold change	p-value
C1R	2.393	0.007504
XPNPEP1	2.148	0.022593
IFNAR1	1.979	0.009908
HTRA2	1.819	0.029699
PDGFRA	1.787	0.048975
ENG	1.729	0.012364
GPI	1.675	0.044112
TNFRSF12A	1.633	0.048589
NAMPT	1.616	0.00389
CD163	1.528	0.037976
GREM1	-1.523	0.015568
OCIAD1	-1.525	0.034185
MASP1	-1.528	0.008323
SLAMF7	-1.544	0.048477
TPSB2	-1.547	0.022328
PRKCD	-1.556	0.038211
KLK4	-1.575	0.015193
CDH12	-1.579	0.009702
BIRC5	-1.581	0.027019
FGF17	-1.601	0.035756

CAMK1D	-1.614	0.016701
TNFRSF11A	-1.627	0.019358
PAK7	-1.631	0.0058
BPI	-1.672	0.028089
Human-virus	-1.679	0.001196
EPS15L1	-1.702	0.001735
P4HB	-1.717	0.005856
REG1A	-1.719	0.015209
MSTN	-1.751	0.012037
JAM3	-1.768	0.032831
SMPDL3A	-1.769	0.026528
TNFRSF9	-1.772	0.023748
FGF6	-1.778	0.018905
CSF2	-1.784	0.023655
KIF23	-1.785	0.019227
CXCL1	-1.804	0.048311
CCL26	-1.832	0.007663
ADAMTS1	-1.842	0.006132
PRDX6	-1.857	0.041467
SERPINA6	-1.862	0.042341
LDLR	-1.865	0.036482
BAD	-1.899	0.048084
LTA	-1.944	0.029358
PA2G4	-1.959	0.037797
SOST	-1.981	0.017399
CLC	-2.005	0.008647
LCK	-2.031	0.021689
SPTAN1	-2.032	0.027508
PRKCI	-2.102	0.017282
EEF1B2	-2.122	0.009298
AREG	-2.130	0.049976
RSPO2	-2.173	0.006493
TNFRSF10A	-2.191	0.015063
ELANE	-2.267	0.027178
CSNK2A1 CSNK2B	-2.384	0.022957
CDC37	-2.412	0.024713
TNFRSF19	-2.466	0.043344
KIR2DL4	-2.552	0.018723
IL5	-2.691	0.04448
ACP1	-2.875	0.007607
IRF1	-3.304	0.010506
RAN	-3.351	0.017916
KEAP1	-3.663	0.036911
PDE3A	-3.726	0.003463
PRKCG	-3.825	0.024676
PRSS1	-5.360	0.00034
PRSS2	-5.586	0.000545

Table S6. Predicted upstream regulators of proteins differentially present in patients with cancer cachexia.

Upstream Regulator	Molecule Type	Predicted Activation State	Activation *z*-score	*p*-value of overlap	Target Molecules in Dataset	Mechanistic Network
TGFB1	growth factor		-1.094	1.1E-14	AFM,ALB,BGLAP,CD163,CFD,CFH,COL18A1,CSF1R,F2,FCER2,FCGR3A/FCGR3B,GDNF,IL1RL1,IMPDH1,INHBA,KLK11,KRAS,LEP,LTA,LYVE1,MRC1,MSTN,NANOG,PDXK,PIM1,PLAUR,PRSS1,SERPINA1,SPP1,TLR2,TNFRSF10A,TYMS	52 (17)
TNF	cytokine		-0.551	2.8E-10	ALB,ASGR1,BGLAP,CCL28,CD163,CFD,CSF1R,CXCL5,DLL4,ENTPD5,FCER2,GDNF,GHR,HSPD1,IL1R2,IL1RL1,INHBA,LEP,LYVE1,MSTN,NADPH,PIM1,PLAUR,S100A7,SPP1,TLR2,TNFRSF10A	52 (19)
dexamethasone	chemical drug		0.236	1.5E-09	ALB,BGLAP,CD163,CFD,CSF1R,DLL1,GHR,GRP,HSD17B1,IBSP,IL1R2,IL1RL1,INHBA,LEP,LRP1,LTA,LYVE1,MSTN,NADPH,NTN4,PIM1,PLAUR,RPS7,SERPINA1,SPP1,TLR2,TNFRSF10A,TYMS	55 (14)
IFNG	cytokine		0.537	6.4E-09	CCL28,CD163,CSF1R,CXCL5,DLL1,FCER2,FCGR3A/FCGR3B,GDNF,HSPD1,IL1RL1,INHBA,KRAS,LEP,LTA,MRC1,PIM1,PLAUR,S100A7,SERPINA1,SPP1,TLR2,TNFRSF10A	50 (19)
CSF2	cytokine		-1.344	7.0E-09	CD163,CFH,CSF1R,HSPD1,IL1RL1,INHBA,LEP,LTA,LY9,MRC1,PIM1,PLAUR,SPP1,TLR2	56 (19)
tetradecanoylphorbol acetate	chemical drug		-1.329	7.2E-09	ALB,BGLAP,CD163,CMPK1,CSF1R,FCER2,HSD17B1,IBSP,INHBA,LTA,LYVE1,MRC1,PIM1,PLAUR,SERPINA1,SPP1,TLR2,TNFRSF10A,TNFRSF8,TYMS	53 (15)
KLF4	transcription regulator		-0.931	1.1E-08	ALB,CSF1R,DLL1,DLL4,IBSP,INHBA,LPO,MRC1,NANOG,PLAUR,SERPINA1	
glucocorticoid	chemical drug		-0.18	1.1E-08	BGLAP,CD163,CSF1R,FCER2,IBSP,IL1R2,LEP,LTA,SOST,SPP1,TLR2	43 (14)
beta-estradiol	chemical - endogenous mammalian		0.458	2.0E-08	ALB,BGLAP,CDH15,CLEC1B,CXCL5,DLL1,FABP3,GDNF,GHR,HSD17B1,HSPD1,IL1R2,INHBA,KLK11,KRAS,LEP,LPO,MB,MDK,MRC1,PLAUR,S100A7,SERPINA1,SET,SOST,TLR2	41 (7)
FGF2	growth factor		-0.471	4.0E-08	ALB,BGLAP,DLL1,DLL4,GDNF,IBSP,INHBA,KRAS,LYVE1,PLAUR,SOST,SPP1	33 (13)
IL1B	cytokine		-0.765	8.7E-08	BGLAP,CCL28,CXCL5,DLL4,FCER2,GHR,IBSP,IL1R2,IL1RL1,INHBA,LEP,LTA,NADPH,PIM1,SPP1,TLR2,TNFRSF10A	51 (20)
IL4	cytokine		-0.194	9.3E-08	CD163,COL18A1,CRLF2,CSF1R,FCER2,FCGR3A/FCGR3B,IFNAR1,IL1R2,IL1RL1,IMPDH1,LRP1,LTA,MRC1,PIM1,SPP1,TLR2,TNFRSF8	42 (12)
lipopolysaccharide	chemical drug		0.474	1.5E-07	ALB,CD163,CFD,CRLF2,CSF1R,CXCL5,DLL4,F2,FABP3,FCER2,GHR,IL17B,IL1R2,INHBA,LEP,LRP1,LTA,MB,MRC1,PIM1,PLAUR,SERPINA1,SPP1,TLR2	50 (16)
PRLR	transmembrane receptor		1	1.7E-07	CFD,GDNF,GHR,LEP,PIM1	22 (6)

JUN	transcription regulator		0.423	1.8E-07	BGLAP,CFD,CSF1R,CXCL5,GDNF,IBSP,IL1RL1,NANOG,PIM1,PLAUR,SPP1,TNFRSF10A	53 (15)
SP7	transcription regulator			1.9E-07	BGLAP,IBSP,SOST,SPP1	
APP	other		1.499	3.9E-07	ALB,CFH,COL18A1,CSF1R,CXCL5,FABP3,GDNF,GHR,HSPD1,INHBA,LRP1,MDK,MRC1,SET,TLR2,TNFRSF10A	35 (5)
ENPP1	enzyme			4.2E-07	BGLAP,LEP,SPP1	
NOTCH1	transcription regulator		-1.526	4.7E-07	BGLAP,CFD,CSF1R,DLL1,DLL4,LYVE1,NADPH,SOST,SPP1	35 (9)
TREM1	transmembrane receptor		-0.865	5.2E-07	ABL2,CXCL5,FABP3,IL1RL1,INHBA,LY9,SPP1,TLR2	22 (5)
IL13	cytokine		-0.856	5.3E-07	CD163,COL18A1,CXCL5,FCER2,IL1R2,IL1RL1,KLK11,LILRB2,MRC1,SERPINA1,SPP1	46 (14)
IL1	group		-0.288	6.6E-07	ALB,CSF1R,CXCL5,GDNF,IL1R2,IL1RL1,INHBA,LEP,LRP1,TLR2	49 (15)
EGF	growth factor		0.452	7.4E-07	CSF1R,CXCL5,HSD17B1,IL1R2,INHBA,KRAS,MB,NANOG,PLAUR,S100A7,SERPINA1,SPP1	48 (14)
IL10	cytokine	Inhibited	-2.286	8.2E-07	CD163,CSF1R,CXCL5,FCER2,FCGR3A/FCGR3B,IL1R2,INHBA,LILRB2,MRC1,SPP1,TLR2	46 (15)
IL6	cytokine		-0.235	8.7E-07	ALB,CD163,CFD,CFH,CSF1R,CXCL5,FCER2,IL1RL1,LEP,PIM1,S100A7,SERPINA1,SPP1,TLR2	54 (18)
Immunoglobulin	complex		0.599	1.1E-06	C7,CFD,CSF1R,CXCL5,FCGR3A/FCGR3B,IL1R2,INHBA,LILRB2,LTA,NANOG,SOST,SPP1,TLR2	35 (10)
LIF	cytokine		0.163	1.3E-06	AHSG,BGLAP,CSF1R,IBSP,LEP,NANOG,PIM1,SPP1	38 (15)
JUNB	transcription regulator		-1.103	1.3E-06	CFD,IBSP,IL1RL1,INHBA,PIM1,PLAUR,TNFRSF8	25 (6)
calcitriol	chemical drug		-1.721	1.5E-06	ALB,BGLAP,CFD,CSF1R,FCER2,GHR,HSPD1,LEP,MRC1,SERPINA1,SPP1,TLR2	47 (13)
MYC	transcription regulator		-0.221	1.6E-06	ALB,CSF1R,F2,HSPD1,IFNA7,INHBA,KRAS,LEP,MRC1,NANOG,PLAUR,RPS7,SERPINA1,SPP1,TNFRSF10A,TNFRSF8,TYMS	30 (6)
PDLIM7	other			2.3E-06	BGLAP,IBSP,SPP1	
erlotinib	chemical drug		-0.737	2.4E-06	CXCL5,FCER2,PLAUR,TLR2,TNFRSF10A,TYMS	
IL2	cytokine		0.77	2.9E-06	CSF1R,FCGR3A/FCGR3B,HSPD1,IL1R2,IL1RL1,LTA,PIM1,SPP1,STK17B,TLR2,TNFRSF10A,TNFRSF8	52 (20)
actinomycin D	biologic drug	Activated	2.18	3.0E-06	ALB,IL1R2,LTA,NADPH,PLAUR,SPP1,TLR2,TNFRSF10A	43 (14)
Mir218	microRNA			3.5E-06	IBSP,SOST,SPP1	
CEBPB	transcription regulator		-0.481	3.6E-06	ALB,BGLAP,CFD,CSF1R,CXCL5,HSPD1,LEP,PLAUR,SERPINA1,SPP1	39 (12)
tretinoin	chemical - endogenous mammalian		-0.05	3.6E-06	BGLAP,CSF1R,CXCL5,DLL1,DLL4,GHR,HSD17B1,HSPD1,INHBA,LILRB2,MDK,NANOG,PIM1,PLAUR,RPS7,SEMA6B,SPP1,TLR2,TNFRSF10A	37 (12)
SREBF1	transcription regulator		0	4.2E-06	BGLAP,CFD,FABP3,HSD17B1,IL1R2,LEP,MDK,SERPINA1	13 (3)
IL1A	cytokine		-0.641	4.4E-06	CXCL5,IL1R2,INHBA,LEP,S100A7,SERPINA1,SPP1,TLR2	51 (19)
MSTN	growth factor		0.492	4.9E-06	BGLAP,IBSP,INHBA,LEP,SPP1	37 (15)
SP1	transcription regulator			5.3E-06	BGLAP,CXCL5,ENTPD5,GHR,HSD17B1,MRC1,PIM1,PLAUR,SPP1,TLR2,TYMS	

GDF11	growth factor		0.061	5.4E-06	BGLAP,IBSP,MSTN,SPP1	
PD98059	chemical - kinase inhibitor		-0.831	6.5E-06	BGLAP,CXCL5,GDNF,LEP,MB,PIM1,PLAUR,SERPINA1,SPP1,TLR2,TNFRSF10A	44 (16)
resiquimod	chemical drug		-0.11	8.7E-06	CSF1R,DLL4,FCER2,INHBA,LTA,SPP1,TLR2,TNFRSF8	46 (18)
cycloheximide	chemical reagent		-1.254	9.5E-06	ALB,IBSP,IL1R2,IL1RL1,LEP,PLAUR,SPP1,TNFRSF10A,TYMS	41 (17)
TLR4	transmembrane receptor		-1.091	1.1E-05	CD163,DLL4,GDNF,IFNA7,INHBA,LTA,SPP1,TLR2,TNFRSF10A	48 (17)
PI3K (complex)	complex		0.342	1.5E-05	DLL4,KRAS,LEP,MRC1,NANOG,PIM1,SPP1,TNFRSF10A	38 (9)
sirolimus	chemical drug		-0.426	1.7E-05	BGLAP,CFD,HSPD1,LEP,LILRB2,MRC1,NANOG,PIM1,PLAUR,RPS7,TYMS	30 (5)
RBPJ	transcription regulator		-0.447	1.8E-05	DLL1,DLL4,GDNF,INHBA,MDK,MSTN	
Tnf (family)	group		-0.625	1.8E-05	CD163,GHR,INHBA,MSTN,SOST,TLR2	
resveratrol	chemical drug		-1.526	2.2E-05	BGLAP,CD163,CSF1R,LEP,MRC1,SOST,SPP1,TNFRSF10A	32 (12)
U0126	chemical - kinase inhibitor		-0.429	2.2E-05	BGLAP,CSF1R,CXCL5,GDNF,IL1R2,INHBA,LEP,PLAUR,SPP1,TLR2	54 (19)
TFCP2	transcription regulator			2.3E-05	CFH,SPP1,TYMS	
hydrocortisone	chemical - endogenous mammalian		-0.218	2.4E-05	CD163,CFD,CXCL5,HSPD1,INHBA,TLR2	43 (13)
EGR2	transcription regulator		1	2.4E-05	BGLAP,CSF1R,FCER2,GHR,IL1R2,TNFRSF8	16 (3)
PDGF BB	complex		-0.985	2.7E-05	COL18A1,CSF1R,GDNF,GHR,IL1RL1,INHBA,KRAS,PIM1	46 (8)
Growth hormone	group		0.121	2.9E-05	ALB,BGLAP,COL18A1,GHR,IBSP,MSTN,SPP1	27 (8)
methotrexate	chemical drug		0.943	2.9E-05	ALB,C7,CFH,CSF1R,LRP1,SPP1,TYMS	
ascorbic acid	chemical - endogenous mammalian		0.399	2.9E-05	BGLAP,F2,LPO,NADPH,SPP1	
IL3	cytokine		-0.233	3.3E-05	CSF1R,FCER2,IL1RL1,LTA,LY9,PIM1,RPS7,TLR2	10 (2)
APBB2	other			3.7E-05	LRP1,TYMS	
butyric acid	chemical - endogenous mammalian		0.928	4.1E-05	ALB,C7,HSD17B1,IBSP,IL1RL1,KRAS,LEP,SPP1,TYMS	21 (4)
NOG	growth factor			4.3E-05	BGLAP,IBSP,SOST,SPP1	7 (3)
streptozocin	chemical drug	Inhibited	-2.041	4.8E-05	ALB,BGLAP,DLL4,FABP3,HSPD1,LEP,SPP1	42 (14)
SB203580	chemical - kinase inhibitor		0.388	5.0E-05	BGLAP,CXCL5,FABP3,FCER2,MSTN,PLAUR,SPP1,STK17B,TLR2	32 (11)
CSF1	cytokine	Inhibited	-2.387	5.1E-05	CD163,CSF1R,FCGR3A/FCGR3B,IL1RL1,MRC1,SPP1,TLR2	46 (16)
CD36	transmembrane receptor		0.447	5.2E-05	CXCL5,LEP,LRP1,PLAUR,TNFRSF10A	
Fgfr	group			5.4E-05	GDNF,NANOG,SPP1	
HOXA11	transcription regulator			5.4E-05	BGLAP,GDNF,SPP1	
CSF3	cytokine		-0.059	5.4E-05	CXCL5,GDNF,HSPD1,LTA,PIM1,TLR2	38 (9)
WWTR1	transcription regulator		1.067	5.6E-05	BGLAP,NANOG,SPP1,TYMS	

HES1	transcription regulator	-0.422	6.4E-05	CFD,DLL1,IL1RL1,SPP1	
STAT3	transcription regulator	0.396	7.2E-05	AHSG,BOC,DLL1,LEP,LILRB2,LTA,PIM1,PLAUR,S100A7,SERPINA1	35 (7)
parbendazole	chemical reagent		7.3E-05	IBSP,SPP1	12 (6)
durapatite	chemical - endogenous mammalian		7.3E-05	IBSP,SPP1	
DP-001	chemical drug		7.3E-05	BGLAP,SPP1	6 (2)
MGP	other		8.1E-05	ALB,CFD,SPP1	
IGF1	growth factor	-0.719	8.1E-05	BGLAP,GDNF,GHR,LEP,MSTN,PDXK,PIM1,PLAUR,SPP1	41 (13)
RUNX2	transcription regulator	0.573	8.7E-05	BGLAP,IBSP,LEP,SOST,SPP1	15 (4)
LEP	growth factor	0.594	9.0E-05	BGLAP,CFD,DLL4,FABP3,GDNF,IBSP,IL1R2,LEP,SPP1	41 (16)
PPARG	ligand-dependent nuclear receptor	-1.764	9.1E-05	BGLAP,CFD,CXCL5,FABP3,KLK11,LEP,MRC1,SERPINA1,SPP1	33 (13)
FST	other	-1.98	9.3E-05	FABP3,MSTN,NANOG,SERPINA1	
GHR	transmembrane receptor	0.371	9.3E-05	GHR,LEP,SOST,SPP1	26 (7)
bleomycin	chemical drug	-0.31	1.0E-04	F2,MRC1,SPP1,STK17B,TLR2,TNFRSF10A	9 (2)
GLI1	transcription regulator	0.733	1.0E-04	IBSP,IL1R2,NANOG,PIM1,S100A7,SPP1	
telmisartan	chemical drug		1.0E-04	CD163,GDNF,LEP,MRC1	
phytohemagglutinin	chemical drug	1.088	1.1E-04	AHSG,AKT2,COL18A1,IL1R2,LTA,SPP1,TYMS	
FGFR2	kinase	-0.254	1.1E-04	BGLAP,DLL1,NANOG,PLAUR,SPP1	14 (3)
MET	kinase	-0.266	1.2E-04	INHBA,LTA,NANOG,PIM1,SPP1	20 (3)
elocalcitol	chemical drug		1.2E-04	BGLAP,SPP1	
2,3-bis(3'-hydroxybenzyl)butane-1,4-diol	chemical - endogenous mammalian		1.2E-04	BGLAP,SPP1	
poly rI:rC-RNA	biologic drug	-1.172	1.3E-04	DLL1,DLL4,HSPD1,INHBA,LTA,NANOG,PIM1,PLAUR,TLR2,TNFRSF10A	47 (17)
LY294002	chemical - kinase inhibitor	-1.673	1.3E-04	CXCL5,IL1R2,KRAS,LEP,NANOG,PIM1,SPP1,TLR2,TYMS	43 (17)
NMU	other		1.4E-04	IL1R2,LEP,TNFRSF8	
calcimycin	chemical reagent	-0.164	1.5E-04	HSD17B1,IL1RL1,INHBA,LTA,TLR2	32 (8)
HRAS	enzyme	-0.401	1.6E-04	CFH,COL18A1,DLL1,FABP3,IL1RL1,KRAS,PLAUR,SPP1,TNFRSF10A,TYMS	35 (10)
WNT11	other		1.6E-04	GDNF,IBSP,NANOG	
OSM	cytokine	-0.508	1.8E-04	ALB,CXCL5,IL1R2,MRC1,S100A7,SERPINA1,SOST,SPP1,TLR2	26 (6)
IgG	complex	-1.016	1.8E-04	AFM,INHBA,KLK11,LY9,PIM1,PLAUR	
Tcf 1/3/4	group		1.8E-04	AHSG,ALB	
Ige	complex	-0.478	1.9E-04	COL18A1,HSPD1,KRAS,LTA,SPP1,TLR2,TNFRSF10A	25 (5)
UTP	chemical - endogenous mammalian		1.9E-04	LTA,MB,SPP1	4 (2)

NR3C1	ligand-dependent nuclear receptor		2.0E-04	AKT2,BGLAP,INHBA,LEP,SERPINA1,SPP1,STK17B,TLR2,TNFRSF10A,TNFRSF8	10 (2)
POU5F1	transcription regulator	-0.214	2.0E-04	ALB,INHBA,LTA,NANOG,SERPINA1,SPP1,TNFRSF10A	20 (3)
FOS	transcription regulator	-1.276	2.1E-04	BGLAP,CFD,COL18A1,IBSP,IL1RL1,PIM1,PLAUR,RPS7,SPP1	25 (6)
JUND	transcription regulator		2.1E-04	BGLAP,CFD,PLAUR,SPP1	25 (6)
VDR	transcription regulator	-0.556	2.3E-04	AKT2,BGLAP,LEP,SERPINA1,SPP1,TLR2	29 (12)
PD 0325901	chemical drug		2.3E-04	MRC1,NANOG,TNFRSF10A	
PTH1R	G-protein coupled receptor		2.3E-04	CSF1R,SOST,SPP1	
SFTPA1	transporter	-0.152	2.3E-04	CLEC1B,CXCL5,IL1RL1,MRC1	
IFNAR1	transmembrane receptor		2.4E-04	DLL1,F2,IFNAR1,KLK11,TLR2	
IL17F	cytokine		2.6E-04	CXCL5,LEP,LTA	26 (7)
SU6656	chemical toxicant		2.6E-04	GDNF,IL1RL1,INHBA	
RIOX1	enzyme		2.6E-04	BGLAP,IBSP	4 (2)
ITGB1BP1	other		2.6E-04	DLL1,DLL4	
polyphosphate	chemical - endogenous mammalian		2.6E-04	BGLAP,SPP1	
MED1	transcription regulator		2.6E-04	CFD,GHR,MB,NANOG,PIM1	
D-glucose	chemical - endogenous mammalian	-1.291	2.9E-04	CSF1R,DLL4,HSPD1,INHBA,LEP,LRP1,NADPH,PLAUR,SPP1,TLR2	46 (16)
miR-155-5p (miRNAs w/seed UAAUGCU)	mature microRNA	-0.447	3.0E-04	CSF1R,F2,FCER2,KRAS,TNFRSF10A	
MSX2	transcription regulator		3.0E-04	BGLAP,IBSP,SPP1	15 (5)
FGF19	growth factor		3.2E-04	FABP3,HSD17B1,LEP,SERPINA1	
HMGB1	transcription regulator	-0.339	3.2E-04	CD163,CXCL5,MRC1,TLR2	46 (13)
ACVRL1	kinase		3.3E-04	IL1RL1,LYVE1,PLAUR	
forskolin	chemical toxicant	-0.403	3.3E-04	BGLAP,CD163,COL18A1,CXCL5,HSD17B1,IBSP,INHBA,LEP,PDXK,SPP1	36 (15)
pyrophosphate	chemical - endogenous mammalian		3.4E-04	BGLAP,SPP1	
GAS7	transcription regulator		3.4E-04	BGLAP,SPP1	
OXTR	G-protein coupled receptor		3.4E-04	BGLAP,IBSP	
TCF3	transcription regulator	-1.633	3.5E-04	CSF1R,IFNAR1,IL1RL1,NANOG,STK17B,TNFRSF8,TYMS	7 (2)
FGFR1	kinase	1	3.6E-04	BGLAP,DLL1,PLAUR,PRSS27	23 (6)
HTT	transcription regulator	-0.594	3.6E-04	AKT2,ASGR1,COL18A1,CSF1R,DLL1,HSPD1,PDXK,PLAUR,SERPINA1,SPP1	26 (7)
fluticasone propionate	chemical drug	0.208	3.7E-04	CD163,CXCL5,IL1R2,SERPINA1,SPP1	
FGF8	growth factor	-1.091	3.7E-04	BGLAP,COL18A1,MDK,SPP1	

SMARCA4	transcription regulator	-1.003	3.9E-04	ALB,BGLAP,DLL1,INHBA,NANOG,PLAUR,SPP1,TLR2,TYMS	34 (8)
TP53	transcription regulator	-0.909	4.1E-04	ALB,BGLAP,COL18A1,CSF1R,FABP3,HSPD1,INHBA,MB,MRC1,NADPH,NANOG,PIM1,PLAUR,SPP1,TNFRSF10A,TYMS,XPNPEP1	36 (12)
1,25-dihydroxyvitamin D	chemical drug		4.1E-04	LEP,MSTN,TLR2	8 (3)
POSTN	other		4.1E-04	BGLAP,SOST,SPP1	
laminaran	chemical drug		4.1E-04	CRLF2,CXCL5,INHBA	
P38 MAPK	group	0.728	4.1E-04	FCER2,IL1RL1,INHBA,LEP,PLAUR,SPP1,TLR2	58 (20)
E2F3	transcription regulator	0.447	4.3E-04	COL18A1,HSPD1,INHBA,KRAS,LEP	
(-)-epicatechin gallate	chemical - endogenous non-mammalian		4.4E-04	BGLAP,SPP1	16 (6)
glimepiride	chemical drug		4.4E-04	BGLAP,LEP	
IL17A	cytokine	-0.113	4.4E-04	CD163,CXCL5,LEP,MRC1,S100A7,TLR2	43 (13)
MYD88	other	-1.772	4.4E-04	DLL4,IFNA7,INHBA,MRC1,SPP1,TLR2	27 (7)
CSF1R	kinase		4.4E-04	CD163,CSF1R,MRC1	8 (3)
CEACAM1	transporter		4.4E-04	COL18A1,DLL1,LEP	
ADORA2B	G-protein coupled receptor		4.7E-04	MRC1,NANOG,SPP1	
NFkB (complex)	complex	0.895	4.7E-04	COL18A1,CXCL5,FCER2,GDNF,IFNA7,LTA,MSTN,SPP1,TLR2	23 (6)
SOD1	enzyme		4.9E-04	BGLAP,CSF1R,GDNF,INHBA,SOST,SPP1	29 (8)
IHH	enzyme		5.0E-04	CD163,MRC1,SPP1	
PTGER2	G-protein coupled receptor	1.091	5.0E-04	CSF1R,IL1R2,PIM1,SPP1	18 (3)
puromycin aminonucleoside	chemical reagent	0.152	5.0E-04	ALB,HSPD1,MRC1,SPP1	
TCF12	transcription regulator	-1	5.2E-04	CSF1R,IL1RL1,STK17B,TNFRSF8	7 (2)
IRF4	transcription regulator		5.3E-04	FCER2,PLAUR,SPP1,STK17B,TNFRSF8	
SMAD3	transcription regulator		5.4E-04	ALB,BGLAP,IBSP,MSTN,NANOG,SPP1	
L-threonine	chemical - endogenous mammalian		5.4E-04	ALB,NANOG	
PARP14	enzyme		5.4E-04	FCER2,PIM1	
LDB1	transcription regulator	-0.816	5.5E-04	ABL2,COL18A1,MDK,TLR2,TNFRSF10A,TNFRSF8	
LMO2	transcription regulator	-0.816	5.6E-04	ABL2,COL18A1,MDK,TLR2,TNFRSF10A,TNFRSF8	
VitaminD3-VDR-RXR	complex		5.7E-04	BGLAP,IL1RL1,SPP1	
estrogen	chemical drug	0	5.9E-04	ALB,COL18A1,IL1RL1,PIM1,SPP1,TYMS	43 (12)
GSK2816126	chemical drug		6.1E-04	BGLAP,IBSP,SPP1	
ESR2	ligand-dependent	-1.091	6.2E-04	CLEC1B,IBSP,LEP,MDK,PLAUR,S100A7,SOST,SPP1	22 (5)

	nuclear receptor				
uranyl nitrate	chemical toxicant	-1.067	6.2E-04	ENTPD5,GHR,HSPD1,SPP1	
bexarotene	chemical drug	1.342	6.4E-04	AKT2,CFD,GDNF,GRP,PLAUR	
CDKN2A	transcription regulator	0.557	6.4E-04	IL17B,IL1R2,KRAS,LTA,MRC1,NANOG	
KDR	kinase		6.5E-04	DLL4,PIM1,SET	
Pam3-Cys	chemical toxicant		6.5E-04	CD163,IL1RL1,TLR2	24 (6)
necrostatin-1	chemical reagent		6.6E-04	CD163,MRC1	
NR1D2	ligand-dependent nuclear receptor		6.6E-04	FABP3,MSTN	
enterolactone	chemical - endogenous mammalian		6.6E-04	BGLAP,SPP1	
tiron	chemical reagent		6.6E-04	KRAS,PLAUR	
GATA2	transcription regulator	-1.408	6.7E-04	CSF1R,DLL1,HSD17B1,IL1RL1,MDK,PRSS1,SERPINA1	
MAP2K1	kinase	0.762	6.8E-04	LEP,PIM1,PLAUR,SERPINA1,TNFRSF10A	53 (13)
ionomycin	chemical reagent	-0.398	7.3E-04	FCER2,LTA,PIM1,SPP1,TLR2	47 (16)
Hsp27	group		7.3E-04	BGLAP,CD163,CSF1R	
OGA	enzyme	0.378	7.7E-04	CDH15,CSF1R,FABP3,HSD17B1,IL1RL1,LEP,NANOG	
hydrogen peroxide	chemical - endogenous mammalian	-0.438	7.8E-04	BGLAP,CFD,KRAS,MB,MDK,PIM1,PLAUR,SPP1	37 (14)
diphtheria toxin	chemical - endogenous non-mammalian		7.9E-04	CD163,CFH,IL1R2,TLR2	
ETV6	transcription regulator		7.9E-04	CSF1R,DLL4	
FOXD3	transcription regulator		7.9E-04	AKT2,NANOG	
PROC	peptidase		8.2E-04	CXCL5,F2,TLR2	22 (6)
FHL2	transcription regulator		8.2E-04	BGLAP,IBSP,SPP1	
IL10RA	transmembrane receptor	1.633	8.3E-04	BOC,ENTPD5,GHR,INHBA,SLAMF6,TLR2	
GW501516	chemical drug		8.6E-04	CFD,FABP3,KLK11,NANOG	
ETV6-RUNX1	fusion gene/product	0	9.0E-04	DLL1,IFNAR1,PIM1,SPP1,STK17B,TNFRSF10A	
SB-431542	chemical reagent	-1.103	9.2E-04	IBSP,IL1R2,NANOG,SPP1	7 (2)
TSC22D3	transcription regulator		9.2E-04	BGLAP,LEP,TLR2	
SQ 22536	chemical reagent		9.3E-04	BGLAP,IBSP	
filgrastim	biologic drug	0.342	9.5E-04	CD163,CSF1R,GRP,IL1R2,PLAUR,SPP1,TNFRSF10A	
baicalin	chemical - endogenous non-mammalian		9.7E-04	BGLAP,MDK,TLR2	

PTPRJ	phosphatase		9.7E-04	INHBA,LTA,PLAUR	
FOSL2	transcription regulator		9.7E-04	BGLAP,LEP,SPP1	26 (8)
Ifn	group		9.8E-04	CFH,IFNAR1,SPP1,TLR2	
interferon beta-1a	biologic drug		9.8E-04	CSF1R,PLAUR,RPS7,SERPINA1	
CD44	other	0	9.9E-04	COL18A1,IL1R2,NANOG,SERPINA1,SPP1	
SP3	transcription regulator		1.0E-03	BGLAP,GHR,HSD17B1,PLAUR,TLR2	13 (3)
IL15	cytokine	0.404	1.0E-03	AKT2,FCGR3A/FCGR3B,LEP,LTA,PIM1,SET,TLR2	19 (4)
BMP2	growth factor	-0.054	1.0E-03	ASGR1,BGLAP,IBSP,SOST,SPP1	23 (11)
SRC	kinase		1.0E-03	IBSP,NANOG,PLAUR,SPP1	34 (12)
FN1	enzyme	-0.391	1.1E-03	CXCL5,NANOG,PLAUR,SPP1,TLR2	35 (13)
STAT5a/b	group		1.1E-03	CLEC1B,PIM1,STK17B,TLR2	
RGS10	enzyme		1.1E-03	IL17B,IL1R2,LTA	
anisomycin	chemical - endogenous non-mammalian		1.1E-03	IL1RL1,PLAUR,SPP1	36 (15)
LPAR1	G-protein coupled receptor		1.1E-03	AKT2,GDNF	
CXCL1	cytokine		1.1E-03	KRAS,LEP	
CCND1	transcription regulator	0.218	1.2E-03	C7,LCORL,NANOG,PRSS1,SPP1,TYMS	
FGF1	growth factor	-0.343	1.2E-03	ALB,DLL1,GDNF,SPP1	18 (4)
eicosapentenoic acid	chemical drug	-0.914	1.2E-03	CSF1R,IL1R2,LEP,MRC1	5 (2)
Ins1	other	0.092	1.2E-03	ALB,CFD,COL18A1,FABP3,LEP,LRP1	27 (5)
amphotericin B	chemical drug		1.3E-03	GDNF,SPP1	
cetuximab	biologic drug		1.3E-03	PLAUR,TNFRSF10A	
FNDC5	other		1.3E-03	SOST,SPP1	
AVPR1A	G-protein coupled receptor		1.3E-03	BGLAP,IBSP	
N-ethyl-N-nitrosourea	chemical toxicant		1.3E-03	SERPINA1,SPP1	
WNT1	cytokine		1.3E-03	DLL1,DLL4,NANOG,SPP1	17 (3)
SCARB1	transporter		1.3E-03	FABP3,LTA,SPP1	
FOXC2	transcription regulator		1.3E-03	CFD,DLL1,LEP	
Ccl2	cytokine		1.3E-03	CFD,LEP,NANOG	
mir-27	microRNA		1.4E-03	DLL4,KRAS,MSTN	
CCL11	cytokine		1.4E-03	CCL28,CXCL5,LEP	
GC-GCR dimer	complex		1.4E-03	CD163,IL1R2	
ALK	kinase		1.4E-03	NANOG,TNFRSF8	
EFNB2	kinase		1.4E-03	BGLAP,SOST	
pimozide	chemical drug		1.4E-03	CSF1R,PIM1	
OSMR	transmembrane receptor		1.4E-03	ALB,PIM1,SERPINA1	
BCL3	transcription regulator		1.4E-03	FABP3,PLAUR,S100A7	
emodin	chemical drug		1.4E-03	CSF1R,LEP,MRC1	
WT1	transcription regulator		1.5E-03	CMPK1,CSF1R,KRAS,LRP1,PLAUR	

SOX2	transcription regulator	0.67	1.5E-03	ALB,BGLAP,CSF1R,DLL1,INHBA,NANOG,SERPINA1	31 (6)
L-methionine	chemical - endogenous mammalian		1.5E-03	ALB,SPP1,TYMS	26 (4)
IL25	cytokine		1.5E-03	IL1R2,IL1RL1,TNFRSF8	25 (5)
cyclosporin A	biologic drug	-0.692	1.6E-03	CD163,FCER2,HSPD1,LTA,LYVE1,MRC1,SPP1	42 (10)
L-triiodothyronine	chemical - endogenous mammalian	1.709	1.6E-03	AHSG,AKT2,ALB,BGLAP,F2,LEP,LRP1	19 (5)
CASR	G-protein coupled receptor	-1	1.6E-03	BGLAP,SERPINA1,TLR2,TYMS	
IDH1	enzyme		1.6E-03	CFD,LEP,NADPH	
NEUROG1	transcription regulator		1.6E-03	CFH,FABP3,INHBA	
SNAI2	transcription regulator		1.6E-03	BGLAP,IBSP,NANOG	15 (5)
IL6ST	transmembrane receptor		1.6E-03	ALB,CSF1R,PIM1	24 (7)
PLN	transporter		1.6E-03	AHSG,DLL1,FABP3	
vitamin D	chemical drug		1.6E-03	BGLAP,SPP1,TYMS	20 (8)
HSD11B2	enzyme		1.6E-03	BGLAP,IBSP	
HELLS	enzyme		1.6E-03	HSPD1,KRAS	
carbon monoxide	chemical - endogenous mammalian		1.7E-03	GDNF,LEP,PLAUR	
melatonin	chemical - endogenous mammalian	-1.109	1.7E-03	BGLAP,IBSP,LEP,SPP1	25 (9)
EGLN1	enzyme		1.8E-03	DLL1,INHBA,LEP	
F3	transmembrane receptor		1.8E-03	F2,KRAS,MDK	
TNFSF13B	cytokine		1.8E-03	FCER2,LEP,SPP1	27 (8)
IL17R	complex		1.8E-03	CXCL5,S100A7	
PAEP	other		1.8E-03	CXCL5,IL1RL1	
rosiglitazone	chemical drug	-0.902	1.9E-03	AHSG,CFD,FABP3,INHBA,LEP,LRP1,SOST	
CTNNB1	transcription regulator	-1.324	2.0E-03	BGLAP,CFD,DLL4,GDNF,GHR,NANOG,PLAUR,RPS7,SERPINA1,SPP1	30 (8)
APOE	transporter	0.975	2.0E-03	COL18A1,HSPD1,LEP,LRP1,SEMA6B,SPP1	11 (3)
prednisolone	chemical drug	-1.342	2.0E-03	CD163,DLL1,LPO,MB,SPP1	
ERBB2	kinase	-0.728	2.0E-03	AKT2,COL18A1,CSF1R,DLL1,GHR,IL17B,MRC1,PLAUR,STK17B,TYMS	
HNF1A	transcription regulator	0.068	2.0E-03	AHSG,ALB,ASGR1,CCL28,F2,IFNAR1,SERPINA1	
TMSB4	group		2.0E-03	BGLAP,SPP1	
CREB3L1	transcription regulator		2.0E-03	BGLAP,SPP1	
cystamine	chemical drug		2.0E-03	BGLAP,SPP1	
helenalin	chemical - endogenous non-mammalian		2.0E-03	CXCL5,TNFRSF10A	6 (2)
BSCL2	other		2.0E-03	CFD,IL1RL1,LEP	
DDX58	enzyme		2.0E-03	DLL1,RPS7,SPP1	
CNR1	G-protein coupled receptor	1	2.1E-03	DLL1,GDNF,LEP,MDK	

RET	kinase	-1	2.1E-03	GDNF,GRP,HSPD1,PLAUR	
HGF	growth factor	-1.501	2.2E-03	INHBA,LTA,LYVE1,NANOG,PIM1,PLAUR,SPP1	35 (12)
HRG	other		2.2E-03	FCGR3A/FCGR3B,SPP1	
RNF31	enzyme		2.3E-03	IL17B,IL1R2,IL1RL1	
CISH	other		2.3E-03	LTA,PLAUR,SPP1	
ADAM12	peptidase		2.4E-03	BGLAP,CFD,IL1RL1	
glutathione	chemical - endogenous mammalian		2.4E-03	ALB,TNFRSF10A,TNFRSF8	
docosahexaenoic acid	chemical drug	0.686	2.4E-03	GDNF,LEP,MRC1,MSTN,NADPH	
TCR	complex	0.246	2.4E-03	HSPD1,IFNAR1,IL1RL1,LTA,PIM1,SPP1	49 (14)
testosterone	chemical - endogenous mammalian	-0.816	2.4E-03	HSD17B1,HSPD1,INHBA,KRAS,LEP,MSTN	
miR-31-5p (and other miRNAs w/seed GGCAAGA)	mature microRNA		2.5E-03	CXCL5,SOST	
RNASE1	enzyme		2.5E-03	CXCL5,FCER2	
N-acetyl-L-cysteine	chemical drug	1.264	2.5E-03	MRC1,PLAUR,SPP1,TNFRSF10A,TNFRSF8	30 (7)
Il3	cytokine		2.6E-03	CSF1R,PIM1,SPP1	40 (12)
FAS	transmembrane receptor		2.6E-03	AKT2,COL18A1,LILRB2,SPP1,STK17B,TLR2	
curcumin	chemical drug	0.218	2.6E-03	ENTPD5,HDAC8,LEP,PIM1,TLR2,TNFRSF10A	
Fibrinogen	complex		2.7E-03	FCGR3A/FCGR3B,TLR2	
FABP5	transporter		2.7E-03	FABP3,LEP	
ADM2	other		2.7E-03	LEP,MRC1	
PLX5622	chemical drug		2.8E-03	IL17B,LTA,SPP1	
TNFSF11	cytokine	0	2.8E-03	CSF1R,PIM1,PLAUR,SPP1,TLR2	46 (17)
indomethacin	chemical drug	-0.686	2.8E-03	IL1RL1,MRC1,PIM1,PLAUR,SPP1	42 (13)
lovastatin	chemical drug		2.8E-03	ALB,GDNF,LEP,TNFRSF8	
nitrofurantoin	chemical drug	-0.152	2.8E-03	ALB,SERPINA1,SPP1,TYMS	
Hdac	group	1.273	2.9E-03	CSF1R,DLL1,GDNF,SPP1	
EZH2	transcription regulator	-0.555	2.9E-03	ABL2,BGLAP,IBSP,KRAS,SERPINA1,SOST	
HTATIP2	transcription regulator		3.0E-03	NANOG,SPP1	
CD200	other		3.0E-03	MRC1,TLR2	
COPS5	transcription regulator		3.0E-03	IFNAR1,NANOG	
beta-glycerophosphoric acid	chemical - endogenous mammalian		3.0E-03	BGLAP,SPP1	15 (5)
methylprednisolone	chemical drug	-1.353	3.0E-03	GHR,KLK11,KRAS,LYVE1,PLAUR,SERPINA1,TLR2	10 (2)
Gsk3	group		3.1E-03	IBSP,NANOG,SPP1	27 (8)
MMP9	peptidase		3.1E-03	COL18A1,MSTN,SERPINA1	
CD40LG	cytokine		3.1E-03	CXCL5,FCER2,INHBA,LTA,PIM1,PLAUR	49 (21)
SPI1	transcription regulator	-1.408	3.2E-03	CSF1R,IL1R2,MRC1,SPP1,TLR2	26 (5)
THRB	ligand-dependent		3.2E-03	AKT2,CFD,SPP1,TLR2,TNFRSF10A	

	nuclear receptor					
THZ1	chemical - kinase inhibitor			3.2E-03	STK17B,TNFRSF8	
Ro41-5253	chemical reagent			3.2E-03	CSF1R,HSD17B1	
SB 290157	chemical reagent			3.2E-03	LEP,SPP1	18 (5)
cardiotoxin	chemical - other	Inhibited	-2	3.4E-03	CSF1R,MRC1,SPP1,TNFRSF8	
FFAR3	G-protein coupled receptor			3.4E-03	CA10,CCL28,LEP	
TGFA	growth factor			3.4E-03	ALB,HSD17B1,SERPINA1	3 (2)
PTPN11	phosphatase			3.4E-03	CSF1R,PIM1,PLAUR	24 (6)
STAT5B	transcriptio n regulator		1.109	3.5E-03	LEP,LTA,MB,PIM1,TLR2	
L-tryptophan	chemical - endogenous mammalian			3.5E-03	ALB,LILRB2	
calcipotriene	chemical drug			3.5E-03	BGLAP,SPP1	10 (3)
UDP-N-acetylglucosam ine	chemical - endogenous mammalian			3.5E-03	LEP	
epi-androsterone	chemical - endogenous mammalian			3.5E-03	NADPH	
2-phosphoglycer ic acid	chemical - endogenous mammalian			3.5E-03	NADPH	
TD114-2	chemical - kinase inhibitor			3.5E-03	NANOG	
Ces1b/Ces1c	enzyme			3.5E-03	GHR	
MARCHF7	other			3.5E-03	NANOG	
MARCHF4	enzyme			3.5E-03	TNFRSF10A	
MDH1	enzyme			3.5E-03	NADPH	
HDLBP	transporter			3.5E-03	CSF1R	
ME2	enzyme			3.5E-03	NADPH	
MKKS	other			3.5E-03	LEP	
NXN	enzyme			3.5E-03	NADPH	
PNRC2	other			3.5E-03	LEP	
MARCHF9	other			3.5E-03	TNFRSF10A	
Protamine	group			3.5E-03	IBSP	
binimetinib	chemical drug			3.5E-03	TNFRSF10A	
APBB3	other			3.5E-03	TYMS	
GRIA3	ion channel			3.5E-03	LEP	
UBA2	enzyme			3.5E-03	ALB	
miR-18a-3p (and other miRNAs w/seed CUGCCCU)	mature microRNA			3.5E-03	KRAS	
miR-331-5p (and other miRNAs w/seed UAGGUAU)	mature microRNA			3.5E-03	PLAUR	
miR-105-5p (and other	mature microRNA			3.5E-03	TLR2	

miRNAs w/seed CAAAUGC)				
STAT3-Brachyury	complex	3.5E-03	NANOG	
SS1(dsfv)PE38	biologic drug	3.5E-03	ALB	
LINC00853	other	3.5E-03	CMPK1	
SAA2	other	3.5E-03	BGLAP	
ME1	enzyme	3.5E-03	NADPH	
CDK3	kinase	3.5E-03	TYMS	
Slc25a12	transporter	3.5E-03	NADPH	
ACSS1	enzyme	3.5E-03	LEP	
CYFIP2	other	3.5E-03	KRAS	
astaxanthin	chemical drug	3.5E-03	LEP	
Scgb1b27 (includes others)	other	3.5E-03	F2	
IFITM3	other	3.5E-03	SPP1	
ESX1	transcription regulator	3.5E-03	KRAS	
beta adrenergic receptor agonist	chemical drug	3.5E-03	LEP	
tetrakis-(diisopropyl-guanidine) phthalocyanine	chemical reagent	3.5E-03	KRAS	
zinc tetrakis-(diisopropyl-guanidine) phthalocyanine	chemical reagent	3.5E-03	KRAS	
dexamethasone/tobramycin	chemical drug	3.5E-03	TLR2	
nolatrexed	chemical drug	3.5E-03	TYMS	
PD173955	chemical - kinase inhibitor	3.5E-03	PLAUR	
RKQ peptide	chemical reagent	3.5E-03	TNFRSF8	
TAC peptide	chemical reagent	3.5E-03	TNFRSF8	
TDC	chemical reagent	3.5E-03	TNFRSF8	
FUS-ERG	fusion gene/product	3.5E-03	PIM1	
EWSR1-ERG	fusion gene/product	3.5E-03	PIM1	
AM966	chemical reagent	3.5E-03	GDNF	
5-fluorouridine	chemical - endogenous mammalian	3.5E-03	TYMS	
trifluridine	chemical drug	3.5E-03	TYMS	
2-aminoisobutyric acid	chemical - endogenous mammalian	3.5E-03	ALB	

D-glucono-1,5-lactone 6-phosphate	chemical - endogenous mammalian		3.5E-03	NADPH	
troglitazone	chemical drug	-0.891	3.6E-03	CFD,CXCL5,ENTPD5,FABP3,LEP,SPP1	40 (12)
TP73	transcription regulator	-1.513	3.6E-03	COL18A1,MDK,NADPH,SERPINA1,SPP1,TNFRSF10A	
BMP7	growth factor		3.6E-03	BGLAP,IBSP,SOST,SPP1	7 (2)
MAPK8	kinase		3.7E-03	FCER2,LEP,PLAUR,TLR2	43 (14)
cobalt chloride	chemical reagent		3.7E-03	COL18A1,LEP,PLAUR	
2-deoxyglucose	chemical drug		3.7E-03	CSF1R,LEP,SPP1	35 (9)
TAF4B	transcription regulator		3.8E-03	INHBA,SPP1	
Gm21596/Hmgb1	transcription regulator		3.8E-03	F2,IL1RL1	
HMGB2	transcription regulator		3.8E-03	CXCL5,INHBA	
alpha-tocopherol	chemical drug		3.8E-03	ALB,LEP	
gefitinib	chemical drug		3.8E-03	GHR,IL1R2,PLAUR,TLR2	
IL5	cytokine	0.928	3.8E-03	IL1R2,IL1RL1,LTA,PIM1,TNFRSF8	
LDL	complex		3.9E-03	DLL4,IBSP,LRP1,LTA,TLR2	31 (9)
IGF2	growth factor		3.9E-03	GHR,LEP,PIM1,SPP1	26 (7)
SCAP	other		4.0E-03	ALB,CSF1R,HSD17B1	
E. coli B4 lipopolysaccharide	chemical toxicant	-0.678	4.0E-03	MRC1,PIM1,PLAUR,TLR2,TNFRSF8	25 (4)
TNFRSF11B	transmembrane receptor		4.1E-03	BGLAP,SPP1	
DTX1	transcription regulator		4.1E-03	AKT2,SPP1	
ECSIT	transcription regulator		4.1E-03	PIM1,PLAUR	
PROX1	transcription regulator		4.1E-03	AKT2,LYVE1	
STAT6	transcription regulator	-0.339	4.1E-03	FCER2,FCGR3A/FCGR3B,IMPDH1,LTA,SERPINA1,TNFRSF8	
INSR	kinase	-0.447	4.1E-03	ALB,HSPD1,IL1RL1,LEP,PIM1,TLR2	24 (8)
ZFP36	transcription regulator		4.1E-03	PIM1,PLAUR,SPP1	24 (5)
RASSF1	other		4.1E-03	GHR,MDK,SPP1	
SAMSN1	other		4.1E-03	FCER2,INHBA,LTA	
PTH	other	-0.772	4.2E-03	BGLAP,IBSP,SOST,SPP1	22 (8)
CXCL8	cytokine		4.2E-03	COL18A1,NANOG,TNFRSF10A	
MAPK3	kinase		4.2E-03	PLAUR,SPP1,TNFRSF10A	36 (10)
Pde4	group		4.4E-03	CD163,CXCL5	
RAPGEF3	other		4.4E-03	LEP,SPP1	18 (5)
ASCL1	transcription regulator		4.5E-03	DLL1,DLL4,SEMA6B	
SP600125	chemical - kinase inhibitor	0.218	4.5E-03	BGLAP,CSF1R,INHBA,SPP1,TNFRSF10A	47 (18)
pioglitazone	chemical drug	-1.067	4.5E-03	AHSG,CFD,LEP,SOST	
MEF2	group		4.7E-03	DLL4,MB	
DLL1	enzyme		4.7E-03	DLL1,DLL4	
S100A6	transporter		4.7E-03	INHBA,TYMS	
PTCH1	transmembrane receptor		4.7E-03	DLL1,DLL4	

MAPK14	kinase	-0.849	5.0E-03	BGLAP,DLL1,PLAUR,SPP1	38 (14)
HDAC3	transcription regulator		5.1E-03	FCER2,IBSP,SPP1	16 (5)
HIF1A	transcription regulator	0.36	5.1E-03	DLL4,GHR,LEP,MB,PLAUR,TLR2	
NFAT5	transcription regulator		5.2E-03	RPS7,SPP1,TLR2	
VEGFA	growth factor	0.067	5.2E-03	DLL1,DLL4,HSD17B1,PIM1,SET	
acetaminophen	chemical drug	-0.943	5.3E-03	ALB,GHR,HSPD1,PLAUR	
okadaic acid	chemical toxicant		5.3E-03	BGLAP,PIM1,SPP1	32 (13)
phosphate	chemical - endogenous mammalian		5.3E-03	AHSG,BGLAP,SPP1	15 (5)
TNFSF14	cytokine		5.3E-03	CXCL5,INHBA	
SUMO1	enzyme		5.3E-03	ALB,NANOG	6 (2)
NF1	other		5.3E-03	KRAS,MDK	
bufalin	chemical reagent		5.3E-03	TNFRSF10A,TYMS	
diethylstilbestrol	chemical drug	0.371	5.4E-03	BGLAP,IL1R2,INHBA,LEP,PIM1	23 (6)
GATA6	transcription regulator	0.811	5.5E-03	CD163,LYVE1,MRC1,NANOG	
Stat3-Stat3	complex		5.7E-03	FCER2,LTA	
HOXC8	transcription regulator		5.7E-03	IL1R2,SPP1	
Nfat (family)	group		5.8E-03	FCER2,LTA,MB	
CEBPD	transcription regulator		5.8E-03	ALB,CSF1R,LEP	24 (5)
CEBPA	transcription regulator	0.061	6.0E-03	ALB,CFD,CSF1R,LEP,SPP1,TNFRSF10A	31 (9)
PRKAR1A	kinase		6.0E-03	BGLAP,LEP	15 (5)
nitroarginine	chemical reagent		6.0E-03	ALB,SPP1	
BCR-ABL1	fusion gene/product		6.1E-03	IFNAR1,PIM1,TNFRSF10A	
FOXO1	transcription regulator	-0.937	6.2E-03	ALB,BGLAP,HDAC8,HSPD1,LEP,MB	11 (3)
NFATC1	transcription regulator		6.3E-03	FCER2,SLAMF6,SPP1	
NFATC2	transcription regulator		6.3E-03	FCER2,INHBA,SLAMF6,TLR2	5 (2)
Hmgn3	other		6.4E-03	AHSG,SERPINA1	
TGFBR2	kinase		6.4E-03	IL1R2,KRAS,PRSS1,SPP1	
GLI2	transcription regulator		6.4E-03	IL1RL1,NANOG,SPP1	
CAMP	other		6.4E-03	CXCL5,IL1R2,TLR2	26 (7)
PTGS2	enzyme	0.106	6.5E-03	CD163,CXCL5,LEP,SOST	
prostaglandin E2	chemical - endogenous mammalian		6.6E-03	CXCL5,IBSP,IL1R2,MRC1,SPP1	30 (9)
NOS2	enzyme	0	6.6E-03	CFD,GDNF,LEP,MB	
mifepristone	chemical drug	1.3	6.6E-03	COL18A1,HSPD1,KRAS,LRP1,SPP1	
RELA	transcription regulator	-0.039	6.7E-03	CXCL5,LTA,NANOG,STK17B,TLR2,TNFRSF10A	20 (4)
interferon alfacon-1	biologic drug		6.8E-03	IL1R2,STK17B	
KDM6A	enzyme		6.8E-03	ALB,F2	
PLAAT4	enzyme		6.8E-03	IFNA7,KRAS	
MIR124	group		6.8E-03	NANOG,SET	

GJA1	transporter		6.8E-03	BGLAP,SPP1	
EPHX2	enzyme		6.8E-03	DLL1,DLL4	
RXRG	ligand-dependent nuclear receptor		6.8E-03	BGLAP,FABP3	
methylnitrosourea	chemical toxicant		6.8E-03	KRAS,PLAUR	
lysophosphatidic acid	chemical - other		6.8E-03	AKT2,GDNF,PLAUR	
hemin	chemical - endogenous mammalian		6.8E-03	CD163,MRC1,SPP1	
Histone h3	group		6.9E-03	BGLAP,CSF1R,GHR,NANOG,SPP1,TLR2	13 (2)
5-N-ethylcarboxamido adenosine	chemical reagent		7.0E-03	ALB,GDNF,INHBA	
IL12 (complex)	complex	1.188	7.0E-03	LTA,PIM1,SET,TLR2	8 (3)
ID2	transcription regulator		7.0E-03	DLL1,DLL4,LTA,SLAMF6	
L-isoleucine	chemical - endogenous mammalian		7.0E-03	ALB	
L-leucine	chemical - endogenous mammalian		7.0E-03	ALB	
gentamicin C1	chemical drug		7.0E-03	SPP1	
1-alpha,24(R),25-trihydroxyvitamin D3	chemical - endogenous mammalian		7.0E-03	BGLAP	
24R,25-dihydroxyvitamin D3	chemical - endogenous mammalian		7.0E-03	BGLAP	
chlorhexidine	chemical drug		7.0E-03	LEP	
interferon gamma-1b	biologic drug		7.0E-03	CD163	
NUMB/NUMBL	group		7.0E-03	MSTN	
enkephalin, methionine	biologic drug		7.0E-03	MRC1	
anthocyanins	chemical drug		7.0E-03	LEP	
smilagenin	chemical drug		7.0E-03	GDNF	
MARCHF2	enzyme		7.0E-03	TNFRSF10A	
CTDSPL2	phosphatase		7.0E-03	BGLAP	
STAT3/5	group		7.0E-03	PIM1	
quizartinib	chemical drug		7.0E-03	HDAC8	
triphenyltin chloride	chemical reagent		7.0E-03	NADPH	
Til1	other		7.0E-03	SPP1	
SETBP1	transcription regulator		7.0E-03	SET	
RAD23B	other		7.0E-03	NANOG	
ITGA8	other		7.0E-03	GDNF	
mir-622	microRNA		7.0E-03	PLAUR	
mir-331	microRNA		7.0E-03	PLAUR	
miR-499-5p (and other	mature microRNA		7.0E-03	IFNAR1	

miRNAs w/seed UAAGACU)						
OSTF1	transcription regulator			7.0E-03	BGLAP	
PGD	enzyme			7.0E-03	NADPH	
ING2	transcription regulator			7.0E-03	SPP1	
TLE6	other			7.0E-03	BGLAP	
CUX2	transcription regulator			7.0E-03	DLL1	
NPFFR2	G-protein coupled receptor			7.0E-03	LEP	
GNG3	enzyme			7.0E-03	LEP	
NPNT	other			7.0E-03	GDNF	
ralimetinib	chemical drug			7.0E-03	SPP1	
bivalirudin	biologic drug			7.0E-03	F2	
p-dimethylaminoazobenzene	chemical toxicant			7.0E-03	ALB	
nortriptyline	chemical drug			7.0E-03	GDNF	
terpenoid	chemical - endogenous mammalian			7.0E-03	LRP1	
D-JNK inhibitor I	biologic drug			7.0E-03	CSF1R	
alsterpaullone	chemical reagent			7.0E-03	TNFRSF10A	
tyrphostin 47	chemical - kinase inhibitor			7.0E-03	HSD17B1	
15-(S)-hydroperoxyeicosatetraenoic acid	chemical - endogenous mammalian			7.0E-03	F2	
digitoxin	chemical drug			7.0E-03	TNFRSF10A	
Ap2	group			7.2E-03	ALB,HSD17B1	
MARK2	kinase			7.2E-03	LEP,LTA	
SELP	transmembrane receptor			7.2E-03	IL1R2,PLAUR	
CYP27B1	enzyme			7.2E-03	SPP1,TLR2	14 (4)
Klrk1	transmembrane receptor			7.2E-03	IL1RL1,SPP1	
FGFR3	kinase			7.5E-03	DLL1,SPP1	
TIMP1	cytokine			7.5E-03	FCER2,PLAUR	
TYK2	kinase			7.5E-03	IFNAR1,LILRB2	
mir-1	microRNA			7.7E-03	HSPD1,KRAS,NADPH	
Rxr	group			7.9E-03	BGLAP,LEP,SPP1	23 (7)
5-fluorouracil	chemical drug	Activated	2.116	7.9E-03	CSF1R,DLL1,DLL4,KRAS,TYMS	
mir-31	microRNA			8.0E-03	SOST,SPP1	
MMP2	peptidase			8.0E-03	COL18A1,IBSP	
IL27RA	transmembrane receptor			8.0E-03	DLL4,LTA	
hyaluronic acid	chemical - endogenous mammalian			8.2E-03	CSF1R,SPP1,TLR2	40 (15)
Jnk	group		1	8.3E-03	GDNF,MRC1,PLAUR,TNFRSF10A	35 (7)

ANGPT2	growth factor	-1.067	8.3E-03	CD163,COL18A1,HSPD1,LEP	
ETS1	transcription regulator		8.3E-03	CSF1R,SLAMF6,SPP1,TYMS	
JAG2	growth factor		8.4E-03	CXCL5,SPP1	
vitamin A	chemical - endogenous mammalian		8.4E-03	LEP,NANOG	
naringenin	chemical - endogenous non-mammalian		8.4E-03	CXCL5,TLR2	
ethionine	chemical toxicant		8.4E-03	NANOG,SPP1	
CD40	transmembrane receptor	1.811	8.4E-03	FCER2,LTA,PIM1,TLR2	48 (16)
AGN194204	chemical drug	1	8.4E-03	IL1R2,LTA,PIM1,SPP1	
TGFB2	growth factor		8.4E-03	BGLAP,INHBA,SPP1	32 (10)
Esrra	transcription regulator		8.4E-03	BGLAP,IBSP,SPP1	
thalidomide	chemical drug		8.8E-03	PIM1,PLAUR	
atorvastatin	chemical drug	1	8.8E-03	LEP,PDXK,SPP1,TLR2	19 (5)
E2F1	transcription regulator	1.96	8.9E-03	BGLAP,HSPD1,KRAS,LEP,STK17B,TYMS	8 (2)
POU2F1	transcription regulator		9.0E-03	CSF1R,SPP1,TNFRSF10A	
RNASEH2A	enzyme		9.2E-03	IFNAR1,TLR2	
SAA1	transporter		9.2E-03	BGLAP,MRC1	
lactic acid	chemical - endogenous mammalian		9.2E-03	MRC1,NADPH	
bafilomycin A1	chemical drug		9.2E-03	DLL4,TNFRSF10A	
MTOR	kinase	-0.256	9.2E-03	CSF1R,LEP,MB,SERPINA1,TNFRSF10A	26 (4)
TGM2	enzyme	-0.577	9.3E-03	LILRB2,PIM1,SEMA6B,SPP1	
RARA	ligand-dependent nuclear receptor		9.4E-03	CXCL5,GHR,NANOG,SPP1,TNFRSF10A	34 (9)
DUSP1	phosphatase		9.4E-03	MRC1,PLAUR,TLR2	8 (3)
HNF1B	transcription regulator		9.4E-03	ALB,PLAUR,SERPINA1	
SAA	group		9.6E-03	MRC1,TLR2	
phenacetin	chemical drug		9.6E-03	MRC1,SPP1	
mir-150	microRNA		1.0E-02	AKT2,DLL4	
NFYC	transcription regulator		1.0E-02	ALB,TYMS	
SELPLG	other		1.0E-02	IL1R2,PLAUR	
THBS1	other		1.0E-02	LRP1,TNFRSF10A	
FOXP1	transcription regulator		1.0E-02	CSF1R,NANOG	
H-7	chemical - kinase inhibitor		1.0E-02	FCER2,PIM1	
exenatide	biologic drug		1.0E-02	MRC1,SOST	
mir-155	microRNA		1.0E-02	CD163,FCER2,MRC1	

RUNX3	transcription regulator	1.1E-02	BGLAP,CSF1R,SPP1
trapidil	chemical drug	1.1E-02	PIM1
perindoprilat	chemical drug	1.1E-02	LRP1
MRAP2	other	1.1E-02	LEP
RAI1	other	1.1E-02	LEP
Aldosterone-MR dimer	complex	1.1E-02	KRAS
bis(4-hydroxycinnamoyl)methane	chemical - endogenous non-mammalian	1.1E-02	TLR2
methylamphotericin B	chemical drug	1.1E-02	SPP1
SERTAD1	transcription regulator	1.1E-02	SET
TBX6	transcription regulator	1.1E-02	DLL1
FCGRT	transmembrane receptor	1.1E-02	ALB
riociguat	chemical drug	1.1E-02	SPP1
PLSCR3	enzyme	1.1E-02	LEP
NEIL1	enzyme	1.1E-02	LEP
SERPINB3	other	1.1E-02	LRP1
SPEN	transcription regulator	1.1E-02	BGLAP
SENP2	peptidase	1.1E-02	MSTN
INHBC	growth factor	1.1E-02	INHBA
MTHFD2	enzyme	1.1E-02	NANOG
ASGR2	transmembrane receptor	1.1E-02	ASGR1
cetuximab/radiotherapy	biologic drug	1.1E-02	TYMS
Gm33994	other	1.1E-02	NANOG
G3BP2	enzyme	1.1E-02	NANOG
GIMAP1-GIMAP5	other	1.1E-02	HSPD1
AZ-960	chemical reagent	1.1E-02	PIM1
SK & F 86002	chemical - kinase inhibitor	1.1E-02	FCER2
CVT-313	chemical - kinase inhibitor	1.1E-02	NANOG
N-ethylmaleimide	chemical reagent	1.1E-02	GHR
protein phosphatase	complex	1.1E-02	PIM1
cabergoline	chemical drug	1.1E-02	GDNF
des-Arg-9 bradykinin	chemical - endogenous mammalian	1.1E-02	CXCL5
ropinirole	chemical drug	1.1E-02	GDNF
benserazide	chemical drug	1.1E-02	GDNF

1beta,25-dihydroxyvitamin D3	chemical drug		1.1E-02	BGLAP
PRMT5	enzyme		1.1E-02	LEP,NANOG
LHCGR	G-protein coupled receptor		1.1E-02	HSD17B1,IL1R2
4-methylnitrosoamino-1-(3-pyridinyl)-1-butanone	chemical toxicant		1.1E-02	SERPINA1,SPP1
estrogen receptor	group	0.277	1.1E-02	AKT2,CDH15,PLAUR,SOST
simvastatin	chemical drug	0.283	1.1E-02	AHSG,SPP1,TLR2,TYMS
TMPRSS2-ERG	fusion gene/product		1.1E-02	INHBA,PIM1,TLR2
epigallocatechin-gallate	chemical drug	-0.958	1.1E-02	COL18A1,CXCL5,LEP,TNFRSF10A
DLL4	other		1.1E-02	DLL4,LYVE1
HR	transcription regulator		1.1E-02	BOC,IL1R2
ELANE	peptidase		1.1E-02	CXCL5,SERPINA1
ILK	kinase		1.1E-02	KRAS,NANOG
TLR9	transmembrane receptor	-0.068	1.1E-02	FCER2,GDNF,IFNA7,LTA
ESR1	ligand-dependent nuclear receptor	-0.647	1.1E-02	AKT2,CLEC1B,DLL1,GHR,HSPD1,KRAS,LEP,PLAUR,SOST,SPP1,TNFRSF10A
GATA3	transcription regulator		1.1E-02	CSF1R,HSD17B1,IL1RL1,LTA
cisplatin	chemical drug	-0.16	1.1E-02	ALB,CXCL5,GHR,MRC1,NTN4,SPP1,STK17B,TYMS
HRH3	G-protein coupled receptor		1.2E-02	CFD,LEP
SMAD1	transcription regulator		1.2E-02	IBSP,SPP1
tempol	chemical drug		1.2E-02	PLAUR,SPP1
SMAD4	transcription regulator		1.2E-02	BGLAP,IL17B,KLK11,TNFRSF10A
docetaxel	chemical drug		1.2E-02	PIM1,TNFRSF10A,TYMS
EBF1	transcription regulator		1.2E-02	AKT2,KRAS,TLR2
MEF2C	transcription regulator		1.2E-02	BGLAP,IBSP,SOST
SATB2	transcription regulator		1.2E-02	NANOG,SPP1
XDH	enzyme		1.2E-02	PLAUR,SPP1
SERPINA1	other		1.2E-02	FCER2,TLR2
LTA	cytokine		1.2E-02	LTA,LYVE1
RUNX1	transcription regulator		1.2E-02	ALB,BGLAP,CSF1R,SPP1
methylselenic acid	chemical reagent		1.2E-02	AKT2,CDH15,TLR2,TYMS
IL33	cytokine		1.2E-02	IL1RL1,MDK,SOST,TLR2
reactive oxygen species	chemical toxicant		1.3E-02	CSF1R,LEP,TLR2

tacrolimus	chemical drug	1.3E-02	HSPD1,IL1R2,IL1RL1,SPP1
2-bromoethylamine	chemical reagent	1.3E-02	MRC1,SPP1
SOX11	transcription regulator	1.3E-02	HSPD1,IL1RL1,KLK11
IL22	cytokine	1.3E-02	CXCL5,GRP,S100A7
TRPS1	transcription regulator	1.4E-02	BGLAP,IBSP
C/EBP	group	1.4E-02	CSF1R,LEP
SP110	transcription regulator	1.4E-02	DLL4,IFNAR1,SERPINA1
DGCR8	enzyme	1.4E-02	GDNF,KRAS
CERK	kinase	1.4E-02	CCL28,TLR2
2-(3-hydroxypropoxy)calcitriol	chemical drug	1.4E-02	BGLAP
FEZF1	other	1.4E-02	KRAS
SPRED1	other	1.4E-02	KRAS
DAND5	other	1.4E-02	NANOG
LY75	transmembrane receptor	1.4E-02	FCER2
SLAMF7	other	1.4E-02	LTA
BBS10	other	1.4E-02	LEP
KBTBD2	other	1.4E-02	LEP
RSPO2	other	1.4E-02	IBSP
TXLNG	other	1.4E-02	BGLAP
TMEM119	other	1.4E-02	BGLAP
SLURP1	cytokine	1.4E-02	LEP
RAB7	group	1.4E-02	CXCL5
CDH3	other	1.4E-02	CDH15
KPNA4	transporter	1.4E-02	LTA
BRS3	G-protein coupled receptor	1.4E-02	LEP
miR-331-3p (miRNAs w/seed CCCCUGG)	mature microRNA	1.4E-02	PLAUR
miR-1285-3p (and other miRNAs w/seed CUGGGCA)	mature microRNA	1.4E-02	AKT2
NEDD8	enzyme	1.4E-02	IFNAR1
LAMB1	other	1.4E-02	DLL4
TXLNA	cytokine	1.4E-02	LTA
anatabine	chemical - endogenous non-mammalian	1.4E-02	IL1R2
lanatoside C	chemical - endogenous non-mammalian	1.4E-02	TNFRSF10A
CIDEB	other	1.4E-02	LEP
PDIA3	peptidase	1.4E-02	FCGR3A/FCGR3B
HELZ2	transcription regulator	1.4E-02	LEP
SLC25A12	transporter	1.4E-02	NADPH
ALPL	phosphatase	1.4E-02	SPP1
HOXD11	transcription regulator	1.4E-02	GDNF

NUCKS1	kinase		1.4E-02	LEP
RAPGEF4	other		1.4E-02	SPP1
TKT	enzyme		1.4E-02	LEP
LIFR	transmembrane receptor		1.4E-02	SOST
SLC20A2	transporter		1.4E-02	SPP1
HAP1	other		1.4E-02	LEP
amprenavir	chemical drug		1.4E-02	LEP
polymethyl methacrylate	chemical reagent		1.4E-02	CSF1R
5-iodo-6-amino-1,2-benzopyrone	chemical reagent		1.4E-02	NADPH
natamycin	chemical drug		1.4E-02	SPP1
incyclinide	chemical drug		1.4E-02	TYMS
adapalene	chemical drug		1.4E-02	TLR2
sodium phosphate	chemical drug		1.4E-02	SPP1
miR-499a-5p inhibitor	chemical reagent		1.4E-02	IFNAR1
miR-208b inhibitor	chemical reagent		1.4E-02	IFNAR1
glutathione diethyl ester	chemical reagent		1.4E-02	PLAUR
dobutamine	chemical drug		1.4E-02	LEP
pentanoic acid	chemical - endogenous mammalian		1.4E-02	LEP
oxamic acid	chemical - endogenous non-mammalian		1.4E-02	INHBA
oleandrin	chemical drug		1.4E-02	TNFRSF10A
trichostatin A	chemical drug	0.415	1.4E-02	AKT2,GHR,MRC1,PIM1,SPP1,TLR2,TNFRSF10A,TYMS
KITLG	growth factor		1.4E-02	CLEC1B,IL1RL1,PIM1,RPS7
TLR3	transmembrane receptor		1.4E-02	GDNF,IFNA7,IFNAR1,IL1R2
SQSTM1	transcription regulator		1.5E-02	LEP,LTA
ZBTB16	transcription regulator		1.5E-02	BGLAP,SLAMF6,SPP1
ROCK2	kinase		1.5E-02	S100A7,SPP1
PRMT1	enzyme		1.5E-02	MRC1,NANOG
IL27	cytokine		1.6E-02	CD163,MRC1,PIM1
Histone h4	group		1.6E-02	BGLAP,SPP1,TYMS
PSEN1	peptidase		1.6E-02	ALB,DLL1,HSD17B1,HSPD1,LRP1
triptolide	chemical drug		1.6E-02	DLL4,GHR,TLR2
E. coli B5 lipopolysaccharide	chemical - endogenous non-mammalian	-1.172	1.6E-02	AHSG,IL1R2,SPP1,TLR2
pyridaben	chemical toxicant		1.6E-02	CRLF2,INHBA
RETN	other		1.6E-02	FABP3,SPP1

carboplatin	chemical drug		1.6E-02	MRC1,SPP1
zVAD-FMK	chemical - protease inhibitor		1.6E-02	CD163,MRC1
palmitic acid	chemical - endogenous mammalian	-0.225	1.6E-02	AHSG,INHBA,LEP,TLR2
Rock	group		1.7E-02	IBSP,SPP1
MAP3K1	kinase		1.7E-02	PLAUR,TNFRSF10A
genistein	chemical drug	-0.555	1.7E-02	CFD,GDNF,PIM1,SPP1,STK17B
MOG	other		1.7E-02	GDNF,LEP
hexachlorobenzene	chemical toxicant		1.7E-02	MRC1,SPP1
candesartan	chemical drug		1.7E-02	LEP,SPP1
valsartan	chemical drug		1.7E-02	LEP,SPP1
N-acetyl-D-mannosamine	chemical - endogenous mammalian		1.8E-02	BGLAP
prostaglandin E	chemical - endogenous mammalian		1.8E-02	SOST
dienogest	chemical drug		1.8E-02	HSD17B1
norethindrone acetate	chemical drug		1.8E-02	F2
ILX-23-7553	chemical drug		1.8E-02	BGLAP
Integrinα	group		1.8E-02	TLR2
catalase	group		1.8E-02	NANOG
NEUROD4	transcription regulator		1.8E-02	DLL1
FOXN4	transcription regulator		1.8E-02	DLL4
TRIM58	other		1.8E-02	TLR2
ARID2	transcription regulator		1.8E-02	BGLAP
receptor protein tyrosine kinase	group		1.8E-02	SPP1
asparagine	chemical - endogenous mammalian		1.8E-02	ALB
CACNA1B	ion channel		1.8E-02	LEP
MARCHF8	enzyme		1.8E-02	TNFRSF10A
MC3R	G-protein coupled receptor		1.8E-02	LEP
NACA	transcription regulator		1.8E-02	SOST
ANKH	transporter		1.8E-02	SPP1
SSTR5	G-protein coupled receptor		1.8E-02	LEP
D-mannosamine	chemical - endogenous mammalian		1.8E-02	BGLAP
CCKAR	G-protein coupled receptor		1.8E-02	LEP

GTF3A	transcription regulator		1.8E-02	PIM1
MATK	kinase		1.8E-02	IBSP
Wfdc17	other		1.8E-02	MRC1
LNPEP	peptidase		1.8E-02	LEP
MARCHF1	enzyme		1.8E-02	TNFRSF10A
unsaturated guluronate oligosaccharide	chemical reagent		1.8E-02	FCGR3A/FCGR3B
CHCHD2	other		1.8E-02	NANOG
tideglusib	chemical drug		1.8E-02	NANOG
farnesyltransferase inhibitor	chemical drug		1.8E-02	KRAS
lonidamine	chemical drug		1.8E-02	NADPH
tirapazamine	chemical drug		1.8E-02	SPP1
butylphen	chemical toxicant		1.8E-02	TNFRSF10A
tyrphostin 25	chemical - kinase inhibitor		1.8E-02	HSD17B1
vidarabine	chemical drug		1.8E-02	FABP3
ecdysterone	chemical - endogenous mammalian		1.8E-02	BGLAP
PAX5	transcription regulator		1.8E-02	CSF1R,FCER2
CBL	transcription regulator		1.8E-02	BGLAP,LEP
Pln	other		1.8E-02	AHSG,FABP3
LEF1	transcription regulator		1.8E-02	BGLAP,NTN4,SPP1
morphine	chemical drug		1.8E-02	GHR,LEP,TLR2
JAK2	kinase		1.8E-02	NANOG,SEMA6B,SET
ZBTB20	transcription regulator		1.8E-02	GHR,LEP
MAPKAPK2	kinase		1.8E-02	LTA,MRC1
PTPN1	phosphatase		1.8E-02	FCER2,LEP
ADRB2	G-protein coupled receptor		1.8E-02	LEP,SPP1
IGF1R	transmembrane receptor		1.9E-02	COL18A1,INHBA,NANOG,TYMS
cytokine	group		1.9E-02	LEP,PLAUR,TLR2
MYCN	transcription regulator	0.218	1.9E-02	COL18A1,HSPD1,INHBA,RPS7
HSP90B1	other		1.9E-02	CXCL5,TLR2
AKT2	kinase		1.9E-02	AKT2,LEP
RNF2	transcription regulator		1.9E-02	NANOG,SERPINA1
geldanamycin	chemical - endogenous non-mammalian	-1.091	1.9E-02	AKT2,HSPD1,IL1RL1,TYMS
CAV1	transmembrane receptor		1.9E-02	CSF1R,INHBA,LEP
dextran sulfate	chemical drug		1.9E-02	CFH,COL18A1,MRC1,TLR2

HIC1	transcription regulator		2.0E-02	INHBA,SPP1
phenylbutazone	chemical drug		2.0E-02	MRC1,SPP1
H89	chemical - kinase inhibitor		2.0E-02	BGLAP,GDNF,IBSP
quercetin	chemical drug		2.0E-02	LEP,NADPH,PLAUR
aldosterone	chemical - endogenous mammalian		2.0E-02	KRAS,PLAUR,SPP1
PRL	cytokine	-0.152	2.0E-02	HSPD1,LYVE1,PIM1,SPP1
S-nitroso-N-acetyl-DL-penicillamine	chemical reagent		2.0E-02	MB,SPP1
mir-181	microRNA		2.0E-02	CD163,NANOG
seocalcitol	chemical drug		2.0E-02	BGLAP,LRP1
WNT3A	cytokine	1.951	2.0E-02	BGLAP,CFD,GDNF,NANOG
NFKB1	transcription regulator		2.1E-02	DLL4,LTA,PIM1,TLR2
CD38	enzyme		2.1E-02	IL1R2,LTA,PIM1
EP300	transcription regulator		2.1E-02	LEP,LTA,NANOG,SLAMF6,TLR2
RHO	G-protein coupled receptor		2.1E-02	CSF1R,IMPDH1
PPP3R1	phosphatase		2.1E-02	IL1RL1,LTA
heme	chemical - endogenous mammalian		2.1E-02	CD163,MRC1
DCAF13	other		2.1E-02	NANOG
AMELY	growth factor		2.1E-02	BGLAP
CCDC3	other		2.1E-02	LEP
YOD1	enzyme		2.1E-02	INHBA
WDFY2	other		2.1E-02	AKT2
FLT4	transmembrane receptor		2.1E-02	BGLAP
Ap2 alpha	group		2.1E-02	PLAUR
PRKD2	kinase		2.1E-02	IFNAR1
Msx3	transcription regulator		2.1E-02	MRC1
RBBP4	enzyme		2.1E-02	KRAS
Pzp	other		2.1E-02	LRP1
BCL2L12	other		2.1E-02	TNFRSF10A
miR-3118 (and other miRNAs w/seed GUGACUG)	mature microRNA		2.1E-02	NANOG
SOX18	transcription regulator		2.1E-02	DLL4
RSC1A1	other		2.1E-02	LEP
TAF10	transcription regulator		2.1E-02	GHR
ETV3	transcription regulator		2.1E-02	SPP1
MMP13	peptidase		2.1E-02	IBSP
BCAT2	enzyme		2.1E-02	LEP
PRLHR	G-protein coupled receptor		2.1E-02	LEP
ATP6V0A2	transporter		2.1E-02	MRC1

PDE10A	enzyme		2.1E-02	LEP
WAC	other		2.1E-02	BGLAP
DSPP	other		2.1E-02	BGLAP
PLAAT3	enzyme		2.1E-02	LEP
SRGN	other		2.1E-02	NANOG
ERCC3	enzyme		2.1E-02	GHR
mianserin	chemical drug		2.1E-02	GDNF
chlorambucil	chemical drug		2.1E-02	TNFRSF10A
risedronic acid	chemical drug		2.1E-02	HSPD1
Ki16425	chemical reagent		2.1E-02	GDNF
aurintricarboxylic acid	chemical drug		2.1E-02	PIM1
15-hydroxyeicosatetraenoic acid	chemical - endogenous mammalian		2.1E-02	F2
phenylamil	chemical reagent		2.1E-02	BGLAP
IFNB1	cytokine	0.277	2.1E-02	DLL1,IFNAR1,SPP1,TNFRSF10A
TWIST1	transcription regulator		2.1E-02	AKT2,BGLAP,SPP1
napabucasin	chemical drug		2.2E-02	NANOG,PLAUR
camptothecin	chemical drug		2.2E-02	AKT2,LILRB2,SPP1,STK17B,TLR2,TNFRSF10A
bisphenol A	chemical - endogenous mammalian		2.2E-02	CFD,LEP,STK17B
STK11	kinase	1	2.2E-02	ALB,SPP1,STK17B,TYMS
Ap1	complex		2.2E-02	BGLAP,RSPO3,TNFRSF8
12-(3-adamantan-1-yl-ureido) dodecanoic acid	chemical reagent		2.2E-02	LTA,TNFRSF10A
FOXL2	transcription regulator		2.2E-02	INHBA,RSPO3
SMPD1	enzyme		2.2E-02	CCL28,TLR2
NFE2L2	transcription regulator	-0.692	2.2E-02	BGLAP,CFD,ENTPD5,GHR,IMPDH1
Salmonella enterica serotype abortus equi lipopolysaccharide	chemical toxicant		2.2E-02	ABL2,CXCL5,INHBA
EPO	cytokine		2.2E-02	IL1RL1,PIM1,RPS7,SPP1
DNMT3A	enzyme		2.3E-02	CSF1R,NANOG,SPP1
tributyrin	chemical drug		2.3E-02	CXCL5,INHBA
FGF21	growth factor		2.3E-02	GHR,LEP
NR4A3	ligand-dependent nuclear receptor		2.3E-02	MB,MSTN
triamterene	chemical drug		2.3E-02	MRC1,SPP1
Tgf beta	group		2.3E-02	BGLAP,CSF1R,SPP1,TLR2
maneb	chemical toxicant		2.3E-02	CRLF2,INHBA

RARB	ligand-dependent nuclear receptor		2.4E-02	NANOG,SERPINA1,SPP1
AKT1	kinase		2.4E-02	LEP,MSTN,SPP1,TYMS
kainic acid	chemical toxicant		2.4E-02	CSF1R,GDNF,SPP1
ERK1/2	group	-1.067	2.4E-02	CXCL5,NANOG,PLAUR,SPP1
Fcer1	complex		2.4E-02	INHBA,MRC1
GNAS	enzyme		2.4E-02	LEP,SPP1
CD3E	transmembrane receptor		2.4E-02	KRAS,LTA
Interferon alpha	group	-0.337	2.4E-02	CXCL5,IFNAR1,PIM1,TLR2,TYMS
raloxifene	chemical drug		2.4E-02	BGLAP,MB,SPP1
L-proline	chemical - endogenous mammalian		2.4E-02	ALB
BBS12	other		2.4E-02	LEP
Stat1 dimer	complex		2.4E-02	PIM1
Glucocorticoid-GCR	complex		2.4E-02	BGLAP
SLC5A8	transporter		2.4E-02	TNFRSF10A
SFRP5	transmembrane receptor		2.4E-02	AKT2
dimethyl sulfone	chemical drug		2.4E-02	GHR
SOX2-OCT4	complex		2.4E-02	NANOG
valine	chemical - endogenous mammalian		2.4E-02	ALB
PYY	other		2.4E-02	LEP
TRPV2	ion channel		2.4E-02	AKT2
Akr1b7	enzyme		2.4E-02	LEP
ULBP2	transmembrane receptor		2.4E-02	LTA
CD55	other		2.4E-02	ALB
mir-296	microRNA		2.4E-02	PRSS1
miR-184 (and other miRNAs w/seed GGACGGA)	mature microRNA		2.4E-02	AKT2
OGN	growth factor		2.4E-02	BGLAP
PRKAB1	kinase		2.4E-02	LEP
KHDRBS1	transcription regulator		2.4E-02	LEP
TRHR	G-protein coupled receptor		2.4E-02	LEP
G3BP1	enzyme		2.4E-02	SPP1
FZD5	G-protein coupled receptor		2.4E-02	SPP1
STEAP4	enzyme		2.4E-02	LEP
MAD2L2	enzyme		2.4E-02	NANOG
SHCBP1	other		2.4E-02	NANOG
SNW1	transcription regulator		2.4E-02	BGLAP
FBLN5	other		2.4E-02	SERPINA1
glyceraldehyde-BSA	chemical reagent		2.4E-02	LEP

methylglyoxal-BSA	chemical reagent		2.4E-02	LEP
Npy4r	G-protein coupled receptor		2.4E-02	LEP
HOXC11	transcription regulator		2.4E-02	GDNF
ANXA6	ion channel		2.4E-02	BGLAP
PSENEN	peptidase		2.4E-02	DLL1
amoxicillin	chemical drug		2.4E-02	TLR2
FM19G11	chemical reagent		2.4E-02	NANOG
aminooxyacetic acid	chemical reagent		2.4E-02	NADPH
cinnamyl-3,4-dihydroxy-alpha-cyanocinnamate	chemical reagent		2.4E-02	MRC1
bupropion	chemical drug		2.4E-02	TNFRSF10A
3-hydroxymorphinan	chemical - endogenous mammalian		2.4E-02	GDNF
sodium fluoride	chemical drug		2.4E-02	LEP
leucovorin	chemical - endogenous mammalian		2.4E-02	TYMS
murabutide	chemical reagent		2.4E-02	MRC1
vinorelbine	chemical drug		2.4E-02	TYMS
20alpha-hydroxycholesterol	chemical - endogenous mammalian		2.4E-02	BGLAP
DOCK8	other		2.5E-02	INHBA,LTA
PDGFB	growth factor		2.5E-02	BGLAP,SPP1
mir-15	microRNA		2.5E-02	PIM1,PLAUR
BCL2L1	other		2.5E-02	LTA,TNFRSF10A
EFNA1	other		2.5E-02	KRAS,S100A7
fenamic acid	chemical reagent		2.5E-02	MRC1,SPP1
ATP-gamma-S	chemical reagent		2.5E-02	CSF1R,IL1R2
infliximab	biologic drug		2.5E-02	INHBA,LTA
TBX21	transcription regulator		2.5E-02	IL1RL1,SPP1
ID1	transcription regulator		2.6E-02	NANOG,TYMS
DKK1	growth factor		2.6E-02	BGLAP,SOST
SASH1	other		2.6E-02	INHBA,LTA
entinostat	chemical drug		2.6E-02	LYVE1,TNFRSF10A
glucosamine	chemical - endogenous mammalian		2.6E-02	BGLAP,LEP
CD3	complex	0.088	2.6E-02	CRLF2,LRP1,LTA,PRSS1,TLR2,TNFRSF8

2-(4-amino-1-isopropyl-1H-pyrazolo[3,4-d]pyrimidin-3-yl)-1H-indol-5-ol	chemical reagent		2.7E-02	INHBA,PIM1,TYMS
doxorubicin	chemical drug	-0.128	2.7E-02	ALB,CXCL5,MRC1,SPP1,TNFRSF10A
RXRB	ligand-dependent nuclear receptor		2.7E-02	BGLAP,SPP1
ETS2	transcription regulator		2.7E-02	CSF1R,SPP1
gentamicin C	chemical drug		2.7E-02	MRC1,SPP1
GNA14	enzyme		2.7E-02	FCGR3A/FCGR3B,PLAUR
mir-34	microRNA		2.7E-02	CSF1R,DLL1
KLF2	transcription regulator		2.8E-02	DLL1,IL1RL1,TNFRSF8
L-phenylalanine	chemical - endogenous mammalian		2.8E-02	ALB
GPIIB-IIIA	complex		2.8E-02	PLAUR
cAMP-Gef	group		2.8E-02	CXCL5
ibudilast	chemical drug		2.8E-02	GDNF
DRD3	G-protein coupled receptor		2.8E-02	LEP
LGALS12	other		2.8E-02	LEP
DLX6-AS1	other		2.8E-02	NANOG
ANGPTL6	other		2.8E-02	LEP
ITPRID2	other		2.8E-02	LEP
TRPC4AP	transporter		2.8E-02	HSPD1
FNIP1	other		2.8E-02	MB
SERPINA12	other		2.8E-02	LEP
PDS5B	other		2.8E-02	NANOG
strontium ranelate	chemical drug		2.8E-02	SOST
GUCY2C	kinase		2.8E-02	LEP
RNF40	enzyme		2.8E-02	BGLAP
U1 snRNP	complex		2.8E-02	LY9
ERCC4	enzyme		2.8E-02	GHR
miR-192-5p (and other miRNAs w/seed UGACCUA)	mature microRNA		2.8E-02	TYMS
DKK2	other		2.8E-02	SPP1
PPP1R3C	phosphatase		2.8E-02	LEP
ERCC1	enzyme		2.8E-02	GHR
LDHB	enzyme		2.8E-02	MB
P2RY1	G-protein coupled receptor		2.8E-02	LEP
DES	other		2.8E-02	SPP1
APCS	other		2.8E-02	CD163
LAMA4	enzyme		2.8E-02	DLL4
INPP5K	phosphatase		2.8E-02	LEP
PLIN2	other		2.8E-02	LEP
GRB10	other		2.8E-02	MRC1
PPP1CA	phosphatase		2.8E-02	SPP1
Apoc3	transporter		2.8E-02	LEP

GADD45GIP1	other		2.8E-02	HSPD1
PCBP1	translation regulator		2.8E-02	INHBA
esculetin	chemical - endogenous non-mammalian		2.8E-02	ALB
JNJ-39933673	chemical reagent		2.8E-02	SPP1
palmitoyl-Cys((RS)-2,3-di(palmitoyloxy)-propyl)-Ala-Gly-OH	chemical reagent		2.8E-02	TLR2
dihematoporphyrin ether	chemical drug		2.8E-02	HSPD1
phytanic acid	chemical - endogenous mammalian		2.8E-02	NADPH
SHH	peptidase		2.8E-02	GDNF,SPP1,TYMS
let-7a-5p (and other miRNAs w/seed GAGGUAG)	mature microRNA		2.8E-02	F2,KRAS,TYMS
Calcineurin protein(s)	complex		2.8E-02	MB,SPP1
LGALS1	other		2.8E-02	KRAS,LEP
folic acid	chemical - endogenous mammalian		2.8E-02	COL18A1,TYMS
TP63	transcription regulator	1.459	2.8E-02	DLL1,INHBA,NANOG,S100A7,TNFRSF10A
progesterone	chemical - endogenous mammalian	-0.956	2.9E-02	ASGR1,CFD,GHR,INHBA,MRC1,SPP1
ADIPOQ	other		2.9E-02	CD163,FABP3,LEP
corticosteroid	chemical drug		3.0E-02	IL1R2,STK17B
PTHLH	other		3.0E-02	BGLAP,IBSP
isotretinoin	biologic drug		3.0E-02	S100A7,TLR2
thyroid hormone	chemical - endogenous mammalian		3.0E-02	GHR,LEP,SEMA6B
Ca2+	chemical - endogenous mammalian	0.152	3.0E-02	BGLAP,NADPH,S100A7,SPP1
IL18	cytokine		3.0E-02	FCGR3A/FCGR3B,SET,SPP1
ESRRG	ligand-dependent nuclear receptor		3.0E-02	IBSP,MB
NEUROG3	transcription regulator		3.0E-02	DLL1,DLL4
ITGB3	transmembrane receptor		3.0E-02	PLAUR,SOST
GHRL	growth factor		3.0E-02	LEP,SPP1
lysophosphatidylcholine	chemical - other		3.1E-02	LRP1,PLAUR
FOSL1	transcription regulator		3.1E-02	NANOG,PLAUR
LEPR	transmembrane receptor		3.1E-02	IL1R2,LEP,STK17B

RUNX1T1	transcription regulator	3.1E-02	CSF1R
MSGN1	transcription regulator	3.1E-02	DLL1
nebivolol	chemical drug	3.1E-02	LEP
ESRP1	other	3.1E-02	NANOG
propargylamine	chemical reagent	3.1E-02	GDNF
ZBED2	other	3.1E-02	PIM1
TFCP2L1	transcription regulator	3.1E-02	NANOG
HINT2	other	3.1E-02	LEP
GATM	enzyme	3.1E-02	LEP
KDM4B	enzyme	3.1E-02	NANOG
SMAD1/5	group	3.1E-02	PLAUR
UCP3	transporter	3.1E-02	LEP
G0S2	other	3.1E-02	HSPD1
PDGF (family)	group	3.1E-02	HSPD1
PRLH	cytokine	3.1E-02	LEP
HTR2C	G-protein coupled receptor	3.1E-02	LEP
CTSZ	peptidase	3.1E-02	LILRB2
Ccl6	cytokine	3.1E-02	SERPINA1
Irs3	other	3.1E-02	LEP
DNM1L	enzyme	3.1E-02	NANOG
TBXA2R	G-protein coupled receptor	3.1E-02	GDNF
ASF1A	other	3.1E-02	NANOG
COCH	other	3.1E-02	NANOG
GLIS1	transcription regulator	3.1E-02	NANOG
USP17L2 (includes others)	peptidase	3.1E-02	NANOG
niflumic acid	chemical drug	3.1E-02	LILRB2
raltitrexed	chemical drug	3.1E-02	TYMS
ammonium trichloro(dioxoethylene-O,O'-)tellurate	chemical drug	3.1E-02	GDNF
ziprasidone	chemical drug	3.1E-02	GDNF
floxuridine	chemical drug	3.1E-02	TYMS
aurothioglucose	chemical drug	3.1E-02	LEP
DYSF	other	3.2E-02	CFD,MRC1
PD173074	chemical reagent	3.3E-02	GDNF,NANOG
ZEB1	transcription regulator	3.3E-02	CD163,MRC1
SOX3	transcription regulator	3.3E-02	DLL1,INHBA
cyclophosphamide	chemical drug	3.3E-02	IL1R2,SPP1
INHBA	growth factor	3.3E-02	INHBA,KRAS,NANOG
RETNLB	other	3.3E-02	STK17B,TLR2
PLG	peptidase	3.3E-02	IL1RL1,MRC1

Bay 11-7082	chemical - kinase inhibitor		3.3E-02	PIM1,TLR2
TLR2	transmembrane receptor		3.4E-02	GDNF,LEP,TLR2
IFN alpha/beta	group		3.4E-02	MRC1,TLR2
MTPN	transcription regulator		3.4E-02	AHSG,SPP1
8-bromo-cAMP	chemical reagent		3.4E-02	BGLAP,HSD17B1,SPP1
valproic acid	chemical drug	0.603	3.4E-02	GDNF,IL1RL1,LEP,SPP1,TNFRSF10A
FOXO3	transcription regulator		3.4E-02	HDAC8,INHBA,MSTN,NTN4
KMT2D	transcription regulator		3.5E-02	CA10,CRLF2,FABP3
Cyclin D	group		3.5E-02	NANOG
NAP1L1	other		3.5E-02	ALB
CRTC3	other		3.5E-02	LEP
Sox2ot	other		3.5E-02	NANOG
LY6K	other		3.5E-02	NANOG
TAF5	transcription regulator		3.5E-02	GHR
DCLK1	kinase		3.5E-02	KRAS
MCHR1	G-protein coupled receptor		3.5E-02	LEP
IL9R	transmembrane receptor		3.5E-02	PIM1
HOXB5	transcription regulator		3.5E-02	PLAUR
RELN	peptidase		3.5E-02	LYVE1
SH2B1	other		3.5E-02	PLAUR
EXT1	enzyme		3.5E-02	NANOG
CCDC80	other		3.5E-02	LEP
MALAT1	other		3.5E-02	KRAS
cariporide	chemical drug		3.5E-02	SPP1
6-aminonicotinamide	chemical reagent		3.5E-02	NADPH
domoic acid	chemical toxicant		3.5E-02	KRAS
acetic acid	chemical - endogenous mammalian		3.5E-02	LEP
retinol acetate	chemical drug		3.5E-02	TNFRSF10A
mannan	chemical - endogenous mammalian		3.5E-02	TLR2
dydrogesterone	chemical drug		3.5E-02	HSD17B1
22(S)-hydroxycholesterol	chemical - endogenous mammalian		3.5E-02	BGLAP
vitamin E	chemical drug		3.5E-02	F2,SPP1
GMNN	transcription regulator		3.5E-02	INHBA,NANOG
SREBF2	transcription regulator		3.5E-02	HSD17B1,LRP1
SPP1	cytokine		3.5E-02	CD163,CXCL5,SPP1
MAP3K14	kinase		3.6E-02	IL1R2,LTA

PRNP	other	3.6E-02	LRP1,PLAUR
CCL5	cytokine	3.6E-02	CD163,PLAUR
NRAS	enzyme	3.6E-02	CFH,FCER2,KRAS
mycophenolic acid	chemical drug	3.7E-02	CXCL5,INHBA
thapsigargin	chemical toxicant	3.7E-02	AHSG,IFNAR1,TLR2
cocaine	chemical drug	3.8E-02	ASGR1,INHBA,PDXK
IL7R	transmembrane receptor	3.8E-02	PIM1,SLAMF6
CYP19A1	enzyme	3.8E-02	HSD17B1,LEP
captopril	chemical drug	3.8E-02	MRC1,SPP1
L-lysine	chemical - endogenous mammalian	3.8E-02	ALB
cosyntropin	biologic drug	3.8E-02	DLL4
FTO	enzyme	3.8E-02	LEP
HOXA-AS2	other	3.8E-02	KRAS
APOL1	transporter	3.8E-02	IFNAR1
B4GALT6	enzyme	3.8E-02	TLR2
PRPF19	enzyme	3.8E-02	NANOG
Cebp	complex	3.8E-02	ALB
ADCY7	enzyme	3.8E-02	IL1RL1
GFPT1	enzyme	3.8E-02	LEP
NRBP2	kinase	3.8E-02	NANOG
SLC16A1	transporter	3.8E-02	NADPH
SOX8	transcription regulator	3.8E-02	IBSP
DUSP4	phosphatase	3.8E-02	IL1RL1
miR-208a-3p (and other miRNAs w/seed UAAGACG)	mature microRNA	3.8E-02	SPP1
LINC-ROR	other	3.8E-02	NANOG
AIMP2	other	3.8E-02	KRAS
SART1	other	3.8E-02	NANOG
GFER	enzyme	3.8E-02	NANOG
CXCL13	cytokine	3.8E-02	LTA
PARP2	enzyme	3.8E-02	LEP
HP	peptidase	3.8E-02	CD163
BRMS1	transcription regulator	3.8E-02	SPP1
FUS	transcription regulator	3.8E-02	HSPD1
darglitazone	chemical drug	3.8E-02	LEP
doxazosin	chemical drug	3.8E-02	SPP1
itraconazole	chemical drug	3.8E-02	TLR2
ibandronic acid	chemical drug	3.8E-02	BGLAP
4-aminophenol	chemical - endogenous mammalian	3.8E-02	SPP1
dextran	chemical drug	3.8E-02	AFM
formononetin	chemical - endogenous	3.8E-02	BGLAP

	non-mammalian				
EIF4E	translation regulator			3.8E-02	CSF1R,PIM1,PLAUR
nicotine	chemical drug			3.9E-02	LEP,PLAUR,SPP1
NFYA	transcription regulator			3.9E-02	ALB,IBSP
CBFB	transcription regulator			3.9E-02	CSF1R,SPP1
TCL1A	transcription regulator			3.9E-02	IL1R2,LY9
NEDD9	other			3.9E-02	LRP1,LRRTM1
metformin	chemical drug			4.0E-02	AHSG,LEP,MSTN
SIM1	transcription regulator			4.0E-02	DLL1,GDNF,LEP
AGT	growth factor		0.899	4.0E-02	DLL4,KRAS,LEP,LTA,SPP1
ZFHX3	transcription regulator			4.0E-02	ALB,SERPINA1
NR4A2	ligand-dependent nuclear receptor			4.0E-02	ABL2,BGLAP
vancomycin	biologic drug			4.0E-02	MRC1,SPP1
ERG	transcription regulator			4.1E-02	PIM1,PLAUR,SPP1
deferoxamine	chemical drug			4.1E-02	LEP,PLAUR,SPP1
TLR7	transmembrane receptor			4.1E-02	CFH,IFNA7,LTA
adenine	chemical - endogenous mammalian			4.2E-02	NADPH
SOX2-OT	other			4.2E-02	NANOG
PP2A	complex			4.2E-02	PIM1
CLEC1B	transmembrane receptor			4.2E-02	SPP1
GPR132	G-protein coupled receptor			4.2E-02	MRC1
PLA2G1B	enzyme			4.2E-02	LEP
ACVR2A	kinase			4.2E-02	BGLAP
RYR1	ion channel			4.2E-02	MB
miR-150-5p (and other miRNAs w/seed CUCCCAA)	mature microRNA			4.2E-02	CSF1R
APOH	transporter			4.2E-02	F2
HLA-G	other			4.2E-02	LILRB2
LPA	other			4.2E-02	GHR
NUMBL	other			4.2E-02	DLL4
RASGRF1	other			4.2E-02	KRAS
KRIT1	other			4.2E-02	DLL4
GREM1	other			4.2E-02	GDNF
PMCH	other			4.2E-02	LEP
BEX2	other			4.2E-02	SPP1
Col17a1	other			4.2E-02	SERPINA1
HNRNPAB	enzyme			4.2E-02	SPP1
selegiline	chemical drug			4.2E-02	GDNF

aminoglutethimide	chemical drug	4.2E-02	SPP1	
putrescine	chemical - endogenous mammalian	4.2E-02	TLR2	
puerarin	chemical drug	4.2E-02	LEP	
canrenoate potassium	chemical drug	4.2E-02	SPP1	
GDF2	growth factor	4.2E-02	LYVE1,SPP1	
NR0B2	ligand-dependent nuclear receptor	4.2E-02	CD163,SPP1	
CCL2	cytokine	4.3E-02	IFNAR1,INHBA	
4-phenylbutyric acid	chemical - endogenous mammalian	4.3E-02	AHSG,SERPINA1	
amphetamine	chemical drug	4.3E-02	CSF1R,INHBA	
wortmannin	chemical - kinase inhibitor	4.3E-02	BGLAP,KRAS,LEP	
BMP4	growth factor	4.4E-02	BGLAP,IBSP,NANOG	
let-7	microRNA	4.4E-02	KRAS,NANOG,PLAUR	
EDN1	cytokine	4.4E-02	INHBA,LEP,PLAUR	
TAF4	transcription regulator	4.4E-02	ABL2,SPP1	
RHOA	enzyme	4.4E-02	HSPD1,IBSP	
digoxin	chemical drug	4.5E-02	TNFRSF10A	
desoxycorticosterone	chemical - endogenous mammalian	4.5E-02	SPP1	
LILRA2	other	4.5E-02	MRC1	
IgG2a	complex	4.5E-02	LTA	
Ppp2c	group	4.5E-02	PIM1	
MIRLET7	group	4.5E-02	KRAS	
mir-127	microRNA	4.5E-02	MRC1	
HIF3A	transcription regulator	4.5E-02	PLAUR	
CSF2RA	transmembrane receptor	4.5E-02	PIM1	
HBP1	transcription regulator	4.5E-02	PIM1	
DOCK2	other	4.5E-02	LEP	
TEF	transcription regulator	4.5E-02	PDXK	
PIK3CB	kinase	4.5E-02	DLL4	
ACE	peptidase	4.5E-02	MDK	
DOK1	kinase	4.5E-02	LEP	
ATXN3	peptidase	4.5E-02	IL1RL1	
VEGFD	growth factor	4.5E-02	BGLAP	
RGS16	enzyme	4.5E-02	IL17B	
PTGER3	G-protein coupled receptor	4.5E-02	LEP	
DUSP14	phosphatase	4.5E-02	INHBA	
ZIC3	transcription regulator	4.5E-02	NANOG	
HPSE	enzyme	4.5E-02	PLAUR	

biotin	chemical - endogenous mammalian		4.5E-02	GDNF
alpha-ketoisocaproic acid	chemical - endogenous mammalian		4.5E-02	NADPH
riluzole	chemical drug		4.5E-02	GDNF
rhodioloside	chemical - endogenous non-mammalian		4.5E-02	BGLAP
terbutaline	chemical drug		4.5E-02	LEP
gambogic acid	chemical - endogenous non-mammalian		4.5E-02	TYMS
Ifnar	group		4.5E-02	SPP1,TLR2
SLC13A1	transporter		4.5E-02	CD163,SPP1
FOLR1	transporter		4.5E-02	MDK,TYMS
TBK1	kinase		4.5E-02	INHBA,TLR2
EHF	transcriptio n regulator		4.5E-02	PLAUR,S100A7
CG	complex	-1.697	4.5E-02	IL1R2,INHBA,PLAUR,SPP1
PPARGC1A	transcriptio n regulator	0	4.6E-02	FABP3,IL1R2,INHBA,MB
benzo(a)pyren e	chemical toxicant		4.6E-02	ENTPD5,INHBA,SERPINA1
SMAD2	transcriptio n regulator		4.6E-02	BGLAP,NANOG
NLRP3	other		4.6E-02	MRC1,TYMS
AG490	chemical - kinase inhibitor		4.6E-02	PIM1,SPP1
PC-SPES	chemical drug		4.8E-02	IMPDH1,TYMS
Mmp	group		4.8E-02	CSF1R
Atrial Natriuretic Peptide	group		4.8E-02	LEP
CTSG	peptidase		4.8E-02	CXCL5
PIAS4	transcriptio n regulator		4.8E-02	ALB
PTPRE	phosphatase		4.8E-02	INHBA
NPY2R	G-protein coupled receptor		4.8E-02	LEP
ACVR1B	kinase		4.8E-02	NANOG
PTGER1	G-protein coupled receptor		4.8E-02	GDNF
DMH1	chemical reagent		4.8E-02	MRC1
SOCS2	other		4.8E-02	GHR
N-(3-oxododecanoyl)-homoserine lactone	chemical reagent		4.8E-02	TYMS
picryl chloride	chemical toxicant		4.8E-02	SPP1
hyperforin	chemical drug		4.8E-02	SPP1

maxacalcitol	chemical drug		4.8E-02	ALB
LDLR	transporter		4.8E-02	LRP1,SPP1,WFIKKN1
mir-210	microRNA		4.9E-02	FCER2,PDXK
CpG ODN 2006	chemical reagent		4.9E-02	FCER2,IFNA7
isobutylmethyl xanthine	chemical toxicant		4.9E-02	BGLAP,IBSP,INHBA
Akt	group	-1.091	4.9E-02	DLL4,LEP,SPP1,TLR2
FOXA2	transcription regulator		4.9E-02	ALB,PIM1,SERPINA1
RBL2	other		5.0E-02	PIM1,TYMS
Vegf	group	-1.955	1.1E-01	DLL4,INHBA,LYVE1,PLAUR

Table S7. Canonical pathways for protein co-expression analysis.

Ingenuity Canonical Pathways	*p*-value	Molecules
Role of Osteoblasts, Osteoclasts and Chondrocytes in Rheumatoid Arthritis	1.3E-15	MAP2K4,MAPK1,IL1RL1,ACP5,IL6,BCL2,IL18R1,IL1R2,JUN,WIF1,TGFB1,PIK3CG,BIRC3,IL1RAP,BMP1,IFNG,SRC,TNFSF11,SPP1,GRB2,MAPK8,DKKL1,IL1R1,TNFRSF11A,CSF1R,BMP10,MAPK14,IL1B,BMP7,SFRP1,SOST,LRP1
Acute Phase Response Signaling	2E-15	MAP2K4,HAMP,MAPK1,GRB2,MAPK8,SERPINF1,PDPK1,KRAS,IL6,STAT3,IL1R1,F2,SERPINF2,C1R,SHC1,ALB,APOA1,SOD2,JUN,MAPK14,APCS,PIK3CG,IL1B,SERPINA1,MAP2K1,IL1RAP,RBP4
PDGF Signaling	6.3E-15	MAP2K4,SRC,MAPK1,GRB2,SPHK2,MAPK8,ABL1,ACP1,CRK,KRAS,STAT3,PDGFC,SHC1,JUN,PIK3CG,PDGFRA,SPHK1,RASA1,MAP2K1,PRKCB,PRKCA
PPARα/RXRα Activation	7.9E-15	MAP2K4,ATP,GPD1,MAPK1,MED1,IL1RL1,GRB2,CD36,SMAD3,MAPK8,KRAS,IL6,IL1R1,AIP,IL1R2,SHC1,APOA1,JUN,MAPK14,GHR,TGFB1,PRKACA,IL1B,IL1RAP,MAP2K1,PRKCA,PRKCB
Axonal Guidance Signaling	1.3E-12	MAPK1,SEMA6A,ABL1,SEMA6B,CRK,KRAS,NCK1,PDGFC,SHC1,EFNB2,PIK3CG,EFNA5,NTRK1,ERBB2,RASA1,MAP2K1,RTN4R,BMP1,PRKCA,EPHB4,PLXNC1,PAK6,ADAMTS1,GRB2,EPHA1,EFNA3,MMP10,RAC3,BMP10,SEMA3A,NTRK3,PRKCD,PRKACA,EPHA5,BMP7,EPHA2,MMP9,NRP1,PRKCB
Glucocorticoid Receptor Signaling	4E-12	MAP2K4,MAPK1,SMAD3,POMC,KRAS,IL6,CD163,BCL2,HMGB1,IL1R2,SHC1,JUN,CCL13,TGFB1,IL2,PIK3CG,MAP2K1,IFNG,VCAM1,IL3,GRB2,MED1,MAPK8,STAT3,IL13,HSPA8,BCL2L1,MAPK14,PRKACA,IL1B,KRT18,ESR1,UBE2I
STAT3 Pathway	5E-12	MAP2K4,SRC,MAPK1,MAPK8,KRAS,STAT3,TNFRSF11A,BCL2,GHR,MAPK14,NTRK3,PIM1,TGFB1,NTRK1,PDGFRA,IL10RB,IL1B,MAP2K1,IFNAR1
Role of Macrophages, Fibroblasts and Endothelial Cells in Rheumatoid Arthritis	6.3E-12	MAP2K4,MAPK1,IL1RL1,KRAS,IL6,IL17RA,PDGFC,IL18R1,IL1R2,JUN,WIF1,TGFB1,PIK3CG,MAPKAPK2,MAP2K1,IL1RAP,PRKCA,SRC,VCAM1,TNFSF11,GRB2,DKKL1,STAT3,IL1R1,TLR2,MAPK14,PRKCD,IL1B,SFRP1,SOST,LRP1,PRKCB
HMGB1 Signaling	1E-11	MAP2K4,IFNG,IL3,VCAM1,MAPK1,GRB2,MAPK8,IFNGR1,KRAS,IL1R1,IL6,IL13,IL25,HMGB1,JUN,MAPK14,IL2,TGFB1,PIK3CG,IL1B,MAP2K1
IL-12 Signaling and Production in Macrophages	1.3E-11	MAP2K4,APOE,IFNG,MAPK1,GRB2,MAPK8,IFNGR1,TLR2,ALB,APOA1,JUN,MAPK14,TGFB1,PIK3CG,PRKCD,SERPINA1,MST1R,MAP2K1,PRKCB,RBP4,PRKCA
Colorectal Cancer Metastasis Signaling	1.3E-11	MAP2K4,ATP,GRK2,MAPK1,MMP16,SMAD3,KRAS,IL6,PDGFC,JUN,TGFB1,PIK3CG,MAP2K1,TP53,IFNG,SRC,CASP3,GRB2,MAPK8,MMP10,IFNGR1,STAT3,BIRC5,TLR2,BCL2L1,PRKACA,LRP1,MMP9
ErbB Signaling	1.6E-11	MAP2K4,MAPK1,PAK6,GRB2,MAPK8,NRG4,PDPK1,KRAS,NCK1,SHC1,JUN,MAPK14,PIK3CG,PRKCD,ERBB2,MAP2K1,PRKCB,PRKCA

Molecular Mechanisms of Cancer	2.5E-11	MAP2K4,ATP,MAPK1,SMAD3,ABL1,CRK,KRAS,BCL2,DIABLO,SHC1,RB1,JUN,TGFB1,PIK3CG,RASA1,BIRC3,MAP2K1,BMP1,PRKCA,TP53,SRC,CASP3,PAK6,GRB2,MAPK8,MDM2,RAC3,BMP10,BCL2L1,MAPK14,PRKCD,PRKACA,BMP7,LRP1,PRKCB
Pancreatic Adenocarcinoma Signaling	2.5E-11	TP53,MAP2K4,MAPK1,GRB2,SMAD3,MAPK8,ABL1,MDM2,KRAS,STAT3,BIRC5,PDGFC,BCL2,RB1,BCL2L1,TGFB1,PIK3CG,ERBB2,MAP2K1,MMP9
Ephrin Receptor Signaling	4E-11	SRC,EPHB4,MAPK1,PAK6,GRB2,EPHA1,EFNA3,ABL1,ACP1,CRK,KRAS,NCK1,STAT3,RAC3,PDGFC,SHC1,EFNB2,EFNA5,PIK3CG,EPHA5,RASA1,EPHA2,MAP2K1
Th1 and Th2 Activation Pathway	4E-11	IFNG,IL3,TNFSF11,JAG2,GRB2,IL1RL1,HAVCR2,IFNGR1,CRLF2,STAT3,IL6,IL13,IL25,IL18R1,NOTCH2,JUN,CD80,IL2,TGFB1,PIK3CG,IL10RB,CD274,DLL4,IFNAR1
LXR/RXR Activation	7.9E-11	APOE,MSR1,IL1RL1,CD36,VTN,SERPINF1,IL6,IL1R1,SERPINF2,IL1R2,ALB,APOA1,IL1B,SERPINA1,IL1RAP,MMP9,CCL7,RBP4
Bladder Cancer Signaling	1E-10	TP53,MAPK1,MMP16,FGF9,ABL1,MMP10,MDM2,KRAS,RPS6KA5,PDGFC,RB1,FGF23,ERBB2,FGF7,MAP2K1,MMP9
Role of Tissue Factor in Cancer	1E-10	TP53,SRC,CASP3,MAPK1,GRB2,RPS6KA3,PLAUR,KRAS,RPS6KA5,F2,BCL2L1,F10,YES1,MAPK14,PIK3CG,LYN,IL1B,CXCL1,PRKCA
Osteoarthritis Pathway	1.4E-10	SPP1,LEP,ATP,CASP3,IL1RL1,SMAD3,MMP10,GDF5,ANXA2,IL1R1,PDGFC,TLR2,HMGB1,IL1R2,S100A9,TGFB1,GDF2,SPHK1,IL1B,NAMPT,IL1RAP,LRP1,MMP9,RBP4
Hepatic Cholestasis	1.8E-10	MAP2K4,IFNG,IL3,ATP,IL1RL1,MAPK8,IL1R1,IL6,IL13,IL25,IL1R2,JUN,IL2,TGFB1,PRKCD,PRKACA,IL1B,ESR1,IL1RAP,PRKCB,PRKCA
Neuroinflammation Signaling Pathway	2.2E-10	MAP2K4,ATP,MAPK1,GDNF,IFNB1,IL6,BCL2,HMGB1,JUN,SOD2,TGFB1,PIK3CG,BIRC3,IFNG,VCAM1,CASP3,GRB2,MAPK8,IFNGR1,IL1R1,CSF1R,BIRC5,TLR2,MAPK14,CD80,IL1B,SNCA,CX3CL1,MMP9
UVC-Induced MAPK Signaling	3.1E-10	TP53,MAP2K4,SRC,MAPK14,JUN,MAPK1,PRKCD,MAPK8,KRAS,MAP2K1,PRKCB,PRKCA
UVB-Induced MAPK Signaling	4.6E-10	TP53,MAP2K4,MAPK1,GRB2,MAPK8,RPS6KA3,RPS6KA5,JUN,MAPK14,PIK3CG,PRKCD,MAP2K1,PRKCA,PRKCB
Inhibition of Angiogenesis by TSP1	7.2E-10	TP53,MAP2K4,CD47,MAPK14,JUN,CASP3,MAPK1,TGFB1,CD36,MAPK8,MMP9
Role of Pattern Recognition Receptors in Recognition of Bacteria and Viruses	7.6E-10	MAP2K4,IFNG,IL3,MAPK1,GRB2,IFNB1,MAPK8,IL6,IL13,IL25,TLR2,CLEC7A,IL2,TGFB1,PIK3CG,PRKCD,IL1B,PRKCB,PRKCA
Fc Epsilon RI Signaling	7.8E-10	MAP2K4,IL3,MAPK1,GRB2,MAPK8,PDPK1,KRAS,IL13,RAC3,BTK,MAPK14,GRAP2,PIK3CG,PRKCD,LYN,MAP2K1,PRKCB,PRKCA
Granulocyte Adhesion and Diapedesis	1.6E-09	CSF3R,VCAM1,IL1RL1,MMP16,MMP10,CXCL5,IL1R1,CXCL9,IL1R2,ICAM2,CCL13,CXCL13,PECAM1,IL1B,CXCL1,CCL26,IL1RAP,CX3CL1,CCL19,MMP9,CCL7
TGF-β Signaling	2.1E-09	MAP2K4,MAPK1,GRB2,SMAD3,MAPK8,KRAS,INHBA,BCL2,JUN,MAPK14,PIAS4,AMH,TGFB1,BMP7,MAP2K1
NRF2-mediated Oxidative Stress Response	2.1E-09	MAP2K4,UBB,MAPK1,GRB2,PPIB,PRDX1,MAPK8,KRAS,SOD1,AKR1A1,JUN,MAPK14,SOD2,KEAP1,STIP1,PIK3CG,PRKCD,CAT,MAP2K1,GSTP1,PRKCB,PRKCA
Atherosclerosis Signaling	2.4E-09	PAFAH1B2,APOE,IFNG,VCAM1,MSR1,CD36,CMA1,IL6,PDGFC,TNFRSF14,ALB,APOA1,TGFB1,IL1B,SERPINA1,MMP9,RBP4
IL-6 Signaling	3.5E-09	MAP2K4,MAPK1,GRB2,IL1RL1,MAPK8,KRAS,IL6,STAT3,IL1R1,IL1R2,SHC1,JUN,MAPK14,PIK3CG,IL1B,MAPKAPK2,IL1RAP,MAP2K1
GDNF Family Ligand-Receptor Interactions	4.5E-09	MAP2K4,SHC1,PSPN,JUN,MAPK1,GDNF,GRB2,PIK3CG,GFRA1,MAPK8,KRAS,NCK1,RASA1,MAP2K1
Renin-Angiotensin Signaling	6.5E-09	MAP2K4,ATP,MAPK1,PAK6,GRB2,MAPK8,KRAS,STAT3,SHC1,JUN,MAPK14,PIK3CG,PRKCD,PRKACA,MAP2K1,PRKCB,PRKCA
PPAR Signaling	8.1E-09	MAPK1,IL1RL1,MED1,GRB2,KRAS,IL1R1,PDGFC,AIP,IL1R2,SHC1,JUN,PDGFRA,IL1B,IL1RAP,MAP2K1

IGF-1 Signaling	8.7E-09	IGFBP6,YWHAE,MAPK1,GRB2,YWHAZ,MAPK8,PDPK1,KRAS,STAT3,IGFBP2,SHC1,JUN,PIK3CG,PRKACA,MAP2K1,RASA1
Erythropoietin Signaling	1.3E-08	EPO,SRC,MAPK1,EPOR,GRB2,PDPK1,KRAS,SHC1,JUN,PIK3CG,PRKCD,MAP2K1,PRKCA,PRKCB
IL-10 Signaling	1.4E-08	MAP2K4,MAPK1,IL1RL1,MAPK8,IL6,STAT3,IL1R1,IL1R2,JUN,MAPK14,IL10RB,IL1B,IL1RAP
EGF Signaling	1.4E-08	MAP2K4,SRC,MAPK1,GRB2,MAPK8,STAT3,SHC1,JUN,MAPK14,PIK3CG,MAP2K1,RASA1,PRKCA
Chemokine Signaling	1.4E-08	SRC,CCL13,MAPK14,JUN,MAPK1,CAMK1D,PIK3CG,MAPK8,KRAS,MAP2K1,PRKCB,PRKCA,CCL7
Cholecystokinin/Gastrin-mediated Signaling	1.7E-08	MAP2K4,SRC,MAPK1,GRB2,MAPK8,KRAS,SHC1,JUN,MAPK14,PRKCD,SST,IL1B,MAP2K1,PRKCA,PRKCB
BMP signaling pathway	2.4E-08	MAP2K4,FST,MAPK1,GRB2,MAPK8,KRAS,BMP10,JUN,MAPK14,PRKACA,BMP7,MAP2K1,BMP1
IL-17 Signaling	2.5E-08	MAP2K4,MAPK1,GRB2,MAPK8,KRAS,CXCL5,IL6,IL17RA,JUN,MAPK14,PIK3CG,CXCL1,MAPKAPK2,MAP2K1
FGF Signaling	3E-08	MAPK1,GRB2,FGF9,MAPK8,RPS6KA5,CRK,STAT3,MAPK14,PIK3CG,FGF23,MAPKAPK2,FGF7,MAP2K1,PRKCA
Glioma Signaling	3E-08	TP53,MAPK1,CAMK1D,GRB2,ABL1,MDM2,KRAS,PDGFC,SHC1,RB1,PIK3CG,PRKCD,PDGFRA,MAP2K1,PRKCA,PRKCB
PTEN Signaling	3.9E-08	MAPK1,CASP3,GRB2,PDPK1,KRAS,TNFRSF11A,RAC3,BCL2,SHC1,BCL2L1,GHR,NTRK3,PIK3CG,NTRK1,PDGFRA,MAP2K1
Apoptosis Signaling	4.7E-08	TP53,MAP2K4,MAPK1,CASP3,MAPK8,KRAS,BCL2,DIABLO,BCL2L1,HTRA2,SPTAN1,MAP2K1,BIRC3,PRKCA
IL-15 Signaling	5.8E-08	IL15RA,VCAM1,MAPK1,GRB2,KRAS,IL6,STAT3,RAC3,SHC1,BCL2L1,MAPK14,PIK3CG,MAP2K1
Production of Nitric Oxide and Reactive Oxygen Species in Macrophages	5.9E-08	MAP2K4,APOE,IFNG,MAPK1,GRB2,MAPK8,IFNGR1,TLR2,ALB,APOA1,JUN,MAPK14,PIK3CG,PRKCD,CAT,SERPINA1,MAP2K1,PRKCB,RBP4,PRKCA
Thrombopoietin Signaling	6.2E-08	SHC1,THPO,JUN,MAPK1,GRB2,PIK3CG,PRKCD,KRAS,STAT3,MAP2K1,PRKCB,PRKCA
14-3-3-mediated Signaling	6.3E-08	MAP2K4,SRC,MAPK1,YWHAE,GRB2,YWHAZ,MAPK8,KRAS,JUN,PIK3CG,PRKCD,GFAP,MAP2K1,SNCA,PRKCB,PRKCA
Role of IL-17F in Allergic Inflammatory Airway Diseases	8.5E-08	MAPK1,RPS6KA3,IL1B,CXCL1,RPS6KA5,CXCL5,IL6,IL17RA,MAP2K1,CCL7
IL-8 Signaling	8.5E-08	MAP2K4,SRC,ANGPT2,VCAM1,MAPK1,GRB2,MAPK8,KRAS,RAC3,PDGFC,BCL2,BCL2L1,JUN,PIK3CG,PRKCD,CXCL1,MAP2K1,MMP9,PRKCB,PRKCA
Neuroprotective Role of THOP1 in Alzheimer's Disease	9.8E-08	IFNG,ATP,YWHAE,CMA1,CTSG,MASP1,KLK4,C1R,KLK11,KLK7,PRKACA,SST,HTRA2,MMP9
Type I Diabetes Mellitus Signaling	1E-07	MAP2K4,IFNG,CASP3,MAPK1,MAPK8,IFNGR1,HSPD1,IL1R1,BCL2,MAPK14,CD80,IL2,IL1B,IL1RAP,CPE
p70S6K Signaling	1.3E-07	SRC,YWHAE,MAPK1,GRB2,YWHAZ,PDPK1,KRAS,F2,BTK,SHC1,PIK3CG,PRKCD,LYN,MAP2K1,PRKCA,PRKCB
Hepatic Fibrosis / Hepatic Stellate Cell Activation	1.4E-07	IFNG,VCAM1,LEP,IL1RL1,SMAD3,IFNGR1,IL1R1,IL6,CXCL9,PDGFC,BCL2,IL1R2,TGFB1,COL23A1,PDGFRA,IL1B,IL1RAP,MMP9,IFNAR1
Neuregulin Signaling	1.5E-07	SRC,MAPK1,GRB2,NRG4,PDPK1,KRAS,CRK,SHC1,PRKCD,ERBB2,MAP2K1,PRKCA,PRKCB
Melanocyte Development and Pigmentation Signaling	1.5E-07	SRC,ATP,MAPK1,GRB2,RPS6KA3,POMC,KRAS,RPS6KA5,CRK,BCL2,SHC1,PIK3CG,PRKACA,MAP2K1
Role of MAPK Signaling in the Pathogenesis of Influenza	1.8E-07	MAP2K4,PAFAH1B2,IFNG,MAPK14,CASP3,MAPK1,IFNB1,MAPK8,KRAS,MAP2K1,BCL2,PRKCA

Role of IL-17A in Arthritis	1.8E-07	MAP2K4,MAPK14,MAPK1,GRB2,PIK3CG,MAPK8,CXCL1,CXCL5,MAPKAPK2,IL17RA,MAP2K1,CCL7
RAR Activation	1.9E-07	MAP2K4,SRC,MAPK1,MED1,CSK,SMAD3,MAPK8,PDPK1,JUN,MAPK14,TGFB1,PIK3CG,PRKCD,PRKACA,MAPKAPK2,MAP2K1,PRKCB,RBP4,PRKCA
UVA-Induced MAPK Signaling	2.5E-07	TP53,MAP2K4,MAPK1,CASP3,GRB2,MAPK8,RPS6KA3,KRAS,RPS6KA5,BCL2L1,JUN,MAPK14,PIK3CG,PRKCA
Th1 Pathway	2.8E-07	IFNG,TNFSF11,GRB2,HAVCR2,IFNGR1,IL6,STAT3,IL18R1,NOTCH2,CD80,IL2,PIK3CG,IL10RB,CD274,DLL4,IFNAR1
Leukocyte Extravasation Signaling	2.8E-07	MAP2K4,SRC,VCAM1,MAPK1,GRB2,MMP16,MAPK8,JAM2,ABL1,MMP10,CRK,BTK,MAPK14,PIK3CG,PRKCD,FER,PECAM1,MMP9,PRKCB,PRKCA
NGF Signaling	4.4E-07	TP53,MAP2K4,MAPK1,GRB2,MAPK8,RPS6KA3,PDPK1,CRK,KRAS,RPS6KA5,SHC1,PIK3CG,PRKCD,NTRK1,MAP2K1
p53 Signaling	5.2E-07	TP53,MED1,GRB2,TNFRSF10B,MAPK8,MDM2,BIRC5,BCL2,RB1,BCL2L1,JUN,MAPK14,PIK3CG,TNFRSF10A
IL-17A Signaling in Airway Cells	5.4E-07	MAP2K4,MAPK14,MAPK1,GRB2,PIK3CG,MAPK8,CXCL1,CXCL5,IL6,STAT3,IL17RA,MAP2K1
Neurotrophin/TRK Signaling	5.4E-07	MAP2K4,SHC1,JUN,MAPK1,NTRK3,GRB2,PIK3CG,NTRK1,MAPK8,PDPK1,KRAS,MAP2K1
Coagulation System	6.8E-07	F10,SERPINA5,PROS1,PROC,PLAUR,SERPINA1,F2,SERPINF2
Parkinson's Signaling	7.2E-07	MAPK14,CASP3,MAPK1,PARK7,MAPK8,SNCA
ErbB2-ErbB3 Signaling	7.8E-07	SHC1,JUN,MAPK1,GRB2,PIK3CG,NRG4,PDPK1,KRAS,ERBB2,STAT3,MAP2K1
Huntington's Disease Signaling	8.5E-07	TP53,MAP2K4,UBB,CASP3,MAPK1,GRB2,MAPK8,PDPK1,HSPA8,SHC1,BCL2L1,JUN,ATP5F1B,PIK3CG,PRKCD,NTRK1,RPS27A,RASA1,SNCA,PRKCA,PRKCB
PAK Signaling	8.7E-07	MAP2K4,SHC1,CASP3,MAPK1,PAK6,GRB2,PIK3CG,MAPK8,PDGFRA,KRAS,NCK1,PDGFC,MAP2K1
HIF1α Signaling	1E-06	TP53,EPO,MAPK1,GRB2,MMP16,MAPK8,MMP10,MDM2,KRAS,PDGFC,JUN,MAPK14,PIK3CG,MMP9
Role of NFAT in Cardiac Hypertrophy	1E-06	MAP2K4,SRC,ATP,MAPK1,CAMK1D,GRB2,MAPK8,KRAS,IL6,CTF1,SHC1,MAPK14,TGFB1,PIK3CG,PRKCD,PRKACA,MAP2K1,PRKCB,PRKCA
ErbB4 Signaling	1.1E-06	SHC1,MAPK1,GRB2,PIK3CG,PRKCD,NRG4,PDPK1,KRAS,MAP2K1,PRKCB,PRKCA
IL-3 Signaling	1.1E-06	SHC1,IL3,JUN,MAPK1,GRB2,PIK3CG,PRKCD,KRAS,STAT3,MAP2K1,PRKCB,PRKCA
Prolactin Signaling	1.1E-06	SHC1,JUN,MAPK1,GRB2,PIK3CG,PRKCD,PDPK1,KRAS,STAT3,MAP2K1,PRKCB,PRKCA
Human Embryonic Stem Cell Pluripotency	1.2E-06	GRB2,SMAD3,PDPK1,PDGFC,BMP10,INHBA,NANOG,NTRK3,TGFB1,PIK3CG,NTRK1,PDGFRA,SPHK1,BMP7,BMP1
Role of Cytokines in Mediating Communication between Immune Cells	1.2E-06	IFNG,IL3,TGFB1,IL2,IFNB1,IL1B,IL6,IL13,IL25
Myc Mediated Apoptosis Signaling	1.2E-06	TP53,MAP2K4,SHC1,YWHAE,CASP3,GRB2,PIK3CG,YWHAZ,MAPK8,KRAS,BCL2
Crosstalk between Dendritic Cells and Natural Killer Cells	1.4E-06	CD209,IFNG,IL15RA,IL3,CD80,MICB,IL2,IFNB1,KIR3DL2,CD83,IL6,MICA
VEGF Signaling	1.6E-06	SRC,YWHAE,MAPK1,GRB2,KRAS,PDGFC,BCL2,SHC1,BCL2L1,PIK3CG,MAP2K1,PRKCA,PRKCB
IL-22 Signaling	1.7E-06	MAP2K4,MAPK14,MAPK1,MAPK8,IL10RB,IL22RA2,STAT3
LPS-stimulated MAPK Signaling	1.8E-06	MAP2K4,MAPK14,JUN,MAPK1,GRB2,PIK3CG,PRKCD,MAPK8,KRAS,MAP2K1,PRKCB,PRKCA

Altered T Cell and B Cell Signaling in Rheumatoid Arthritis	1.8E-06	TLR2,IFNG,TNFSF11,SPP1,CD80,CXCL13,TGFB1,IL2,IL1B,TNFRSF13B,IL6,TNFRSF13C
Agranulocyte Adhesion and Diapedesis	1.9E-06	VCAM1,MMP16,MMP10,CXCL5,IL1R1,CXCL9,ICAM2,CCL13,CXCL13,PECAM1,IL1B,CXCL1,CCL26,CX3CL1,MMP9,CCL19,CCL7
ERK/MAPK Signaling	1.9E-06	SRC,MAPK1,PAK6,GRB2,YWHAZ,CRK,KRAS,RPS6KA5,STAT3,RAC3,SHC1,PIK3CG,PRKCD,PRKACA,ESR1,MAP2K1,PRKCA,PRKCB
CXCR4 Signaling	2.1E-06	MAP2K4,SRC,ATP,MAPK1,PAK6,GRB2,MAPK8,CRK,KRAS,JUN,PIK3CG,PRKCD,LYN,MAP2K1,PRKCA,PRKCB
GM-CSF Signaling	2.2E-06	SHC1,BCL2L1,MAPK1,GRB2,PIM1,PIK3CG,LYN,KRAS,STAT3,MAP2K1,PRKCB
IL-17A Signaling in Gastric Cells	2.4E-06	MAP2K4,MAPK14,JUN,MAPK1,MAPK8,CXCL1,IL17RA
Chronic Myeloid Leukemia Signaling	2.5E-06	TP53,MAPK1,GRB2,SMAD3,ABL1,KRAS,CRK,MDM2,RB1,BCL2L1,TGFB1,PIK3CG,MAP2K1
G Beta Gamma Signaling	2.5E-06	SRC,ATP,MAPK1,GRB2,PDPK1,KRAS,BTK,SHC1,PIK3CG,PRKCD,PRKACA,PRKCA,PRKCB
NF-κB Signaling	2.6E-06	TNFSF11,GRB2,UBE2N,MAPK8,KRAS,IL1R1,TNFRSF11A,TLR2,IL1R2,GHR,NTRK3,PIK3CG,NTRK1,PRKACA,PDGFRA,IL1B,PRKCB
PI3K/AKT Signaling	2.7E-06	TP53,YWHAE,MAPK1,GRB2,YWHAZ,PDPK1,KRAS,MDM2,BCL2,SHC1,BCL2L1,NANOG,PIK3CG,MAP2K1
Germ Cell-Sertoli Cell Junction Signaling	3.5E-06	MAP2K4,SRC,MAPK1,PAK6,GRB2,MAPK8,PDPK1,KRAS,RAC3,CDH2,MAPK14,TGFB1,KEAP1,PIK3CG,FER,MAP2K1
Iron homeostasis signaling pathway	3.6E-06	EPO,HAMP,ATP,MAPK1,HFE2,IL6,STAT3,SKP1,CD163,BMP10,PDGFRA,BMP7,LRP1,BMP1
Non-Small Cell Lung Cancer Signaling	3.9E-06	TP53,RB1,MAPK1,GRB2,PIK3CG,ABL1,PDPK1,KRAS,ERBB2,MAP2K1,PRKCA
Role of NANOG in Mammalian Embryonic Stem Cell Pluripotency	4.3E-06	TP53,MAPK1,GRB2,KRAS,STAT3,BMP10,LIFR,SHC1,NANOG,PIK3CG,BMP7,MAP2K1,BMP1
CD40 Signaling	4.4E-06	MAP2K4,MAPK14,JUN,MAPK1,GRB2,PIK3CG,MAPK8,STAT3,MAPKAPK2,MAP2K1,FCER2
Clathrin-mediated Endocytosis Signaling	4.8E-06	APOE,SRC,UBB,GRB2,FGF9,MDM2,PDGFC,F2,HSPA8,ALB,APOA1,PIK3CG,FGF23,RPS27A,SERPINA1,FGF7,RBP4
Opioid Signaling Pathway	5E-06	MAP2K4,SRC,ATP,MAPK1,CAMK1D,GRK2,RPS6KA3,POMC,KRAS,RPS6KA5,RAC3,YES1,PIK3CG,PRKCD,LYN,PRKACA,MAP2K1,PRKCB,PRKCA
Renal Cell Carcinoma Signaling	5E-06	UBB,JUN,MAPK1,PAK6,GRB2,TGFB1,PIK3CG,RPS27A,CRK,KRAS,MAP2K1
Leptin Signaling in Obesity	5E-06	ATP,LEP,MAPK1,GRB2,PIK3CG,PDE3A,PRKACA,POMC,STAT3,MAP2K1,GHRL
Thyroid Cancer Signaling	5.8E-06	TP53,SHC1,MAPK1,GDNF,NTRK3,NTRK1,KRAS,MAP2K1
Gα12/13 Signaling	5.8E-06	MAP2K4,BTK,SRC,CDH2,JUN,MAPK1,GRB2,PIK3CG,MAPK8,KRAS,CDH15,RASA1,MAP2K1,F2
Prostate Cancer Signaling	6E-06	TP53,RB1,MAPK1,GRB2,PIK3CG,ABL1,PDPK1,MDM2,KRAS,MAP2K1,GSTP1,BCL2
Agrin Interactions at Neuromuscular Junction	6E-06	MAP2K4,SRC,JUN,MAPK1,PAK6,MAPK8,NRG4,KRAS,ERBB2,RAC3
Growth Hormone Signaling	6.5E-06	GHR,MAPK1,GRB2,PIK3CG,PRKCD,RPS6KA3,PDPK1,RPS6KA5,STAT3,PRKCB,PRKCA
Regulation of IL-2 Expression in Activated and Anergic T Lymphocytes	6.5E-06	MAP2K4,JUN,MAPK1,CD80,GRB2,TGFB1,IL2,SMAD3,MAPK8,KRAS,MAP2K1
Natural Killer Cell Signaling	6.5E-06	SHC1,MAPK1,PAK6,GRB2,PRKCD,PIK3CG,KIR3DL2,KRAS,NCK1,RAC3,MAP2K1,PRKCB,PRKCA

HGF Signaling	6.5E-06	MAP2K4,JUN,MAPK1,GRB2,PRKCD,PIK3CG,MAPK8,KRAS,STAT3,IL6,MAP2K1,PRKCB,PRKCA
p38 MAPK Signaling	6.5E-06	MAP2K4,TP53,IL1R2,MAPK14,MAPKAPK3,TGFB1,IL1RL1,RPS6KA3,IL1B,RPS6KA5,IL1R1,MAPKAPK2,IL1RAP
B Cell Receptor Signaling	6.9E-06	MAP2K4,MAPK1,GRB2,CSK,MAPK8,ABL1,PDPK1,KRAS,BTK,SHC1,BCL2L1,JUN,MAPK14,PIK3CG,LYN,MAP2K1,PRKCB
GNRH Signaling	8.1E-06	MAP2K4,SRC,ATP,MAPK1,PAK6,GRB2,MAPK8,KRAS,JUN,MAPK14,PRKCD,PRKACA,MAP2K1,PRKCA,PRKCB
FLT3 Signaling in Hematopoietic Progenitor Cells	8.1E-06	SHC1,MAPK14,MAPK1,GRB2,PIK3CG,RPS6KA3,PDPK1,KRAS,RPS6KA5,STAT3,MAP2K1
JAK/Stat Signaling	8.1E-06	SHC1,BCL2L1,PIAS4,JUN,MAPK1,GRB2,PIK3CG,KRAS,IL6,STAT3,MAP2K1
Xenobiotic Metabolism Signaling	8.3E-06	MAP2K4,MAPK1,CAMK1D,GRB2,MED1,MAPK8,KRAS,IL6,AIP,MAPK14,CYP3A4,KEAP1,PIK3CG,PRKCD,CAT,IL1B,MAP2K1,GSTP1,PRKCB,PRKCA
FXR/RXR Activation	8.3E-06	MAP2K4,APOE,ALB,APOA1,VTN,FETUB,MAPK8,SERPINF1,IL1B,SERPINA1,SERPINF2,RBP4
Aryl Hydrocarbon Receptor Signaling	9.1E-06	TP53,SRC,MAPK1,MED1,MAPK8,MDM2,IL6,AIP,RB1,JUN,TGFB1,IL1B,ESR1,GSTP1
Ovarian Cancer Signaling	9.1E-06	TP53,SRC,MAPK1,GRB2,FGF9,ABL1,KRAS,PDGFC,BCL2,RB1,PIK3CG,PRKACA,MAP2K1,MMP9
Regulation of the Epithelial-Mesenchymal Transition Pathway	1E-05	MAP2K4,JAG2,MAPK1,GRB2,FGF9,SMAD3,KRAS,STAT3,CDH2,NOTCH2,TGFB1,PIK3CG,FGF23,FGF7,MAP2K1,MMP9
VDR/RXR Activation	1E-05	IGFBP6,IFNG,TNFSF11,SPP1,MED1,IL2,IL1RL1,PRKCD,PRKCB,PRKCA
RANK Signaling in Osteoclasts	1.1E-05	MAP2K4,SRC,TNFSF11,MAPK14,JUN,MAPK1,GRB2,PIK3CG,MAPK8,TNFRSF11A,BIRC3,MAP2K1
PEDF Signaling	1.2E-05	TP53,BCL2L1,MAPK14,SOD2,MAPK1,GDNF,GRB2,PIK3CG,SERPINF1,KRAS,BCL2
T Helper Cell Differentiation	1.2E-05	IFNG,CD80,TGFB1,IL2,IL10RB,IFNGR1,IL6,STAT3,IL13,IL18R1
Tec Kinase Signaling	1.4E-05	MAP2K4,SRC,PAK6,GRB2,TNFRSF10B,MAPK8,STAT3,BTK,YES1,PIK3CG,PRKCD,LYN,TNFRSF10A,PRKCA,PRKCB
4-1BB Signaling in T Lymphocytes	1.4E-05	MAP2K4,MAPK14,JUN,MAPK1,TNFSF9,MAPK8,MAP2K1
CNTF Signaling	1.4E-05	LIFR,MAPK1,GRB2,PIK3CG,RPS6KA3,KRAS,RPS6KA5,STAT3,MAP2K1
Communication between Innate and Adaptive Immune Cells	1.6E-05	TLR2,IFNG,IL3,CD80,IL2,IFNB1,IL1B,TNFRSF13B,CD83,IL6,TNFRSF13C
Toll-like Receptor Signaling	1.7E-05	TLR2,MAP2K4,UBB,MAPK14,JUN,MAPK1,IL1RL1,MAPK8,RPS27A,IL1B
Intrinsic Prothrombin Activation Pathway	1.8E-05	KLK11,F10,KLK7,PROS1,PROC,F2,KLK4
Paxillin Signaling	1.9E-05	MAP2K4,SRC,MAPK14,MAPK1,PAK6,GRB2,PIK3CG,CSK,MAPK8,CRK,KRAS,NCK1
Pyridoxal 5'-phosphate Salvage Pathway	1.9E-05	PDXK,MAP2K4,ATP,MAPK1,PIM1,PRKCD,MAPK8,PLK1,MAP2K1
Salvage Pathways of Pyrimidine Ribonucleotides	2E-05	MAP2K4,AK1,ATP,MAPK1,PIM1,PRKCD,NME2,MAPK8,PLK1,CMPK1,MAP2K1
T Cell Receptor Signaling	2.3E-05	BTK,MAP2K4,JUN,MAPK1,GRB2,GRAP2,PIK3CG,CSK,MAPK8,KRAS,MAP2K1,RASA1
Th2 Pathway	2.5E-05	IFNG,NOTCH2,IL3,JAG2,JUN,CD80,GRB2,TGFB1,IL2,IL1RL1,PIK3CG,CRLF2,IL13,IL25
Ceramide Signaling	2.5E-05	DIABLO,MAP2K4,JUN,GRB2,PIK3CG,SPHK2,MAPK8,SPHK1,KRAS,MAP2K1,BCL2

Death Receptor Signaling	2.5E-05	DIABLO,MAP2K4,CASP3,TNFRSF10B,MAPK8,HTRA2,TNFSF15,SPTAN1,BIRC3,TNFRSF10A,BCL2
Macropinocytosis Signaling	2.5E-05	MRC1,SRC,GRB2,PIK3CG,PRKCD,KRAS,PDGFC,CSF1R,PRKCB,PRKCA
Induction of Apoptosis by HIV1	2.5E-05	DIABLO,TP53,MAP2K4,BCL2L1,CASP3,MAPK8,HTRA2,BIRC3,BCL2
IL-17A Signaling in Fibroblasts	2.6E-05	MAPK14,JUN,MAPK1,CXCL5,IL6,IL17RA,CCL7
Fcγ Receptor-mediated Phagocytosis in Macrophages and Monocytes	2.8E-05	SRC,YES1,MAPK1,PIK3CG,PRKCD,LYN,CRK,NCK1,RAC3,PRKCB,PRKCA
Cell Cycle: G2/M DNA Damage Checkpoint Regulation	2.9E-05	TP53,YWHAE,YWHAZ,ABL1,PLK1,MDM2,SKP1,CCNB1
mTOR Signaling	3E-05	MAPK1,GRB2,RPS6KA3,PDPK1,KRAS,RPS6KA5,PDGFC,RPS7,EIF4G2,EIF4A3,PIK3CG,PRKCD,RPS27A,PRKCB,RPSA,PRKCA
IL-2 Signaling	3.3E-05	SHC1,JUN,MAPK1,GRB2,IL2,PIK3CG,MAPK8,KRAS,MAP2K1
Role of JAK family kinases in IL-6-type Cytokine Signaling	3.4E-05	MAP2K4,MAPK14,MAPK1,MAPK8,IL6,STAT3
Docosahexaenoic Acid (DHA) Signaling	3.9E-05	BCL2L1,CASP3,GRB2,PIK3CG,SERPINF1,IL1B,PDPK1,BCL2
G-Protein Coupled Receptor Signaling	4.2E-05	SMPDL3A,SRC,PDE2A,ATP,MAPK1,GRK2,GRB2,PDE3A,PDPK1,KRAS,STAT3,SHC1,PIK3CG,PRKACA,PDE5A,RASA1,MAP2K1,PRKCB,PRKCA
Antiproliferative Role of TOB in T Cell Signaling	4.3E-05	RB1,MAPK1,TGFB1,IL2,SMAD3,SKP1
Lymphotoxin β Receptor Signaling	4.8E-05	DIABLO,BCL2L1,VCAM1,CASP3,MAPK1,GRB2,PIK3CG,PDPK1,CXCL1
Endothelin-1 Signaling	5.1E-05	PAFAH1B2,SRC,ATP,CASP3,MAPK1,GRB2,MAPK8,KRAS,SHC1,JUN,MAPK14,PIK3CG,PRKCD,PRKCA,PRKCB
MSP-RON Signaling Pathway	5.4E-05	TLR2,KLK11,IFNG,KLK7,IL3,GRB2,PIK3CG,MST1R,KLK4
VEGF Family Ligand-Receptor Interactions	6E-05	SHC1,MAPK1,GRB2,PIK3CG,PRKCD,KRAS,MAP2K1,PRKCB,NRP1,PRKCA
Role of JAK1 and JAK3 in γc Cytokine Signaling	6.9E-05	SHC1,IL15RA,MAPK1,GRB2,IL2,PIK3CG,KRAS,CRLF2,STAT3
Melanoma Signaling	6.9E-05	TP53,RB1,MAPK1,GRB2,PIK3CG,MDM2,KRAS,MAP2K1
Differential Regulation of Cytokine Production in Macrophages and T Helper Cells by IL-17A and IL-17F	7.4E-05	IL3,IL1B,CXCL1,IL6,IL13
TREM1 Signaling	7.8E-05	TLR2,MAPK1,GRB2,IL1RL1,IL1B,CD83,IL6,STAT3,CCL7
B Cell Activating Factor Signaling	7.8E-05	MAP2K4,MAPK14,JUN,MAPK1,MAPK8,TNFRSF13B,TNFRSF13C
Amyotrophic Lateral Sclerosis Signaling	7.9E-05	TP53,BCL2L1,CASP3,GDNF,GRB2,PIK3CG,CAT,SOD1,PDGFC,BIRC3,BCL2
IL-7 Signaling Pathway	8.1E-05	SHC1,IFNG,MAPK14,JUN,MAPK1,GRB2,PIK3CG,LYN,PDPK1,BCL2
Telomerase Signaling	8.5E-05	TP53,SHC1,RB1,MAPK1,GRB2,IL2,PIK3CG,ABL1,PDPK1,KRAS,MAP2K1
Reelin Signaling in Neurons	9.1E-05	MAP2K4,PAFAH1B2,APOE,SRC,YES1,GRB2,PIK3CG,LYN,MAPK8,LRP8
Nitric Oxide Signaling in the Cardiovascular System	9.3E-05	PDE2A,MAPK1,GRB2,PIK3CG,PRKCD,PRKACA,PDE5A,PDGFC,MAP2K1,PRKCB,PRKCA
FcγRIIB Signaling in B Lymphocytes	9.5E-05	BTK,MAP2K4,SHC1,GRB2,PIK3CG,LYN,MAPK8,PDPK1,KRAS
Angiopoietin Signaling	9.5E-05	ANGPT2,PAK6,GRB2,PIK3CG,CRK,KRAS,NCK1,RASA1,BIRC5

Acute Myeloid Leukemia Signaling	0.0001	CSF3R,MAP2K4,MAPK1,GRB2,PIM1,PIK3CG,KRAS,STAT3,CSF1R,MAP2K1
Sertoli Cell-Sertoli Cell Junction Signaling	0.00011	MAP2K4,EPB41,SRC,ATP,MAPK14,JUN,MAPK1,KEAP1,PRKACA,MAPK8,JAM2,KRAS,SPTAN1,MAP2K1
Regulation of eIF4 and p70S6K Signaling	0.00014	RPS7,SHC1,MAPK14,MAPK1,EIF4G2,GRB2,EIF4A3,PIK3CG,RPS27A,PDPK1,KRAS,MAP2K1,RPSA
Glioblastoma Multiforme Signaling	0.00014	TP53,SRC,MAPK1,GRB2,KRAS,MDM2,PDGFC,SHC1,RB1,PIK3CG,PRKCD,PDGFRA,MAP2K1
FAK Signaling	0.00016	SRC,MAPK1,PAK6,GRB2,PIK3CG,CSK,PDPK1,CRK,KRAS,MAP2K1
Role of IL-17A in Psoriasis	0.00019	S100A9,CXCL1,CXCL5,IL17RA
Hematopoiesis from Multipotent Stem Cells	0.00019	EPO,THPO,IL3,IL2
Sumoylation Pathway	0.00019	TP53,MAP2K4,ATP,PIAS4,JUN,MAPK8,GDI2,MDM2,SNCA,UBE2I
Factors Promoting Cardiogenesis in Vertebrates	0.0002	MAPK14,TGFB1,PRKCD,BMP7,LRP1,BMP10,PRKCB,BMP1,PRKCA
Adrenomedullin signaling pathway	0.0002	MAP2K4,MAPK1,CASP3,GRB2,CSK,MAPK8,KRAS,BCL2,SHC1,MAPK14,PIK3CG,PRKACA,IL1B,MAP2K1
Insulin Receptor Signaling	0.0002	SHC1,ATP,MAPK1,GRB2,PIK3CG,PRKACA,MAPK8,PDPK1,CRK,KRAS,NCK1,MAP2K1
Endometrial Cancer Signaling	0.00021	TP53,MAPK1,GRB2,PIK3CG,PDPK1,KRAS,ERBB2,MAP2K1
Oncostatin M Signaling	0.00021	SHC1,MAPK1,GRB2,KRAS,STAT3,MAP2K1
Dendritic Cell Maturation	0.00021	MAP2K4,LEP,MAPK1,GRB2,IFNB1,MAPK8,CD83,IL6,TLR2,MAPK14,CD80,PIK3CG,IL1B,IFNAR1
CCR3 Signaling in Eosinophils	0.00023	MAPK14,MAPK1,PAK6,GRB2,PIK3CG,PRKCD,KRAS,CCL26,MAP2K1,PRKCB,PRKCA
ERK5 Signaling	0.00023	CTF1,SRC,YWHAE,NTRK1,YWHAZ,RPS6KA3,KRAS,RPS6KA5
Integrin Signaling	0.00024	MAP2K4,SRC,PAK6,MAPK1,GRB2,MAPK8,ABL1,KRAS,CRK,NCK1,BCAR3,RAC3,SHC1,PIK3CG,MAP2K1
Cancer Drug Resistance By Drug Efflux	0.00025	TP53,ATP,MAPK1,PIK3CG,MDM2,KRAS,MAP2K1
Differential Regulation of Cytokine Production in Intestinal Epithelial Cells by IL-17A and IL-17F	0.00026	IFNG,IL3,IL1B,CXCL1,IL13
Role of JAK1, JAK2 and TYK2 in Interferon Signaling	0.00026	IFNG,IFNB1,IFNGR1,STAT3,IFNAR1
Mouse Embryonic Stem Cell Pluripotency	0.00026	TP53,LIFR,MAPK14,NANOG,MAPK1,GRB2,PIK3CG,KRAS,STAT3,MAP2K1
Phagosome Formation	0.00026	TLR2,MRC1,CLEC7A,MSR1,GRB2,PIK3CG,VTN,PRKCD,PRKCB,PRKCA,FCER2
HER-2 Signaling in Breast Cancer	0.00026	TP53,GRB2,PIK3CG,PRKCD,MDM2,KRAS,ERBB2,PRKCB,PRKCA
SAPK/JNK Signaling	0.00029	TP53,MAP2K4,SHC1,JUN,GRB2,PIK3CG,MAPK8,CRK,KRAS,RAC3
Actin Cytoskeleton Signaling	0.00032	PAK6,MAPK1,GRB2,FGF9,CSK,KRAS,CRK,PDGFC,RAC3,F2,SHC1,PIK3CG,FGF23,FGF7,MAP2K1
Thrombin Signaling	0.00032	SRC,CAMK1D,MAPK1,GRB2,PDPK1,KRAS,F2,SHC1,MAPK14,PIK3CG,PRKCD,MAP2K1,PRKCA,PRKCB
Role of PI3K/AKT Signaling in the Pathogenesis of Influenza	0.00035	IFNG,MAPK1,GRB2,PIK3CG,IFNB1,CRK,MAP2K1,IFNAR1
Extrinsic Prothrombin Activation Pathway	0.00036	F10,PROS1,PROC,F2
Virus Entry via Endocytic Pathways	0.00039	SRC,CD55,GRB2,PIK3CG,PRKCD,ABL1,KRAS,RAC3,PRKCB,PRKCA

Type II Diabetes Mellitus Signaling	0.00039	MAP2K4,ATP,MAPK1,GRB2,PIK3CG,PRKCD,CD36,PKM,MAPK8,PDPK1,PRKCB,PRKCA
Glycolysis I	0.00039	PGK1,GPI,ATP,PKM,ENO2
Gluconeogenesis I	0.00039	PGK1,GPI,ATP,ENO2,MDH1
P2Y Purigenic Receptor Signaling Pathway	0.00044	ATP,JUN,MAPK1,GRB2,PIK3CG,PRKCD,PRKACA,KRAS,MAP2K1,PRKCB,PRKCA
IL-1 Signaling	0.00045	MAP2K4,ATP,MAPK14,JUN,MAPK1,PRKACA,MAPK8,IL1R1,IL1RAP
Activation of IRF by Cytosolic Pattern Recognition Receptors	0.00046	MAP2K4,JUN,PPIB,IFNB1,MAPK8,IL6,IFNAR1
April Mediated Signaling	0.00046	MAP2K4,MAPK14,JUN,MAPK1,MAPK8,TNFRSF13B
CD28 Signaling in T Helper Cells	0.00049	MAP2K4,JUN,CD80,GRB2,GRAP2,IL2,PIK3CG,CSK,MAPK8,PDPK1,MAP2K1
Relaxin Signaling	0.0005	SMPDL3A,PDE2A,ATP,JUN,MAPK1,GRB2,PIK3CG,PDE3A,PRKACA,PDE5A,MAP2K1,MMP9
Ephrin A Signaling	0.00058	GRB2,PIK3CG,EFNA5,EPHA1,EFNA3,EPHA5,EPHA2
Cardiac Hypertrophy Signaling	0.0006	MAP2K4,ATP,MAPK1,MAPKAPK3,GRB2,MAPK8,KRAS,IL6,JUN,MAPK14,TGFB1,PIK3CG,PRKACA,MAPKAPK2,MAP2K1
Estrogen-Dependent Breast Cancer Signaling	0.00069	SRC,JUN,MAPK1,GRB2,PIK3CG,KRAS,ESR1,HSD17B1
Wnt/β-catenin Signaling	0.00076	TP53,SRC,UBB,CDH2,WIF1,JUN,TGFB1,RPS27A,MDM2,DKKL1,SFRP1,LRP1
ILK Signaling	0.00093	MAP2K4,JUN,CASP3,MAPK1,GRB2,PIK3CG,MUC1,MAPK8,PDPK1,RPS6KA5,KRT18,PDGFC,MMP9
eNOS Signaling	0.00105	HSPA8,ATP,CASP3,GRB2,PIK3CG,PRKCD,PRKACA,PDPK1,PDGFC,ESR1,PRKCB,PRKCA
EIF2 Signaling	0.00107	MAPK1,GRB2,PDPK1,KRAS,BCL2,RPS7,SHC1,EIF4G2,EIF4A3,PIK3CG,EIF5,RPS27A,MAP2K1,RPSA
3-phosphoinositide Biosynthesis	0.00112	SRC,ATP,GRB2,ACP1,ACP5,SET,CA3,CD80,PIK3CG,PDGFRA,ERBB2,ESR1,RASA1
GP6 Signaling Pathway	0.00126	BTK,GRB2,GRAP2,PIK3CG,COL23A1,PRKCD,LYN,PDPK1,PRKCB,PRKCA
TNFR1 Signaling	0.00126	MAP2K4,JUN,CASP3,PAK6,MAPK8,BIRC3
Superoxide Radicals Degradation	0.00129	SOD2,CAT,SOD1
Breast Cancer Regulation by Stathmin1	0.00132	TP53,SHC1,ATP,MAPK1,CAMK1D,GRB2,PRKCD,PIK3CG,PRKACA,KRAS,MAP2K1,PRKCB,PRKCA
PI3K Signaling in B Lymphocytes	0.00145	BTK,JUN,MAPK1,PIK3CG,LYN,ABL1,PDPK1,KRAS,MAP2K1,PRKCB
α-Adrenergic Signaling	0.00148	ATP,MAPK1,PRKCD,PRKACA,KRAS,MAP2K1,PRKCB,PRKCA
Role of JAK2 in Hormone-like Cytokine Signaling	0.00148	EPO,SHC1,GHR,EPOR,STAT3
Glioma Invasiveness Signaling	0.00155	MAPK1,GRB2,PIK3CG,VTN,PLAUR,KRAS,MMP9
Cell Cycle: G1/S Checkpoint Regulation	0.00155	TP53,RB1,TGFB1,SMAD3,ABL1,MDM2,SKP1
NF-κB Activation by Viruses	0.00158	MAPK1,GRB2,PIK3CG,PRKCD,KRAS,TNFRSF14,PRKCB,PRKCA
CCR5 Signaling in Macrophages	0.00158	MAP2K4,MAPK14,JUN,MAPK1,PRKCD,MAPK8,PRKCB,PRKCA
Neuropathic Pain Signaling In Dorsal Horn Neurons	0.00162	SRC,CAMK1D,MAPK1,GRB2,PIK3CG,PRKCD,PRKACA,PRKCB,PRKCA
PKCθ Signaling in T Lymphocytes	0.00174	MAP2K4,JUN,MAPK1,CD80,GRB2,GRAP2,IL2,PIK3CG,MAPK8,KRAS,RAC3
Phospholipase C Signaling	0.00182	PEBP1,SRC,MAPK1,GRB2,RPS6KA3,KRAS,BTK,SHC1,GRAP2,PRKCD,LYN,MAP2K1,PRKCA,PRKCB

Melatonin Signaling	0.00186	MAP2K4,MAPK1,PRKCD,PRKACA,MAP2K1,PRKCB,PRKCA
Interferon Signaling	0.00195	IFNG,IFNB1,IFNGR1,IFNAR1,BCL2
Corticotropin Releasing Hormone Signaling	0.00204	ATP,MAPK14,JUN,MAPK1,PRKCD,PRKACA,POMC,MAP2K1,PRKCB,PRKCA
Rac Signaling	0.00224	MAP2K4,JUN,MAPK1,PAK6,GRB2,PIK3CG,MAPK8,KRAS,MAP2K1
CD27 Signaling in Lymphocytes	0.0024	MAP2K4,BCL2L1,JUN,CASP3,MAPK8,MAP2K1
NAD Phosphorylation and Dephosphorylation	0.00245	ATP,ACP1,ACP5
Aldosterone Signaling in Epithelial Cells	0.00263	HSPA8,MAPK1,GRB2,PIK3CG,PRKCD,PDPK1,KRAS,HSPD1,MAP2K1,PRKCB,PRKCA
Retinoic acid Mediated Apoptosis Signaling	0.00263	IFNG,CASP3,TNFRSF10B,IFNB1,TNFRSF10A,IFNAR1
ATM Signaling	0.00263	TP53,MAP2K4,MAPK14,JUN,MAPK8,ABL1,MDM2,CCNB1
Pyrimidine Deoxyribonucleotides De Novo Biosynthesis I	0.00263	AK1,ATP,NME2,CMPK1
Antiproliferative Role of Somatostatin Receptor 2	0.00275	SRC,MAPK1,GRB2,PIK3CG,SST,KRAS,MAP2K1
Systemic Lupus Erythematosus Signaling	0.00295	JUN,MAPK1,CD80,GRB2,IL2,PIK3CG,HNRNPA2B1,LYN,IL1B,KRAS,IL6,TNFRSF13C
LPS/IL-1 Mediated Inhibition of RXR Function	0.00339	IL1R2,MAP2K4,APOE,JUN,CYP3A4,IL1RL1,CAT,MAPK8,IL1B,IL1R1,IL1RAP,GSTP1
CDK5 Signaling	0.00347	ATP,MAPK14,MAPK1,PRKACA,MAPK8,ABL1,KRAS,MAP2K1
tRNA Splicing	0.00355	SMPDL3A,PDE2A,ATP,PDE3A,PDE5A
Mitochondrial Dysfunction	0.00363	MAP2K4,ATP,SOD2,ATP5F1B,CASP3,PARK7,CAT,MAPK8,HTRA2,SNCA,BCL2
Role of PKR in Interferon Induction and Antiviral Response	0.00398	TP53,IFNG,MAPK14,CASP3,IFNB1
Role of p14/p19ARF in Tumor Suppression	0.00398	TP53,RB1,GRB2,PIK3CG,MDM2
Superpathway of Inositol Phosphate Compounds	0.00407	SRC,ATP,GRB2,ACP1,ACP5,SET,CA3,CD80,PIK3CG,PDGFRA,ERBB2,ESR1,RASA1
MIF Regulation of Innate Immunity	0.00437	TP53,MAP2K4,JUN,MAPK1,MAPK8
Sirtuin Signaling Pathway	0.00447	TP53,EPO,PGK1,PPID,HIST1H1C,ATP,PPIF,MAPK1,SOD1,STAT3,JUN,SOD2,ATP5F1B,XRCC6,NAMPT
Role of Lipids/Lipid Rafts in the Pathogenesis of Influenza	0.00501	IFNG,IFNB1,IFNAR1
Graft-versus-Host Disease Signaling	0.00537	IFNG,CD80,IL2,IL1B,IL6
Androgen Signaling	0.0055	SHC1,SRC,JUN,MAPK1,SMAD3,PRKCD,PRKACA,PRKCB,PRKCA
OX40 Signaling Pathway	0.00562	MAP2K4,BCL2L1,JUN,IL2,MAPK8,BCL2
Protein Kinase A Signaling	0.00575	SMPDL3A,HIST1H1C,PDE2A,ATP,MAPK1,YWHAE,PDE3A,SMAD3,YWHAZ,ACP1,DUSP3,TGFB1,PRKCD,PRKACA,PDE5A,MAP2K1,PRKCB,PRKCA
Small Cell Lung Cancer Signaling	0.00603	TP53,RB1,BCL2L1,GRB2,PIK3CG,ABL1,BCL2
UDP-N-acetyl-D-galactosamine Biosynthesis II	0.00617	GPI,HK2,ATP
Gαq Signaling	0.00631	BTK,MAPK1,GRK2,GRB2,PIK3CG,CSK,PRKCD,MAP2K1,PRKCB,PRKCA
Trehalose Degradation II (Trehalase)	0.00631	HK2,ATP

Gap Junction Signaling	0.00676	SRC,ATP,MAPK1,GRB2,PIK3CG,PRKCD,PRKACA,KRAS,MAP2K1,PRKCB,PRKCA
TNFR2 Signaling	0.00708	MAP2K4,JUN,MAPK8,BIRC3
Complement System	0.00708	C1R,CD55,CD59,MASP1
iNOS Signaling	0.00708	IFNG,MAPK14,JUN,MAPK1,IFNGR1
Sperm Motility	0.00813	PAFAH1B2,PDE2A,ATP,PRKCD,PRKACA,MST1R,PRKCB,PRKCA
Synaptic Long Term Potentiation	0.00871	ATP,MAPK1,PRKCD,PRKACA,KRAS,MAP2K1,PRKCB,PRKCA
Gαi Signaling	0.00871	SHC1,SRC,ATP,MAPK1,GRB2,PRKACA,KRAS,STAT3
Caveolar-mediated Endocytosis Signaling	0.00891	SRC,ALB,CD55,ABL1,DYRK3,PRKCA
Role of Hypercytokinemia/hyperchemokinemia in the Pathogenesis of Influenza	0.00891	IFNG,IFNB1,IL1B,IL6
cAMP-mediated signaling	0.00891	SMPDL3A,SRC,PDE2A,ATP,CAMK1D,MAPK1,GRK2,PDE3A,PRKACA,PDE5A,STAT3,MAP2K1
Ceramide Degradation	0.00933	ATP,ASAH2
Sphingosine-1-phosphate Signaling	0.00955	CASP3,MAPK1,GRB2,PIK3CG,SPHK1,PDGFRA,PDGFC,ASAH2
Hypoxia Signaling in the Cardiovascular System	0.01148	TP53,EPO,JUN,UBE2N,MDM2,UBE2I
iCOS-iCOSL Signaling in T Helper Cells	0.01175	SHC1,CD80,GRB2,GRAP2,IL2,PIK3CG,CSK,PDPK1
CREB Signaling in Neurons	0.0123	SHC1,ATP,MAPK1,GRB2,PIK3CG,PRKCD,PRKACA,KRAS,MAP2K1,PRKCB,PRKCA
Regulation of Cellular Mechanics by Calpain Protease	0.01259	RB1,SRC,MAPK1,GRB2,KRAS
fMLP Signaling in Neutrophils	0.01288	MAPK1,GRB2,PIK3CG,PRKCD,KRAS,MAP2K1,PRKCB,PRKCA
Glycerol Degradation I	0.01288	ATP,GPD1
NAD Biosynthesis III	0.01288	ATP,NAMPT
Inhibition of Matrix Metalloproteases	0.01349	MMP16,MMP10,LRP1,MMP9
Protein Ubiquitination Pathway	0.01585	HSPA8,IFNG,UBB,ATP,UBE2N,RPS27A,MDM2,HSPD1,SKP1,PSMA2,BIRC3,USP25,UBE2I
Estrogen Receptor Signaling	0.01585	SHC1,SRC,MAPK1,GRB2,MED1,KRAS,ESR1,MAP2K1
Cyclins and Cell Cycle Regulation	0.0166	TP53,RB1,TGFB1,ABL1,SKP1,CCNB1
Role of Oct4 in Mammalian Embryonic Stem Cell Pluripotency	0.0195	TP53,RB1,SPP1,NANOG
Epithelial Adherens Junction Signaling	0.02089	SRC,NOTCH2,YES1,CDH2,KEAP1,FER,CRK,KRAS
Hematopoiesis from Pluripotent Stem Cells	0.02089	EPO,IL3,IL2,IL6
Sphingosine and Sphingosine-1-phosphate Metabolism	0.02138	ATP,ASAH2
IL-15 Production	0.02344	IFNB1,IL6,MST1R
NAD Salvage Pathway II	0.02344	ATP,ACP1,ACP5
Hereditary Breast Cancer Signaling	0.02344	TP53,RB1,UBB,GRB2,PIK3CG,RPS27A,KRAS,CCNB1

Pyrimidine Ribonucleotides Interconversion	0.02455	AK1,ATP,NME2,CMPK1
Citrulline Degradation	0.0257	ATP
NAD Biosynthesis from 2-amino-3-carboxymuconate Semialdehyde	0.0263	ATP,ABL1
Phosphatidylcholine Biosynthesis I	0.0263	ATP,CHKB
Semaphorin Signaling in Neurons	0.02884	SEMA3A,MAPK1,PAK6,NRP1
Pyrimidine Ribonucleotides De Novo Biosynthesis	0.0309	AK1,ATP,NME2,CMPK1
Glucose and Glucose-1-phosphate Degradation	0.03162	HK2,ATP
Phosphatidylethanolamine Biosynthesis II	0.03162	ATP,CHKB
Signaling by Rho Family GTPases	0.03388	MAP2K4,CDH2,JUN,MAPK1,PAK6,GRB2,PIK3CG,MAPK8,GFAP,CDH15,MAP2K1
Ephrin B Signaling	0.03467	EFNB2,EPHB4,MAPK1,ACP1,RAC3
Sonic Hedgehog Signaling	0.03467	GRK2,PRKACA,CCNB1
GDP-glucose Biosynthesis	0.03715	HK2,ATP
Role of NFAT in Regulation of the Immune Response	0.0389	BTK,JUN,MAPK1,CD80,GRB2,PIK3CG,LYN,KRAS,MAP2K1
Synaptic Long Term Depression	0.04467	PAFAH1B2,MAPK1,PRKCD,LYN,KRAS,MAP2K1,PRKCB,PRKCA
Cardiomyocyte Differentiation via BMP Receptors	0.04898	BMP7,BMP10
autophagy	0.05129	CTSV,CTSG,CTSE,BCL2
Adenine and Adenosine Salvage VI	0.05129	ATP
Cardiac β-adrenergic Signaling	0.0537	SMPDL3A,PDE2A,ATP,GRK2,PDE3A,PRKACA,PDE5A
Granzyme A Signaling	0.05623	SET,HIST1H1C
Telomere Extension by Telomerase	0.05623	XRCC6,HNRNPA2B1
Cdc42 Signaling	0.05754	MAP2K4,SRC,MAPK14,JUN,MAPK1,MAPK8,RASA1
Colanic Acid Building Blocks Biosynthesis	0.0631	GPI,ATP
Notch Signaling	0.06607	NOTCH2,JAG2,DLL4
IL-4 Signaling	0.06918	SHC1,GRB2,PIK3CG,KRAS,FCER2
Glutaryl-CoA Degradation	0.07079	PARK7,CA1
Bile Acid Biosynthesis, Neutral Pathway	0.07079	ATP,CYP3A4
Acetyl-CoA Biosynthesis III (from Citrate)	0.07586	ATP
Thiamin Salvage III	0.07586	ATP
D-glucuronate Degradation I	0.07586	AKR1A1
Sulfate Activation for Sulfonation	0.07586	ATP
Glycerol-3-phosphate Shuttle	0.07586	GPD1

Lipoate Salvage and Modification	0.07586	ATP
Basal Cell Carcinoma Signaling	0.07586	TP53,BMP7,BMP10,BMP1
GPCR-Mediated Integration of Enteroendocrine Signaling Exemplified by an L Cell	0.07586	ATP,PRKACA,SST,VIP
RAN Signaling	0.07762	KPNB1,KPNA2
NAD biosynthesis II (from tryptophan)	0.07762	ATP,ABL1
Purine Nucleotides De Novo Biosynthesis II	0.07762	ATP,IMPDH2
DNA damage-induced 14-3-3σ Signaling	0.07762	TP53,CCNB1
Mechanisms of Viral Exit from Host Cells	0.07943	PRKCD,PRKCB,PRKCA
Transcriptional Regulatory Network in Embryonic Stem Cells	0.07943	SET,NANOG,STAT3
GADD45 Signaling	0.08511	TP53,CCNB1
TR/RXR Activation	0.0912	F10,GRB2,MED1,PIK3CG,MDM2
GPCR-Mediated Nutrient Sensing in Enteroendocrine Cells	0.0912	ATP,PRKCD,PRKACA,PRKCB,PRKCA
Endoplasmic Reticulum Stress Pathway	0.09333	CASP3,MAPK8
Gustation Pathway	0.09333	SMPDL3A,PDE2A,ATP,PDE3A,PRKACA,PDE5A
AMPK Signaling	0.0955	SRC,AK1,LEP,MAPK14,MAPK1,GRB2,PIK3CG,PRKACA,PDPK1
NADH Repair	0.1	ATP
Glutamine Biosynthesis I	0.1	ATP
Flavin Biosynthesis IV (Mammalian)	0.1	ATP
Proline Biosynthesis I	0.1	ATP
1D-myo-inositol Hexakisphosphate Biosynthesis V (from Ins(1,3,4)P3)	0.1	ATP
Inflammasome pathway	0.10162	ATP,IL1B
Antioxidant Action of Vitamin C	0.10351	MAP2K4,PAFAH1B2,MAPK14,MAPK1,MAPK8
IL-9 Signaling	0.10495	GRB2,PIK3CG,STAT3
Primary Immunodeficiency Signaling	0.10495	BTK,TNFRSF13B,TNFRSF13C
Adipogenesis pathway	0.10914	TP53,RB1,LEP,TGFB1,SMAD3,BMP7
nNOS Signaling in Neurons	0.11588	PRKCD,PRKCB,PRKCA
RhoGDI Signaling	0.11641	SRC,CDH2,PAK6,GDI2,CDH15,ESR1,PRKCA
D-myo-inositol (1,4,5,6)-Tetrakisphosphate Biosynthesis	0.12162	SET,CA3,ATP,ACP1,ACP5,RASA1
D-myo-inositol (3,4,5,6)-tetrakisphosphate Biosynthesis	0.12162	SET,CA3,ATP,ACP1,ACP5,RASA1
Phagosome Maturation	0.12162	CTSV,PRDX1,LPO,CTSG,CTSE,DYNLRB1

Airway Inflammation in Asthma	0.12303	IL13
Diphthamide Biosynthesis	0.12303	ATP
PRPP Biosynthesis I	0.12303	ATP
Lipoate Biosynthesis and Incorporation II	0.12303	ATP
Methylmalonyl Pathway	0.12303	ATP
Inosine-5'-phosphate Biosynthesis II	0.12303	ATP
GDP-L-fucose Biosynthesis II (from L-fucose)	0.12303	ATP
Biotin-carboxyl Carrier Protein Assembly	0.12303	ATP
Asparagine Biosynthesis I	0.12303	ATP
N-acetylglucosamine Degradation II	0.12303	ATP
4-hydroxybenzoate Biosynthesis	0.12303	ATP
S-adenosyl-L-methionine Biosynthesis	0.12303	ATP
Role of BRCA1 in DNA Damage Response	0.12503	TP53,RB1,IFNG,PLK1
Tumoricidal Function of Hepatic Natural Killer Cells	0.12706	CASP3,LYVE1
TCA Cycle II (Eukaryotic)	0.12706	ATP,MDH1
Ethanol Degradation IV	0.12706	ATP,CAT
Amyloid Processing	0.12735	MAPK14,MAPK1,PRKACA
Gαs Signaling	0.12882	SRC,ATP,MAPK1,PRKACA,MAP2K1
Estrogen-mediated S-phase Entry	0.14488	RB1,ESR1
Tryptophan Degradation III (Eukaryotic)	0.14488	PARK7,CA1
Embryonic Stem Cell Differentiation into Cardiac Lineages	0.14555	NANOG
Glutathione Biosynthesis	0.14555	ATP
Uridine-5'-phosphate Biosynthesis	0.14555	ATP
L-glutamine Biosynthesis II (tRNA-dependent)	0.14555	ATP
Acetate Conversion to Acetyl-CoA	0.14555	ATP
PCP pathway	0.1574	MAP2K4,JUN,MAPK8
Actin Nucleation by ARP-WASP Complex	0.16368	GRB2,KRAS,NCK1
Cellular Effects of Sildenafil (Viagra)	0.1652	PDE2A,ATP,PDE3A,PRKACA,PDE5A
Creatine-phosphate Biosynthesis	0.16788	ATP
5-aminoimidazole Ribonucleotide Biosynthesis I	0.16788	ATP
Coenzyme A Biosynthesis	0.16788	ATP
Lysine Degradation V	0.16788	ATP

2-oxobutanoate Degradation I	0.16788	ATP
Galactose Degradation I (Leloir Pathway)	0.16788	ATP
Aspartate Degradation II	0.16788	MDH1
Folate Polyglutamylation	0.16788	ATP
HIPPO signaling	0.16788	YWHAE,SMAD3,YWHAZ,SKP1
Nur77 Signaling in T Lymphocytes	0.17022	CASP3,CD80,BCL2
Unfolded protein response	0.17022	HSPA8,MAPK8,BCL2
Cytotoxic T Lymphocyte-mediated Apoptosis of Target Cells	0.17219	CASP3,BCL2
D-myo-inositol-5-phosphate Metabolism	0.17458	SET,CA3,ATP,ACP1,ACP5,RASA1
Allograft Rejection Signaling	0.1766	IFNG,CD80,IL2
PXR/RXR Activation	0.18323	CYP3A4,PRKACA,IL6
Airway Pathology in Chronic Obstructive Pulmonary Disease	0.18923	MMP9
Urea Cycle	0.18923	ATP
CMP-N-acetylneuraminate Biosynthesis I (Eukaryotes)	0.18923	ATP
NAD Salvage Pathway III	0.18923	ATP
Selenocysteine Biosynthesis II (Archaea and Eukaryotes)	0.18923	ATP
Inositol Pyrophosphates Biosynthesis	0.18923	ATP
Molybdenum Cofactor Biosynthesis	0.18923	ATP
GDP-mannose Biosynthesis	0.18923	GPI
Netrin Signaling	0.18967	PRKACA,NCK1,RAC3
Ethanol Degradation II	0.20045	AKR1A1,ATP
Mitotic Roles of Polo-Like Kinase	0.20277	TGFB1,PLK1,CCNB1
Calcium-induced T Lymphocyte Apoptosis	0.20941	PRKCD,PRKCB,PRKCA
Role of Wnt/GSK-3β Signaling in the Pathogenesis of Influenza	0.20941	IFNG,IFNB1,IFNAR1
Tight Junction Signaling	0.20989	EPB41,JUN,TGFB1,PRKACA,JAM2,SPTAN1
Dopamine-DARPP32 Feedback in cAMP Signaling	0.20989	ATP,PRKCD,CAMKK1,PRKACA,PRKCB,PRKCA
Estrogen Biosynthesis	0.20989	CYP3A4,HSD17B1
Pathogenesis of Multiple Sclerosis	0.21038	CXCL9
Arginine Biosynthesis IV	0.21038	ATP
Sucrose Degradation V (Mammalian)	0.21038	ATP
Citrulline-Nitric Oxide Cycle	0.21038	ATP
Calcium Signaling	0.21528	ATP,CAMK1D,MAPK1,CAMKK1,PRKACA,TPM1,TPM4

CTLA4 Signaling in Cytotoxic T Lymphocytes	0.2208	CD80,GRB2,GRAP2,PIK3CG
TWEAK Signaling	0.22909	CASP3,BIRC3
Citrulline Biosynthesis	0.23067	ATP
Folate Transformations I	0.23067	ATP
Calcium Transport I	0.25061	ATP
Salvage Pathways of Pyrimidine Deoxyribonucleotides	0.25061	ATP
Assembly of RNA Polymerase III Complex	0.2704	BRF1
Fatty Acid Activation	0.28907	ATP
Methylglyoxal Degradation III	0.28907	AKR1A1
Autoimmune Thyroid Disease Signaling	0.3062	CD80,IL2
3-phosphoinositide Degradation	0.3069	SET,CA3,ACP1,ACP5,RASA1
DNA Double-Strand Break Repair by Homologous Recombination	0.30761	ABL1
DNA Double-Strand Break Repair by Non-Homologous End Joining	0.30761	XRCC6
Mevalonate Pathway I	0.30761	ATP
Leucine Degradation I	0.30761	ATP
Maturity Onset Diabetes of Young (MODY) Signaling	0.32509	ATP
Urate Biosynthesis/Inosine 5'-phosphate Degradation	0.32509	IMPDH2
γ-glutamyl Cycle	0.32509	ATP
Granzyme B Signaling	0.34277	CASP3
Mismatch Repair in Eukaryotes	0.34277	ATP
Dermatan Sulfate Degradation (Metazoa)	0.34277	IDS
Phenylalanine Degradation IV (Mammalian, via Side Chain)	0.34277	ATP
Superpathway of Citrulline Metabolism	0.3767	ATP
γ-linolenate Biosynthesis II (Animals)	0.3767	ATP
1D-myo-inositol Hexakisphosphate Biosynthesis II (Mammalian)	0.3767	ATP
D-myo-inositol (1,3,4)-trisphosphate Biosynthesis	0.3767	ATP
Superpathway of Geranylgeranyldiphosphate Biosynthesis I (via Mevalonate)	0.3767	ATP
Polyamine Regulation in Colon Cancer	0.40832	KRAS

Tryptophan Degradation X (Mammalian, via Tryptamine)	0.40832	AKR1A1
Valine Degradation I	0.40832	HIBADH
Histamine Degradation	0.40832	ATP
Oxidative Ethanol Degradation III	0.40832	ATP
Role of CHK Proteins in Cell Cycle Checkpoint Control	0.40926	TP53,PLK1
Bupropion Degradation	0.42364	CYP3A4
Purine Nucleotides Degradation II (Aerobic)	0.42364	IMPDH2
Methionine Degradation I (to Homocysteine)	0.42364	ATP
Glutathione Redox Reactions I	0.43853	GSTP1
Superpathway of D-myo-inositol (1,4,5)-trisphosphate Metabolism	0.4529	ATP
D-myo-inositol (1,4,5)-Trisphosphate Biosynthesis	0.46774	ATP
Cysteine Biosynthesis III (mammalia)	0.46774	ATP
Acetone Degradation I (to Methylglyoxal)	0.48084	CYP3A4
G Protein Signaling Mediated by Tubby	0.50816	ABL1
Glutathione-mediated Detoxification	0.50816	GSTP1
B Cell Development	0.5445	CD80
Circadian Rhythm Signaling	0.55719	VIP
Superpathway of Cholesterol Biosynthesis	0.55719	ATP
Fatty Acid β-oxidation I	0.56885	ATP
Dopamine Receptor Signaling	0.57412	ATP,PRKACA
MIF-mediated Glucocorticoid Regulation	0.57943	MAPK1
Role of RIG1-like Receptors in Antiviral Innate Immunity	0.60117	IFNB1
Nicotine Degradation III	0.60117	CYP3A4
Noradrenaline and Adrenaline Degradation	0.60117	AKR1A1
Cell Cycle Regulation by BTG Family Proteins	0.61094	RB1
Antigen Presentation Pathway	0.62087	IFNG
Regulation of Actin-based Motility by Rho	1	PAK6,RAC3
RhoA Signaling	1	EPHA1
Melatonin Degradation I	1	CYP3A4
Phospholipases	1	PAFAH1B2
tRNA Charging	1	ATP

Nicotine Degradation II	1	CYP3A4
Serotonin Degradation	1	AKR1A1
Superpathway of Melatonin Degradation	1	CYP3A4
Stearate Biosynthesis I (Animals)	1	ATP
Superpathway of Methionine Degradation	1	ATP
Remodeling of Epithelial Adherens Junctions	1	SRC
Oxidative Phosphorylation	1	ATP,ATP5F1B
Wnt/Ca+ pathway	1	PRKCA
GABA Receptor Signaling	1	UBB,RPS27A
Phototransduction Pathway	1	PRKACA
Eicosanoid Signaling	1	PAFAH1B2

Publisher's Note: MDPI stays neutral with regard to jurisdictional claims in published maps and institutional affiliations.

© 2020 by the authors. Submitted for possible open access publication under the terms and conditions of the Creative Commons Attribution (CC BY) license (http://creativecommons.org/licenses/by/4.0/).
